# Non‐peroxisomal allantoin biosynthesis reveals unexpected flexibility in plant purine catabolism

**DOI:** 10.1111/tpj.71178

**Published:** 2026-10-23

**Authors:** Yuta Takeuchi, Tamio Sumida, Hiroshi Shimada, Atsushi Sakamoto

**Affiliations:** ^1^ Program of Mathematical and Life Sciences, Division of Integrated Sciences for Life, Graduate School of Integrated Sciences for Life Hiroshima University 1‐3‐1 Kagamiyama Higashi‐Hiroshima 739‐8526 Japan; ^2^ Department of Biological Science, School of Science Hiroshima University 1‐3‐1 Kagamiyama Higashi‐Hiroshima 739‐8526 Japan

**Keywords:** allantoin synthase, alternative splicing, enzyme evolution, subcellular localization, peroxisome, urate

## Abstract

Allantoin, a central intermediate of purine catabolism, plays key roles in nitrogen remobilization and is also implicated in stress protection in plants. As in other eukaryotes, allantoin biosynthesis in land plants has long been considered restricted to peroxisomes, where this process is catalyzed by ALLANTOIN SYNTHASE (ALNS). However, angiosperms produce not only the canonical peroxisomal isoform but also a shorter splice variant lacking a type 2 peroxisomal targeting signal (PTS2), raising the question of whether allantoin biosynthesis can occur outside peroxisomes. Through comparative sequence analyses across Archaeplastida, we show that PTS2‐lacking ALNS exhibits a patchy distribution within Viridiplantae, including species without identifiable PTS2‐containing ALNS homologs, suggesting the evolutionary significance of non‐peroxisomal allantoin biosynthesis. Fluorescent protein fusion analysis showed that PTS2‐lacking ALNS proteins from the angiosperm *Arabidopsis thaliana*, the monilophyte *Dryopteris erythrosora*, and the bryophyte *Marchantia polymorpha* localize outside peroxisomes, consistent with cytosolic localization. Furthermore, recombinant proteins from all three species catalyzed stereospecific (*S*)‐allantoin formation. *Arabidopsis alns* knockout mutants accumulated racemic allantoin, indicating that ALNS ensures the stereospecific formation of (*S*)‐allantoin while preventing the generation of poorly metabolizable (*R*)‐allantoin, which likely arises through spontaneous decomposition of the unstable ALNS substrate 5‐hydroxyisourate. Importantly, expression of either ALNS isoform restored normal allantoin metabolism and largely rescued the growth and physiological defects of the *alns* mutants. These findings demonstrate the functional equivalence of cytosolic and peroxisomal ALNS in *Arabidopsis* and reveal previously unrecognized flexibility in the subcellular organization of purine catabolism in plants.

## Significance Statement

Allantoin biosynthesis in plants, and more broadly across eukaryotes, is generally considered to be a peroxisomal process because the substrate for allantoin synthase is produced in this compartment. We show that a cytosolic isoform of this enzyme alone, generated by alternative splicing, functions in allantoin biosynthesis, revealing unexpected metabolic plasticity in an established plant pathway.

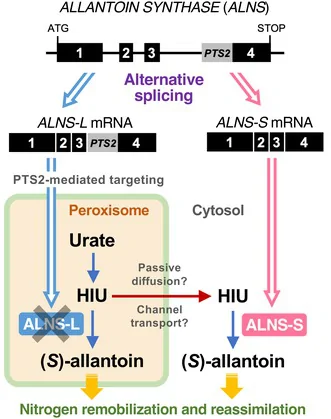

## INTRODUCTION

Purine degradation is a conserved nitrogen metabolic process that occurs across all domains of life, yet its final products have diverged during evolution. In terrestrial animals, progressive truncation of the purine degradation pathway favored the excretion of relatively non‐toxic nitrogenous wastes such as urate and allantoin (White et al., 1973). By contrast, land plants have retained the complete degradative route to ammonium, enabling the recovery of purine‐derived nitrogen for reassimilation into organic compounds (Schubert & Boland, 1990). Thus, purine degradation is not merely catabolic but also contributes to nutrient recycling and nitrogen‐use efficiency in plants.

In angiosperms such as Arabidopsis (*Arabidopsis thaliana*), the purine degradation pathway proceeds through three subcellular compartments (for review, see Werner & Witte, 2011). As shown in Figure 1, the early reactions occur in the cytosol, where purine nucleotides, nucleosides, and free purine bases converge at xanthine as a common intermediate. Xanthine is oxidized by xanthine dehydrogenase (XDH) to urate, which is transported into peroxisomes for further degradation. In the peroxisome, urate oxidase (UOX) produces 5‐hydroxyisourate (HIU), an unstable intermediate that is converted to allantoin via 2‐oxo‐4‐hydroxy‐4‐carboxy‐5‐ureidoimidazoline (OHCU). This two‐step conversion is catalyzed by allantoin synthase (ALNS), a bifunctional enzyme with HIU hydrolase (HIUH) and OHCU decarboxylase (OHCUD) activities (Lamberto et al., 2010; Pessoa et al., 2010). Allantoin is then metabolized in the endoplasmic reticulum by allantoinase (ALN) and additional enzymes, ultimately releasing ammonium for reassimilation. Arabidopsis and rice (*Oryza sativa*) mutants defective in steps of this pathway frequently exhibit nitrogen deficiency phenotypes, especially under nitrogen limitation or during late development (Brychkova et al., 2008; Han et al., 2020; Nakagawa et al., 2007a; Soltabayeva et al., 2018; Takagi et al., 2018).

**Figure 1:**
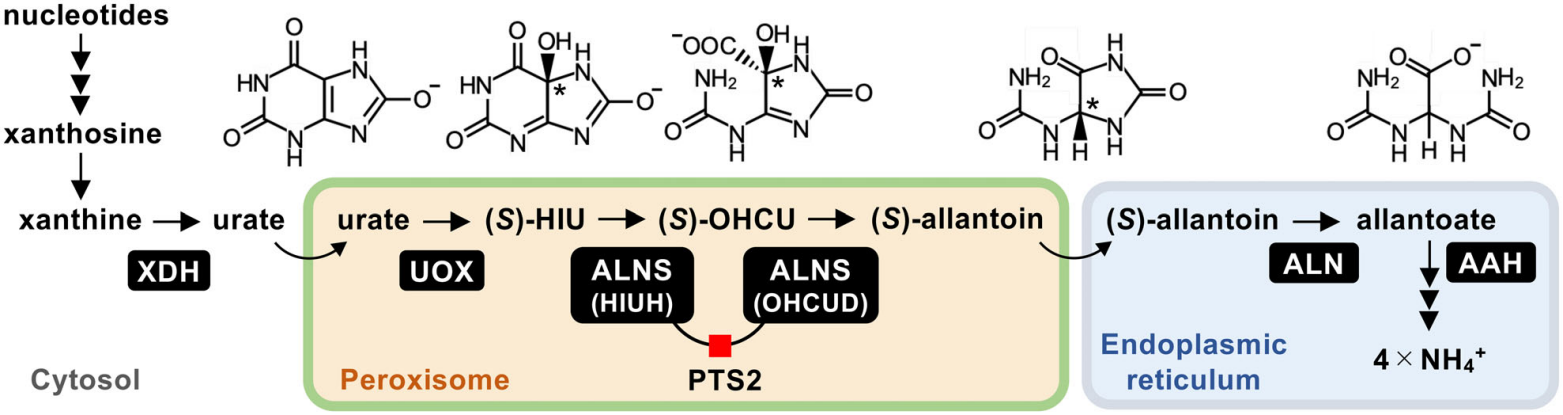
Simplified scheme of the major purine catabolic pathway in plants. Xanthine is converted to urate by xanthine dehydrogenase (XDH) and then to (*S*)‐5‐hydroxyisourate [(*S*)‐HIU] by urate oxidase (UOX). (*S*)‐HIU is hydrolyzed to (*S*)‐2‐oxo‐4‐hydroxy‐4‐carboxy‐5‐ureidoimidazoline [(*S*)‐OHCU] by the HIU hydrolase (HIUH) domain of the bifunctional allantoin synthase (ALNS), and (*S*)‐OHCU is subsequently decarboxylated by the OHCU decarboxylase (OHCUD) domain of ALNS to yield (*S*)‐allantoin. (*S*)‐allantoin is further metabolized by allantoinase (ALN) and allantoate amidohydrolase (AAH). The structures shown correspond to the enzymatically generated *S*‐enantiomers of HIU, OHCU, and allantoin (Pipolo et al., 2011; Ramazzina et al., 2006). Asterisks (*) indicate chiral centers. PTS2, type 2 peroxisomal targeting signal.

Among the intermediates of the purine degradation pathway, allantoin has attracted considerable attention as a key player in purine‐derived nitrogen recycling and remobilization. Allantoin and allantoate constitute the major transported ureides in symbiotic legumes (Matsumoto et al., 1977), whereas their roles in non‐legumes have remained less clear. Nevertheless, recent mutant studies of catabolic enzymes and ureide permeases suggested that allantoin contributes to long‐distance nitrogen translocation in non‐leguminous species, including Arabidopsis and rice (Lescano et al., 2020; Redillas et al., 2019; Soltabayeva et al., 2018; Takagi et al., 2018).

Beyond its roles in nitrogen metabolism, allantoin has also been implicated in plant–environment interactions. Allantoin accumulates under various stress conditions and altered allantoin levels have been associated with stress tolerance (Brychkova et al., 2008; Lescano et al., 2016; Nourimand & Todd, 2016; Watanabe et al., 2010, 2014). In addition, this metabolite has been linked to seed dormancy in Arabidopsis (Piskurewicz et al., 2016) and was identified as a root‐secreted kin recognition factor in rice (Yang et al., 2018). Thus, allantoin appears to participate in nitrogen metabolism and in diverse physiological processes. Understanding how its biosynthesis and turnover are regulated is therefore of particular interest.

Allantoin biosynthesis in plants exhibits two notable evolutionary features. First, ALNS arose through the fusion of HIUH and OHCUD into a single bifunctional protein containing a peroxisomal targeting signal (PTS), specifically a type 2 signal (PTS2), between the two catalytic domains (Ramazzina et al., 2006; Reumann et al., 2007). By contrast, in many other organisms, the two enzymes are encoded separately. Second, in angiosperms, alternative splicing of the exon containing the PTS2‐coding region generates a shorter isoform lacking this targeting signal (Lamberto et al., 2010). This isoform, originally identified as a transthyretin‐like protein (TTL) and implicated in brassinosteroid signaling (Nam & Li, 2004), retains enzymatic activity *in vitro* (Lamberto et al., 2010; Pessoa et al., 2010), yet its metabolic role in plants remains unresolved. A recent comparative sequence analysis in algae suggested that, before bifunctional ALNS evolved, ancestral HIUH and OHCUD lacked PTS motifs and were likely cytosolic (Kechasov et al., 2020). In addition, several extant algal lineages with secondary plastids encode ALNS proteins lacking obvious targeting signals (Kechasov et al., 2020; Oh et al., 2018). These observations support the idea that allantoin biosynthesis may not be strictly confined to peroxisomes in all lineages. However, the evolutionary history and diversification of ALNS and other enzymes involved in allantoin biosynthesis across Archaeplastida remain incompletely understood, particularly within green plant lineages.

Another unresolved issue is that Arabidopsis *alns* knockout mutants do not show reduced allantoin levels; instead, accumulating allantoin at levels comparable to wild‐type (WT) plants (Chen et al., 2020). This paradox suggests that allantoin accumulation cannot be explained solely by the canonical ALNS pathway.

In this study, we combined comparative sequence analysis across Archaeplastida with biochemical, cell biological, and genetic analyses to investigate the evolutionary and physiological significance of ALNS diversification. Comparative analyses revealed a patchy distribution of PTS2‐lacking ALNS proteins across green plant lineages, including several taxa lacking identifiable PTS2‐containing homologs. Biochemical and localization analyses of such PTS2‐lacking ALNS proteins from selected bryophyte and monilophyte species demonstrated that these proteins catalyze stereospecific (*S*)‐allantoin formation and localize outside peroxisomes, supporting the occurrence of non‐peroxisomal allantoin biosynthesis in land plants. Functional analyses in Arabidopsis further demonstrated that the cytosolic and peroxisomal isoforms function equivalently in allantoin metabolism. Finally, our analyses provide a mechanistic explanation for the paradoxical accumulation of allantoin in the *alns* mutants. Together, our findings reveal unexpected flexibility in both the evolution and subcellular compartmentation of allantoin biosynthesis in plants.

## RESULTS

### Certain Archaeplastida lineages possess only PTS2‐lacking ALNS proteins

To investigate the evolutionary diversification of allantoin‐biosynthetic enzymes in Archaeplastida, we compared the domain architectures of ALNS and their ancestral HIUH and OHCUD enzymes across major lineages, including the presence or absence of PTS motifs. In addition to 26 previously reported sequences (Kechasov et al., 2020; Lamberto et al., 2010; Oh et al., 2018; Reumann et al., 2007), we retrieved 61 additional sequences from the National Center for Biotechnology Information (NCBI), Phytozome (Goodstein et al., 2012), and the China National GeneBank using the two Arabidopsis ALNS isoforms as queries. The two Arabidopsis isoforms were previously designated TTL^1−^ (for PTS2‐containing) and TTL^2−^ (for PTS2‐lacking) (Lamberto et al., 2010). Consistent with the notation in The Arabidopsis Information Resource (TAIR) and their structural differences, we refer to these isoforms as AtALNS‐L (for PTS2‐containing long form) and AtALNS‐S (for PTS2‐lacking short form) hereafter. In total, we compiled a list of 87 sequences spanning major Archaeplastida lineages (Table S1), including 11 angiosperms, 14 gymnosperms, seven monilophytes, three lycophytes, 11 bryophytes, seven streptophyte algae, five chlorophytes, four rhodophytes, and three glaucophytes.

Figure 2 summarizes the evolutionary distribution of allantoin‐synthesizing enzymes across Archaeplastida (see also Table S1). Consistent with previous reports (Lamberto et al., 2010), all angiosperms examined possessed both ALNS‐L and ALNS‐S, while gymnosperms possessed only ALNS‐L. Among seedless vascular plants, lycophytes also contained only ALNS‐L. Monilophytes, which are phylogenetically closer to seed plants, contained either ALNS‐L or ALNS‐S, but no species examined harbored both isoforms. A mutually exclusive pattern was also observed among bryophytes and streptophyte algae. Within Viridiplantae, the chlorophyte lineage was unique in that it had retained both ALNS‐L and the ancestral enzymes HIUH and OHCUD. These ancestral enzymes either lacked PTS motifs or carried a PTS in only one enzyme (HIUH with PTS2 or OHCUD with type 1 PTS [PTS1]). No ALNS‐S–type protein was detected in any green alga. By contrast, non‐green algae (rhodophytes and glaucophytes) appeared to lack bifunctional enzymes altogether. Rhodophyte proteins lacked PTS motifs, whereas glaucophyte proteins contained these motifs, although the available sequence data remain limited.

**Figure 2:**
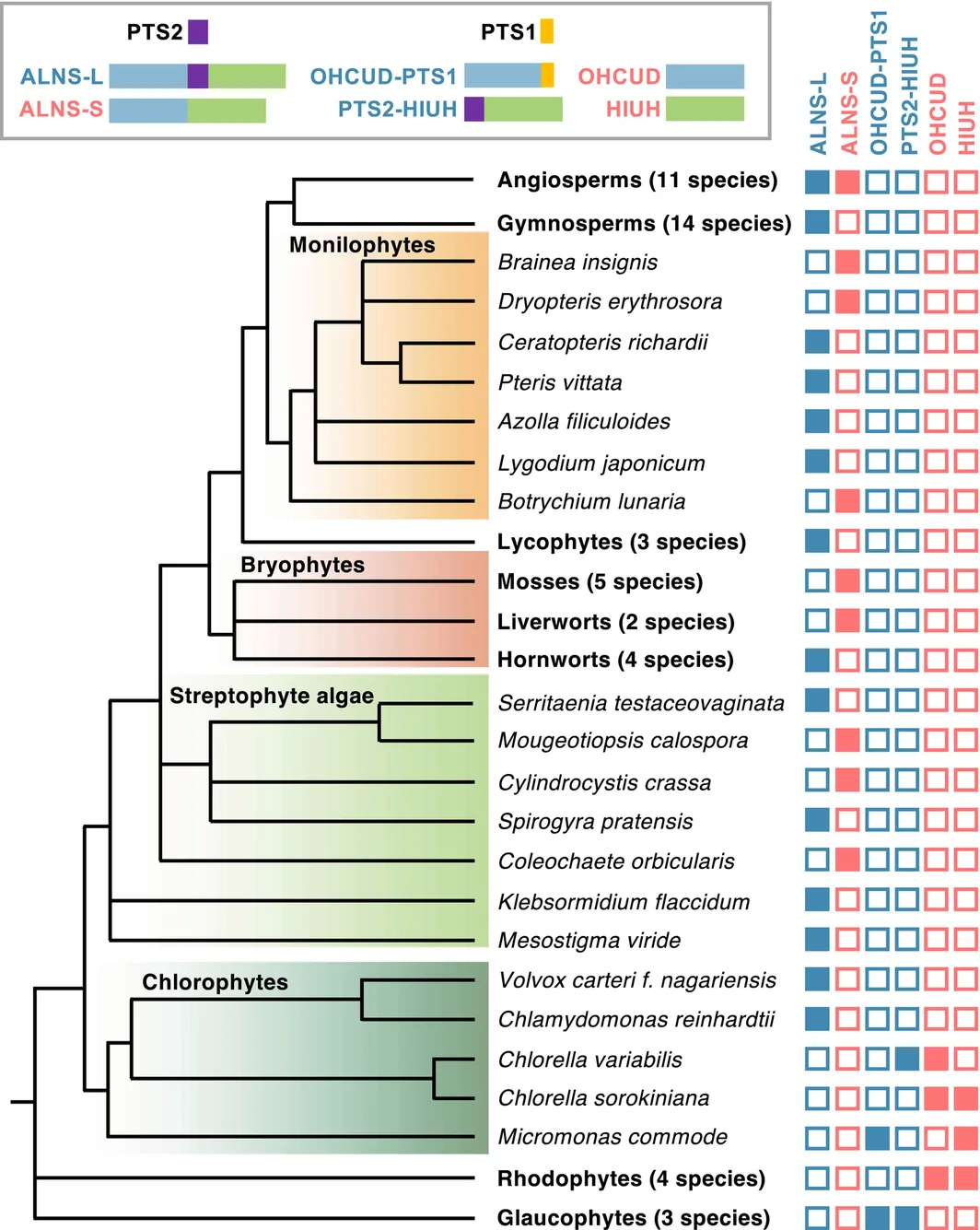
Domain architecture and evolutionary distribution of enzymes involved in allantoin biosynthesis across Archaeplastida lineages. The top panel shows schematic representations of ALNS‐L, ALNS‐S, OHCUD‐PTS1, PTS2‐HIUH, OHCUD, and HIUH, together with their domain organization and peroxisomal targeting signals (PTSs): HIUH (light green), OHCUD (light blue), PTS1 (yellow), and PTS2 (dark purple). The distribution of these enzymes across major lineages is indicated by filled (presence) or open (absence) squares. Enzymes containing PTS motifs are shown in blue, whereas PTS‐lacking enzymes are shown in pink. Sequence sources are listed in Table S1. The phylogenetic tree was generated using phyloT v2 (https://phylot.biobyte.de/).

Collectively, these results reveal substantial diversity in allantoin‐biosynthetic enzymes across Archaeplastida, as reflected in differences in domain architecture, predicted subcellular localizations, and lineage‐specific enzyme repertoires. Notably, species in which only ALNS‐S was identified also contained identifiable XDH and UOX homologs, except for the moss *Sanionia uncinata*, from which no UOX homolog was retrieved (Tables S2 and S3). The absence of identifiable ALNS‐L homologs in these taxa raises the possibility that allantoin biosynthesis occurs outside peroxisomes.

### PTS2‐lacking ALNS proteins from an angiosperm, a monilophyte, and a bryophyte localize outside peroxisomes

ALNS‐L was experimentally shown to localize to peroxisomes, whereas ALNS‐S has been observed in the cytosol in both Arabidopsis and rice (Lamberto et al., 2010; Reumann et al., 2007; Yu et al., 2024). However, with the exception of the proteomic identification of peroxisomal AtALNS‐L (Reumann et al., 2007), these observations were based on transient expression in protoplasts of the native species or in tissues of heterologous species. It thus remained unclear whether these localization patterns are maintained under stable expression *in planta*. To address this issue, we generated transgenic Arabidopsis lines stably expressing each AtALNS isoform as an N‐terminal fusion to enhanced green fluorescent protein (EGFP) (Figure S1A,C). In cotyledons, EGFP–AtALNS‐S fluorescence did not co‐localize with that of transiently expressed mCherry fused with PTS1 (mCherry–PTS1), suggesting that AtALNS‐S resides in the cytosol (Figure 3a). Consistent with this interpretation, EGFP–AtALNS‐S showed diffuse cytosolic fluorescence in young root cells stained with FM4‐64 (Figure 3b). By contrast, EGFP–AtALNS‐L co‐localized with mCherry–PTS1, confirming its peroxisomal localization (Figure 3c). Transient expression of an *EGFP*‐fused genomic *AtALNS* construct in WT cotyledons corroborated these results, revealing dual localization to both peroxisomes and the cytosol (Figure 3d).

**Figure 3:**
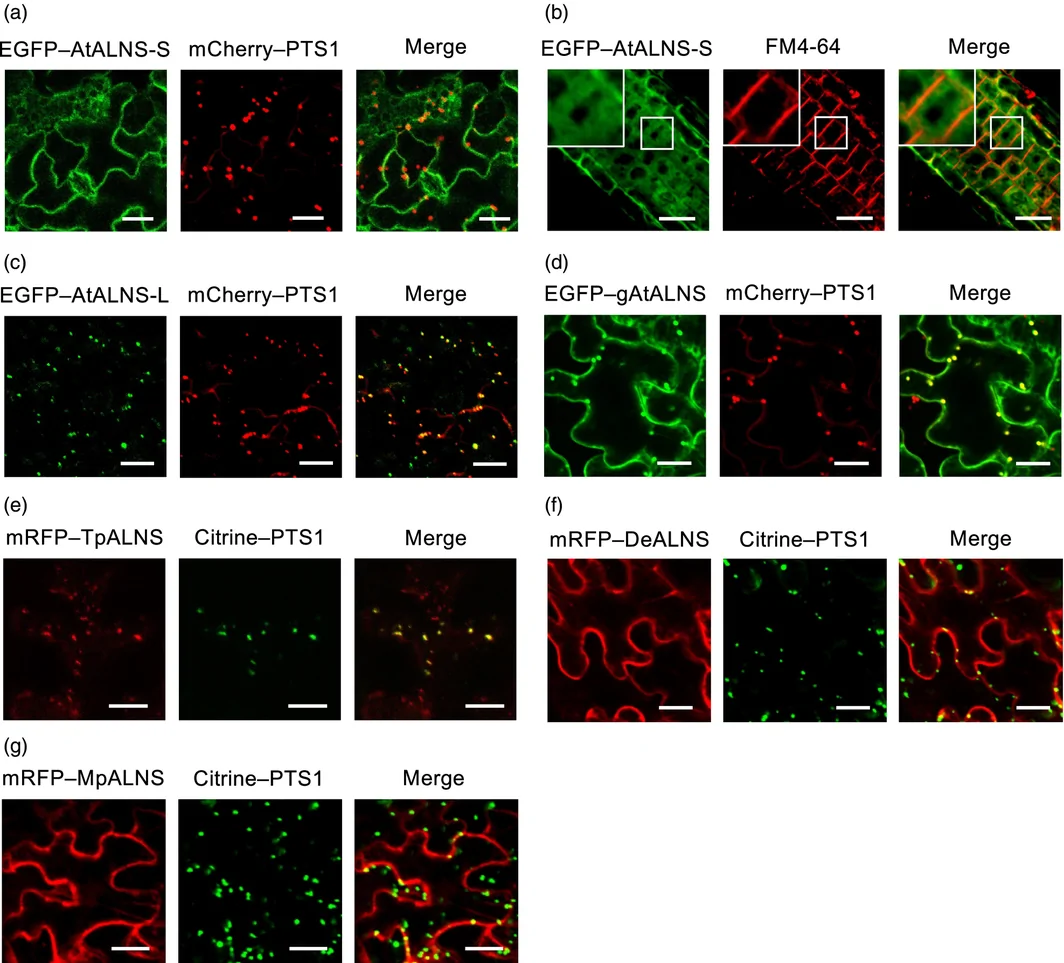
Subcellular localization of EGFP‐ or mRFP‐fused ALNS proteins from an angiosperm (*Arabidopsis thaliana*), a gymnosperm (*Thuja plicata*), a monilophyte (*Dryopteris erythrosora*), and a bryophyte (*Marchantia polymorpha*). Representative confocal images of Arabidopsis are shown. (a) Six‐day‐old Arabidopsis cotyledons stably expressing EGFP–AtALNS‐S and transiently co‐expressing mCherry–PTS1. (b) Two‐day‐old Arabidopsis roots stably expressing EGFP–AtALNS‐S, stained with FM4‐64 to visualize the plasma membrane and internal membranes. (c) Six‐day‐old Arabidopsis cotyledons stably expressing EGFP–AtALNS‐L and transiently co‐expressing mCherry–PTS1. (d) Nine‐day‐old Arabidopsis cotyledons transiently co‐expressing EGFP–gAtALNS and mCherry–PTS1. (e) Leaf epidermal cells of 21‐day‐old Arabidopsis seedlings transiently co‐expressing mRFP–TpALNS and Citrine–PTS1. (f) Leaf epidermal cells of 21‐day‐old Arabidopsis seedlings transiently co‐expressing mRFP–DeALNS and Citrine–PTS1. (g) Leaf epidermal cells of 21‐day‐old Arabidopsis seedlings transiently co‐expressing mRFP–MpALNS and Citrine–PTS1. Scale bars, 20 μm. See Figure S1 for details about the ALNS fusion constructs and fluorescent peroxisomal reporters.

We next examined the subcellular localization of ALNS from the gymnosperm western red cedar (*Thuja plicata*; TpALNS), the monilophyte *Dryopteris erythrosora* (DeALNS), and the bryophyte *Marchantia polymorpha* (MpALNS). TpALNS possessed a recognizable PTS2 motif, whereas DeALNS and MpALNS lacked any predicted PTS (Table S1). We transiently co‐expressed monomeric red fluorescent protein (mRFP)‐fused TpALNS, DeALNS, or MpALNS with the peroxisomal marker Citrine–PTS1 in Arabidopsis leaves (Figure S1B). The mRFP–TpALNS signal overlapped with that of Citrine–PTS1, whereas mRFP–DeALNS and mRFP–MpALNS showed no such overlap (Figure 3e–g). These results indicate that the gymnosperm enzyme localizes to peroxisomes, whereas the monilophyte and bryophyte enzymes reside outside peroxisomes, most likely in the cytosol.

Together, these observations support the sequence‐based predictions (Figure 2) and reveal the lineage‐specific diversification of the subcellular localization of ALNS during land‐plant evolution.

### Recombinant PTS2‐lacking ALNS proteins from a monilophyte and a bryophyte catalyze stereospecific allantoin formation

To date, among PTS2‐lacking ALNS proteins from Archaeplastida, only AtALNS‐S has been biochemically characterized (Lamberto et al., 2010; Pessoa et al., 2010). We therefore examined the enzymatic activities of purified recombinant MpALNS and DeALNS, using recombinant AtALNS‐S as a positive control, in a UOX‐coupled assay in which HIU was generated *in situ* (Figure 4). Both MpALNS and DeALNS accelerated UOX‐catalyzed urate oxidation and exhibited robust HIU‐hydrolyzing activities comparable to those of AtALNS‐S (Figure 4b,c). Time‐resolved circular dichroism (CD) spectra further showed that both proteins catalyzed the stereospecific conversion of HIU to (*S*)‐allantoin. In reactions containing MpALNS, DeALNS, or AtALNS‐S, the HIU‐associated CD signal at 312 nm was markedly decreased and the OHCU signal at 257 nm was evident at 3 min. By 9 min, the OHCU signal had largely disappeared, leaving spectral features consistent with the formation of (*S*)‐allantoin (Figure 4d); this assignment was confirmed by comparison with the authentic (*S*)‐allantoin obtained by chiral separation of a racemic allantoin standard (Figure S2). By contrast, the HIU‐associated signal remained clearly detectable at 9 min in the UOX‐only control, although it decreased over time. These results demonstrate that PTS2‐lacking ALNS proteins from a monilophyte and a bryophyte catalyze stereospecific allantoin formation. Together, these findings extend biochemical evidence for canonical ALNS activity to non‐angiosperm Archaeplastida and support the occurrence of non‐peroxisomal allantoin biosynthesis across diverse land‐plant lineages.

**Figure 4:**
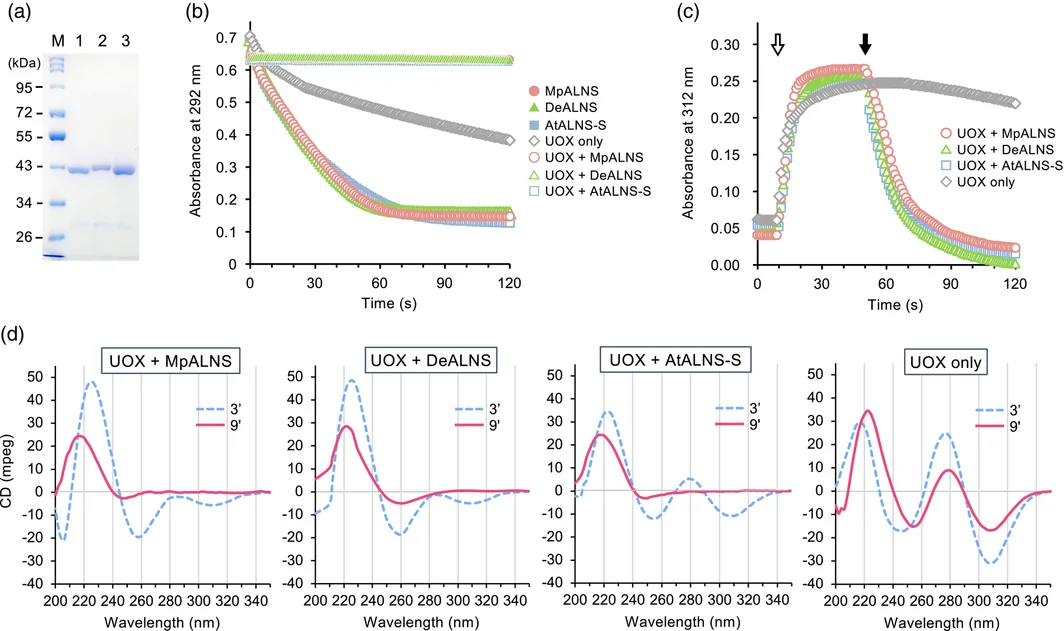
Enzyme activities and stereospecific allantoin formation by recombinant PTS2‐lacking ALNS proteins from a bryophyte (*Marchantia polymorpha*), a monilophyte (*Dryopteris erythrosora*), and an angiosperm (*Arabidopsis thaliana*). (a) Coomassie Brilliant Blue staining of purified recombinant proteins (2 μg) separated by 10% SDS–PAGE under denaturing conditions. Lane M, protein molecular mass markers (kDa); lane 1, *M. polymorpha* ALNS (MpALNS); lane 2, *D. erythrosora* ALNS (DeALNS); lane 3, *A. thaliana* ALNS‐S (AtALNS‐S). (b) Acceleration of UOX‐catalyzed urate oxidation by the recombinant ALNS proteins. Reactions were initiated by adding 50 μM urate, and urate degradation was monitored by the decrease in absorbance at 292 nm. (c) HIUH activity of the recombinant ALNS proteins. HIU was generated by adding UOX (open arrow) to a reaction mixture containing 50 μM urate. When the absorbance at 312 nm reached its maximum, purified ALNS proteins were added (closed arrow), and HIU degradation was monitored by the decrease in absorbance. (d) Time‐resolved CD spectra of urate oxidation products generated by UOX in the presence or absence of recombinant ALNS proteins. Spectra were recorded at 3 and 9 min after the addition of 100 μM urate. The negative CD peaks at 312 and 257 nm correspond to HIU and OHCU, respectively (Lamberto et al., 2010; Ramazzina et al., 2006). See Figure S2 for the reference CD spectrum of (*S*)‐allantoin. Representative data from three independent assays are shown in (b–d).

### *AtALNS‐S* transcripts are more abundant than *AtALNS‐L* transcripts in Arabidopsis

To assess the potential functional significance of ALNS‐S in Arabidopsis, we analyzed the relative abundance of *AtALNS* splice variants. Analysis of PastDB, a transcriptome‐based database of alternative splicing events and transcript variant usage (Martín et al., 2021), showed that the *AtALNS* locus produces three mRNA variants, *AtALNS‐L*, *AtALNS‐S*, and an intron‐retained form, through alternative splicing at intron 3 (Figure S3). Across more than 60 transcriptome datasets spanning diverse tissues, organs, and developmental stages, *AtALNS‐S* was consistently the most abundant transcript variant, accounting for approximately 60–80% of total *AtALNS* transcripts. *AtALNS‐L* transcripts represented roughly 20–40%, whereas the intron‐retained form was always the least abundant, typically constituting less than 10% of total *AtALNS* transcripts. These transcript usage profiles indicate that AtALNS‐S likely constitutes the predominant functional output of the *ALNS* locus in Arabidopsis.

### The loss of *AtALNS* leads to racemic allantoin accumulation in Arabidopsis

The non‐peroxisomal localization, predominant expression, and enzymatic activity of AtALNS‐S prompted us to examine how the loss of ALNS activity would affect allantoin metabolism *in planta*. We therefore compared allantoin levels in the *ALNS* knockout mutant *alns‐1* (Figure S4A) with those in WT plants. While *alns‐1* seedlings were previously reported to accumulate allantoin at levels similar to those in WT rather than reduced levels (Chen et al., 2020), we found that mature *alns‐1* plants accumulated approximately seven‐fold more allantoin than WT plants (Figure 5a).

**Figure 5:**
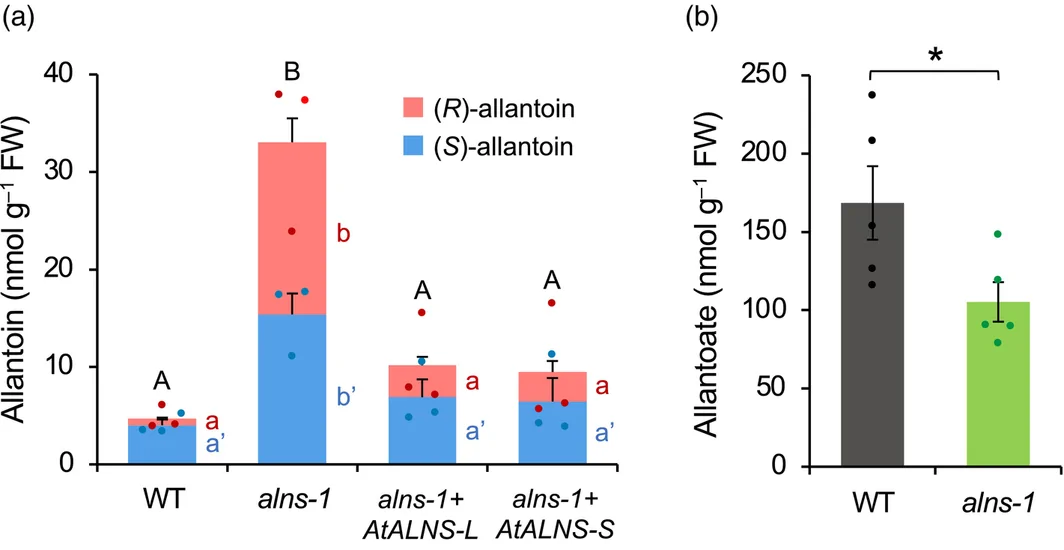
Both *AtALNS* splice variants prevent the overaccumulation of racemic allantoin in the *alns‐1* mutant. (a) Total allantoin levels and enantiomeric composition in wild‐type (WT), *alns‐1*, and transgenic lines expressing *AtALNS‐L* or *AtALNS‐S* cDNA in the *alns‐1* background. (b) Allantoate levels in WT and *alns‐1* plants. Samples were collected from 32‐day‐old soil‐grown plants. Values represent means ± SEM of three (a) or five (b) biological replicates. In (a), different uppercase letters indicate significant differences in total allantoin content, whereas different lowercase letters indicate significant differences among genotypes within each enantiomeric form (pink, (*R*)‐allantoin; blue, (*S*)‐allantoin), based on one‐way ANOVA followed by Tukey's multiple comparison test (*P* < 0.05). In (b), an asterisk (*) indicates a significant difference from WT (Student's *t*‐test, *P* < 0.05).

To explain this paradoxical accumulation, we examined whether (*R*)‐allantoin accounts for the elevated total allantoin levels in the mutant. HIU, the substrate for ALNS (Figure 1), is chemically unstable and undergoes spontaneous decomposition to racemic allantoin *in vitro* (Kahn & Tipton, 2000), whereas downstream ALN enzymes in various organisms preferentially degrade (*S*)‐allantoin and exhibit much lower activity toward the non‐natural (*R*)‐enantiomer (Lee & Roush, 1964; Vogels et al., 1966). Accordingly, using the enantiomer assignments established from racemic allantoin standards (Figure S2), we analyzed the enantiomeric ratio of allantoin purified from plant tissues by preparative HPLC (Figure S5). This analysis revealed that WT plants predominantly contained (*S*)‐allantoin, whereas *alns‐1* accumulated a markedly increased proportion of the (*R*)‐enantiomer, slightly exceeding that of the (*S*)‐enantiomer (Figure 5a; *R*/*S* ratio = 1.1 in *alns‐1* and 0.17 in WT). A minor proportion of (*R*)‐allantoin was also detected in WT samples, although whether this reflects low‐level spontaneous formation *in planta* or partial racemization during sample preparation remains unclear. These results suggest that ALNS rapidly channels HIU into stereospecific (*S*)‐allantoin formation, thereby limiting spontaneous reactions that generate the metabolically disfavored (*R*)‐enantiomer.

Consistent with this interpretation, *alns‐1* plants exhibited significantly lower allantoate content than the WT (Figure 5b), despite higher *ALN* expression (Figure S6). To examine whether poorly metabolized (*R*)‐allantoin could interact with ALN, we performed molecular docking using the AlphaFold‐predicted model of Arabidopsis ALN (AtALN). The top‐ranked poses of the two enantiomers showed broadly comparable Vina scores (Table S4). Among the highly ranked solutions, both (*S*)‐ and (*R*)‐allantoin were accommodated within the same predicted substrate‐binding pocket in proximity to Asn140, Asp366, and Ser368, which correspond to substrate‐interacting residues identified in a bacterial allantoinase (Kim et al., 2009; Figure S7). Overlay of representative poses revealed broadly similar overall positioning of the two enantiomers, although the orientation of the hydantoin ring differed. Together, these results suggest that loss of ALNS leads to the substantial accumulation of (*R*)‐allantoin and reduced turnover of (*S*)‐allantoin, possibly involving (*R*)‐allantoin binding to ALN.

### Both *AtALNS* splice variants restore allantoin metabolism in *alns* mutants

Because the loss of ALNS disrupts allantoin metabolism and causes the substantial accumulation of the (*R*)‐enantiomer, we next asked whether either *ALNS* splice variant could restore metabolic homeostasis. We generated transgenic Arabidopsis lines in the *alns‐1* background expressing either *AtALNS‐L* or *AtALNS‐S* cDNA under the control of the native promoter (Figure S4B–D). Expression of either AtALNS isoform restored both total allantoin levels and the enantiomeric composition to values not significantly different from the WT (Figure 5a; *R*/*S* ratio = 0.47 in both lines), despite the distinct subcellular localizations of the two proteins (Figure 3a–c). These results indicate that the cytosolic ALNS‐S isoform is functionally equivalent to the peroxisomal ALNS‐L isoform in restoring allantoin metabolism in Arabidopsis.

### Both *AtALNS* splice variants rescue the early growth of *alns* mutants supplied with an allantoin precursor

Because AtALNS‐S restored allantoin metabolism as effectively as AtALNS‐L, we tested its physiological relevance to purine catabolism. We examined post‐germination growth on xanthosine, a relatively water‐soluble upstream precursor of allantoin (Figure 1), supplied as the sole nitrogen source (Figure 6). Although xanthosine delayed vegetative development, WT seedlings successfully produced true leaves under these conditions, demonstrating the functional operation of the catabolic pathway. In contrast, *alns‐1* seedlings developed expanded cotyledons but failed to progress beyond that stage and ultimately died. Expression of either *AtALNS‐L* or *AtALNS‐S* cDNA restored true leaf formation to WT levels. Similar restoration was observed in *alns‐1* lines expressing EGFP‐fused AtALNS isoforms (Figure S8). When allantoin was used as the sole nitrogen source, no significant differences in leaf development were observed among WT, *alns‐1*, and the complemented lines; all lines produced true leaves (Figure 6). As a negative control, the downstream *ALN*‐defective mutant *aln‐1* failed to grow when supplied with either xanthosine or allantoin as the sole nitrogen source. These results indicate that ALNS activity is required for growth on purine‐derived nitrogen sources and that either AtALNS isoform can restore this function.

**Figure 6:**
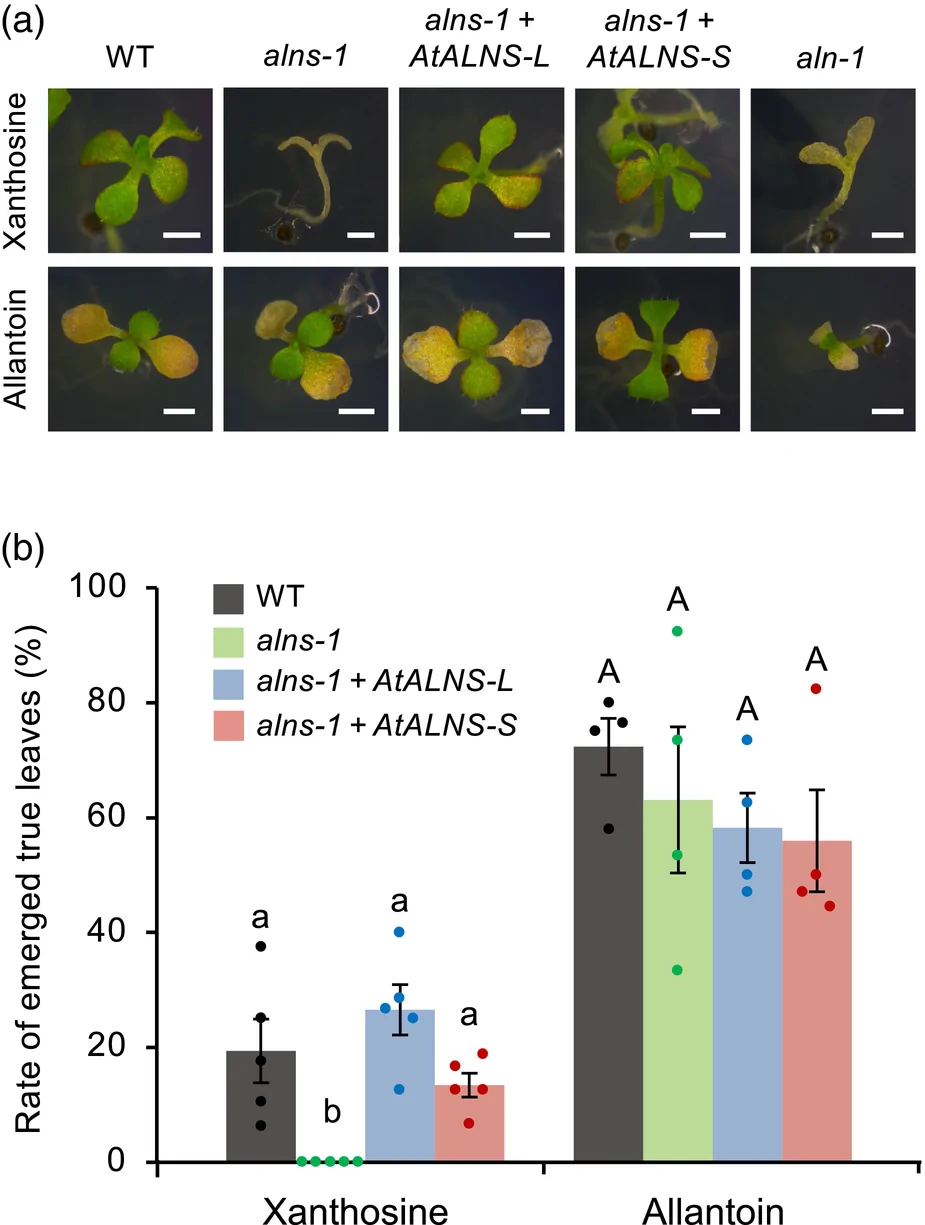
Both *AtALNS* splice variants rescue the growth defect of *alns‐1* plants supplied with purine metabolites as the sole nitrogen source. (a) Representative images of each genotype after 20 days of growth under long‐day conditions on half‐strength MS solid medium lacking nitrogen and supplemented with 5 mM xanthosine or allantoin (see Figure 1). Scale bars, 1 mm. (b) Quantification of true leaf emergence in the experiments shown in (a). True leaves were scored as emerged when both the first and second leaf blades were ≥1 mm long. Values represent means ± SEM of five (xanthosine) or four (allantoin) biological replicates (*n* ≥ 55 seedlings per genotype and condition). Different letters indicate statistically significant differences (chi‐squared test with Bonferroni correction, *P* < 0.05).

### Both *AtALNS* splice variants substantially rescue the early‐flowering phenotype of *alns* mutants

Allantoin metabolism contributes to nitrogen remobilization, and nitrogen deficiency promotes flowering in Arabidopsis (Sanagi et al., 2021). Because *alns* mutants exhibit altered allantoin metabolism (Figure 5) and appear to be impaired in purine‐derived nitrogen use (Figure 6), we examined flowering time in these mutants. Under our experimental conditions, *alns‐1* and an independent allele, *alns‐2*, flowered earlier than WT (Figure 7a; Figure S9A–D). This phenotype was substantially rescued by expressing either AtALNS‐S or AtALNS‐L or by expressing constructs encoding the corresponding EGFP fusion proteins (Figure 7; Figure S9E). Introducing the genomic *AtALNS* construct significantly delayed flowering; approximately 90% of these complemented plants remained vegetative even after 35 days, by which point most WT plants had already flowered (Figure S10). This extreme delay is likely attributable to non‐physiological overexpression, as *ALNS* transcript levels in these plants were approximately eightfold higher than in WT (Figure S4D). Together, these results indicate that both ALNS isoforms rescue the early‐flowering phenotype of *alns* mutants to a comparable extent and suggest a role for ALNS‐dependent allantoin metabolism in floral transition timing.

**Figure 7:**
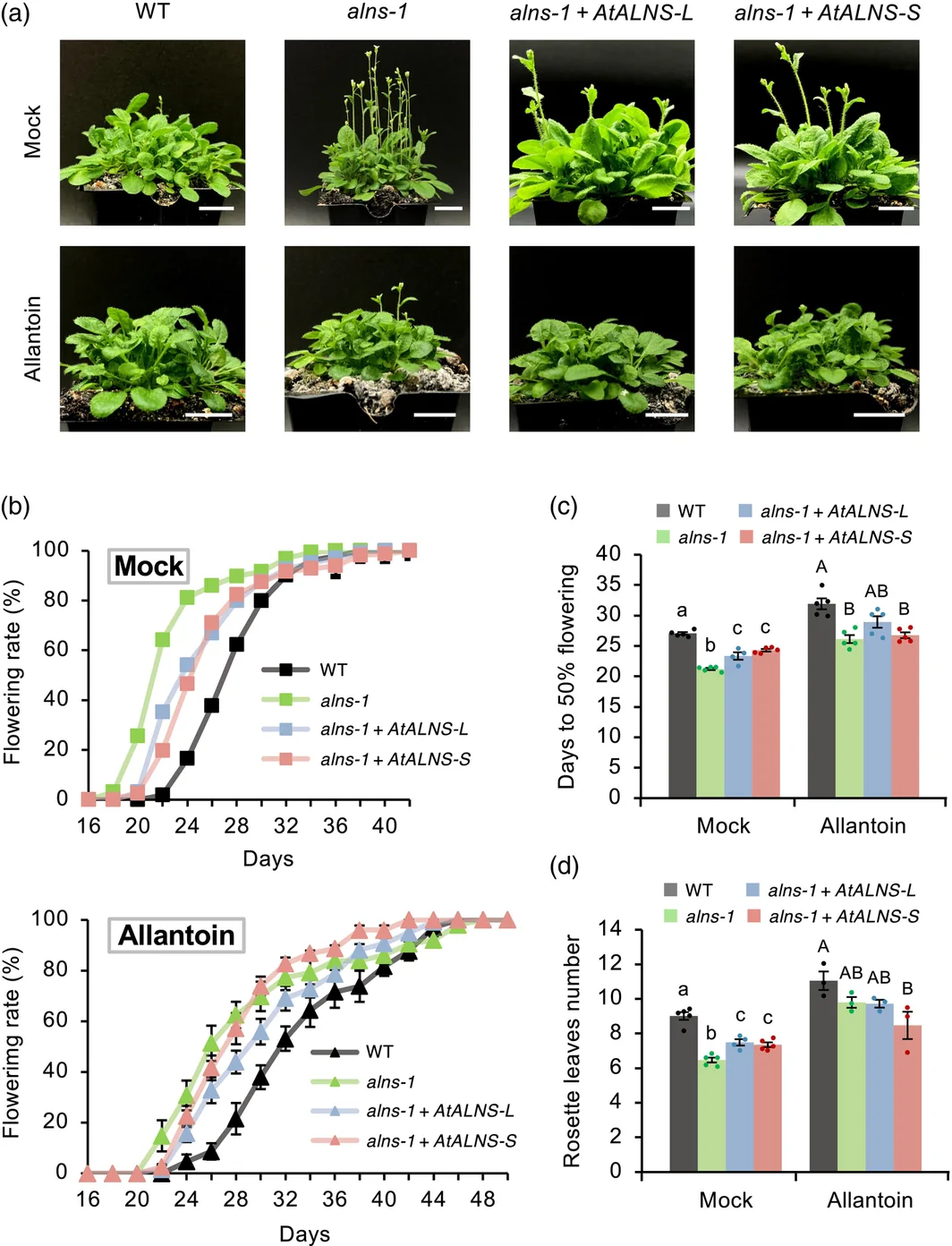
Both *AtALNS* splice variants substantially rescue the early‐flowering phenotype of the *alns‐1* mutant. (a) Representative flowering phenotypes of each genotype at 21 days under long‐day conditions. For allantoin treatment, plants were watered twice weekly, except that in one of every four waterings, tap water was replaced with 5 mM allantoin solution. Mock‐control plants were watered twice weekly with tap water. Scale bars, 1 cm. (b) Flowering rate. (c) Days to 50% flowering. (d) Number of rosette leaves at bolting, as defined in the Materials and Methods. Values represent means ± SEM of at least three biological replicates. Different letters indicate statistically significant differences (one‐way ANOVA followed by Tukey's multiple comparison test, *P* < 0.05).

Furthermore, because exogenous allantoin restored leaf development of *alns* mutants to WT levels (Figure 6), we examined whether allantoin application similarly rescues the early‐flowering phenotype of *alns‐1*. This treatment delayed flowering to a timing comparable to that observed in mock‐treated WT plants, with no significant differences among allantoin‐treated *alns‐1* and the two complementation lines (Figure 7b–d). Notably, allantoin treatment further delayed flowering in WT plants. Together, these observations support a link between allantoin and the timing of the floral transition.

### Both *AtALNS* splice variants rescue the salt‐sensitive germination phenotype of *alns* mutants

A recent genome‐wide association study in rice identified *OsTTL*, the rice homolog of Arabidopsis *ALNS*, as a determinant of seed germination under salt stress; *Osttl* mutants showed markedly reduced germination under saline conditions (Zhan et al., 2023). Consistent with this finding, *alns‐1* plants showed delayed and reduced germination in the presence of 100 mM NaCl (Figure 8). Expression of either *AtALNS‐S* or *AtALNS‐L* partially rescued germination at 48 h and fully restored germination to WT levels by 72 h, with the two isoforms showing comparable effects. These results indicate that ALNS activity is required for normal germination in Arabidopsis under saline conditions.

**Figure 8:**
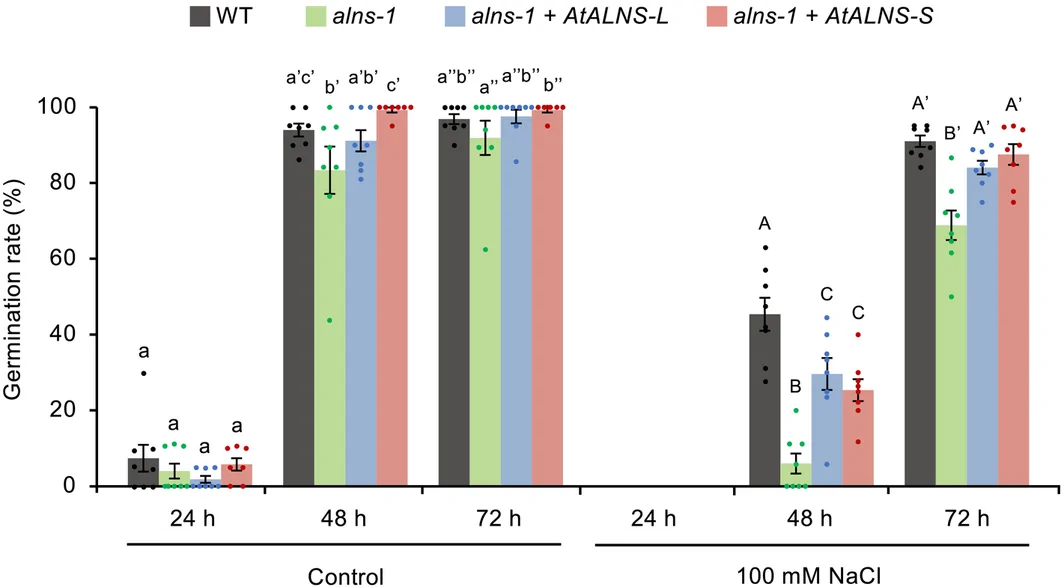
Both *AtALNS* splice variants rescue the salt‐sensitive germination phenotype of the *alns‐1* mutant. Seeds of each genotype were sown on half‐strength MS solid medium with or without 100 mM NaCl and germinated under long‐day conditions. At least 120 seeds per genotype and condition were scored, and germination frequency was monitored at the indicated time points. Because no seeds germinated at 24 h in the presence of 100 mM NaCl, all individual data points were zero and therefore not shown. Values represent means ± SEM of eight biological replicates. Different letters indicate statistically significant differences (chi‐squared test followed by Bonferroni correction, *P* < 0.05).

### Salt stress does not detectably alter the subcellular localization of AtALNS isoforms

Given that allantoin accumulation has been implicated in stress tolerance and that both AtALNS isoforms rescued the salt‐sensitive germination phenotype of *alns* mutants, we examined whether salt stress affects the subcellular localization of AtALNS‐S or AtALNS‐L. Two‐day‐old Arabidopsis seedlings stably expressing EGFP–AtALNS‐S or EGFP–AtALNS‐L were exposed to 100 mM NaCl for 24 h, and EGFP fluorescence in root tissues was compared between control and salt‐treated seedlings (Figure S11). EGFP–AtALNS‐L continued to exhibit punctate fluorescence characteristic of peroxisomes, with no detectable change in its subcellular localization. Salt stress changed the appearance of the EGFP–AtALNS‐S fluorescence pattern, although the signal remained diffusely distributed throughout the cytosol. Specifically, the large EGFP‐negative region likely corresponding to the nucleus became less evident, while multiple small EGFP‐negative regions appeared within the cells. These changes are consistent with stress‐induced reorganization of intracellular architecture, including previously reported vacuolar remodeling and nuclear shrinkage (Goswami et al., 2020; Hamaji et al., 2009). Thus, the altered fluorescence pattern of EGFP–AtALNS‐S likely reflects changes in intracellular architecture rather than protein relocalization, and salt stress did not detectably alter the subcellular localization of either AtALNS isoform under the tested conditions.

## DISCUSSION

The evolutionary diversification of ALNS, which has been regarded as a peroxisomal enzyme central to purine catabolism in land plants, illustrates how metabolic enzymes can diversify through molecular innovation. Two key events have shaped its evolution: gene fusion, generating a bifunctional enzyme, and alternative splicing, producing distinct protein isoforms. Here, we show that allantoin biosynthesis also occurs in the cytosol in Arabidopsis and that the cytosolic isoform AtALNS‐S is functionally equivalent to the peroxisomal isoform AtALNS‐L, as summarized in our updated working model (Figure 9). We also provide a mechanistic explanation for the previously reported paradoxical accumulation of allantoin in the *alns* mutants. Together, these findings expand current views on the subcellular organization of purine catabolism in plants and highlight the evolutionary and physiological significance of the diversification of ALNS isoforms.

**Figure 9:**
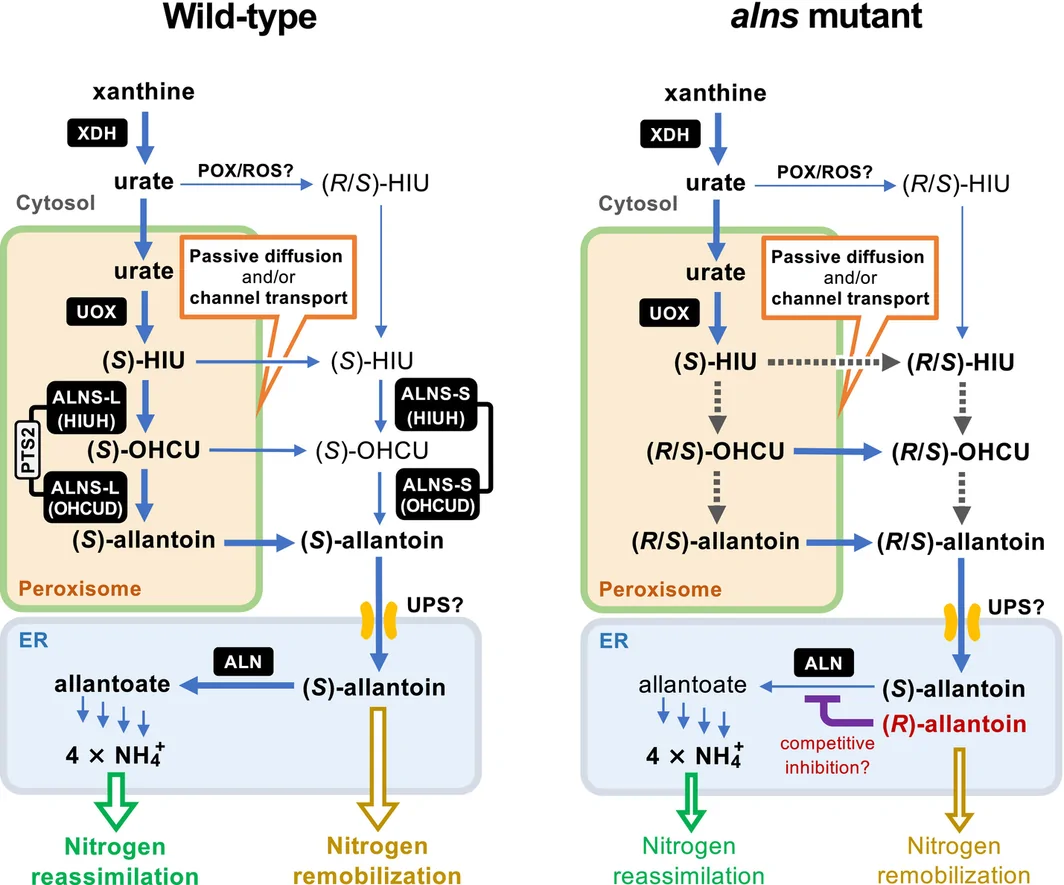
Updated working model of purine catabolism in wild‐type Arabidopsis and the metabolic consequences of ALNS loss in *alns* mutants. Wild‐type (left): In addition to the canonical peroxisomal pathway mediated by ALNS‐L, allantoin is proposed to be synthesized in the cytosol by ALNS‐S. Metabolite passage across the peroxisomal membrane may occur through passive diffusion, transport via unidentified channel‐forming proteins, or both. HIU and possibly OHCU may therefore become available in the cytosol by leakage from peroxisomes, with minor contributions from chemical or enzymatic urate oxidation associated with ROS scavenging. Cytosolic ALNS‐S may facilitate the rapid detoxification of HIU outside peroxisomes. The hypothetical involvement of the ER in allantoin export for nitrogen remobilization is based on the model proposed by Lescano et al. (2020). See Figure S12 for the corresponding models for *alns‐1* lines expressing *AtALNS* splice variants. *alns* mutants (right): In the absence of ALNS activity, HIU generated in peroxisomes or the cytosol is thought to decompose spontaneously to racemic (*R*/*S*)‐allantoin. Accumulated allantoin may be transported into the ER, where (*R*)‐allantoin could reduce ALN‐dependent turnover of (*S*)‐allantoin. Solid arrows indicate enzymatic reactions or metabolite transport, whereas dashed arrows indicate spontaneous non‐enzymatic reactions. Arrow thickness schematically represents the proposed relative magnitude of metabolic or transport fluxes. ALN, allantoinase; ER, endoplasmic reticulum; ROS, reactive oxygen species; POX, putative urate peroxidase; UPS, ureide permease.

### ALNS‐S is a non‐peroxisomal allantoin‐biosynthetic enzyme

Allantoin biosynthesis in eukaryotes, including plants, has long been regarded as a peroxisome‐confined process (Hanks & Tolbert, 1982; Ramazzina et al., 2006; Yeldandi et al., 1996). However, our comparative analysis of ALNS across the Viridiplantae, from chlorophytes to angiosperms, revealed a patchy distribution of ALNS‐S and identified several lineages of land plants and streptophyte algae that possess ALNS‐S but lack identifiable ALNS‐L homologs (Figure 2; Table S1). These findings challenge the prevailing view that allantoin biosynthesis is restricted to peroxisomes and suggest that it can also occur in the cytosol. Consistent with this interpretation, AtALNS‐S is cytosolic, and the *D. erythrosora* and *M. polymorpha* proteins are also most likely cytosolic enzymes that catalyze (*S*)‐allantoin formation *in vitro* (Figures 3 and 4). Moreover, AtALNS‐S alone complemented the *alns* mutant as effectively as AtALNS‐L (Figures 5, 6, 7, 8; Figures S5 and S8–S10). This agrees with previous *in vitro* studies showing that recombinant AtALNS‐S and AtALNS‐L have similar enzymatic activities (Lamberto et al., 2010). Therefore, AtALNS‐S functions as a cytosolic allantoin‐biosynthetic enzyme *in planta*. Before it was recognized as an ALNS isoform, ALNS‐S, originally identified as TTL, was implicated in brassinosteroid signaling in Arabidopsis (Nam & Li, 2004), and this role has recently been extended to rice (Yu et al., 2024). How these distinct functions are coordinated remains an important question.

### The subcellular localization of ALNS changed during plant evolution

No ALNS homologs were identified in the rhodophytes or glaucophytes examined in this study. By contrast, chlorophytes contained either separate HIUH and OHCUD enzymes or ALNS alone, whereas streptophyte algae and land plants possessed only ALNS (Figure 2; Table S1). These observations suggest that ALNS arose through the fusion of HIUH and OHCUD in a chlorophyte ancestor. The ancestral ALNS was likely ALNS‐L, because all chlorophyte ALNS proteins examined contained a PTS2 between the two domains. In lineages possessing only ALNS‐S, including some streptophyte algae, mosses, liverworts, and certain monilophytes, the PTS2 appears to have been lost independently from ancestral ALNS‐L. Importantly, the general retention of identifiable homologs of the upstream enzymes XDH and UOX in these lineages (Tables S2 and S3) supports the view that ALNS‐S remains integrated into the purine catabolic pathway rather than having been retained solely for an unrelated function. Although the relocalization of peroxisomal enzymes has been documented in animals (for example, Danpure, 1997), such examples remain rare in plants. The evolution of a cytosolic ALNS through the loss of PTS2 might represent a notable case of subcellular diversification with retained enzymatic function, although broader taxonomic sampling will be needed to refine this scenario.

Why PTS2 has been lost independently across multiple lineages remains unclear, but several non‐mutually exclusive explanations can be proposed. First, the primary function of ALNS might be the efficient conversion of HIU to (*S*)‐allantoin rather than its localization to a specific subcellular compartment, which is consistent with our finding that AtALNS‐S and AtALNS‐L showed indistinguishable activities in restoring allantoin levels, enantiomeric composition, and growth phenotypes in *alns* mutants. Given the permeability of the peroxisomal membrane, HIU and possibly OHCU might also become available in the cytosol (see below). Second, cytosolic allantoin production might facilitate downstream metabolism and transport, as peroxisome‐derived allantoin is thought to be released to the cytosol prior to further degradation or long‐distance transport (Lescano et al., 2020). Third, HIU‐to‐allantoin conversion might be maintained more stably in the cytosol than in peroxisomes, which undergo dynamic oxidative metabolism, protein turnover, and remodeling via pexophagy (Lingard et al., 2009; Shibata et al., 2013; Willekens et al., 1997). An additional possibility is that the loss of PTS2 might improve the structural stability of ALNS. The crystal structure of a diatom ALNS shows no direct interactions between the OHCUD and HIUH domains (Oh et al., 2018), suggesting that proteolytic cleavage between these domains by a PTS2‐processing protease such as DEG15 (Schuhmann et al., 2008) might destabilize the enzyme. Consistent with this possibility, immunodetection of EGFP–AtALNS‐L, but not EGFP–AtALNS‐S, revealed an additional band (Figures S1C and S8A, red arrowhead), possibly corresponding to a processed EGFP–OHCUD–PTS2 fragment lacking the HIUH domain. These observations raise the question of how HIU becomes available in the cytosol despite being generated in peroxisomes.

### Potential sources of HIU for cytosolic allantoin biosynthesis

The occurrence of allantoin biosynthesis in the cytosol implies that the precursors HIU and OHCU are also present in the cytosol. However, UOX, which generates HIU, is targeted to peroxisomes via its conserved PTS1 (Table S3), making the direct cytosolic production of HIU unlikely under the canonical pathway. Urate peroxidase activity has been reported in plant extracts (Tajima et al., 1985), although its localization and physiological significance remain unclear. Studies in yeast and mammals have shown that the peroxisomal membrane is permeable to small metabolites of up to 600 Da, including uric acid (168 Da), which can diffuse across the membrane (Antonenkov et al., 2004) or pass through channel‐forming proteins such as PMP22/PXMP2 (Chornyi et al., 2021; Rokka et al., 2009). Given their comparable molecular sizes, HIU, and possibly OHCU may likewise traverse the peroxisomal membrane by passive diffusion, channel‐mediated transport, or both; plant homologs of PMP22/PXMP2 represent possible candidates for the latter mechanism (Charton et al., 2019; Tugal et al., 1999). Because peroxisomes are major sites of reactive oxygen species (ROS) production, membrane integrity might be compromised during stress or senescence, allowing HIU or OHCU to escape into the cytosol.

ROS may also generate HIU in the cytosol, as cytosol‐derived urate can undergo non‐enzymatic oxidation to form HIU or related intermediates that subsequently decompose to allantoin (Ames et al., 1981; Santos et al., 1999; Wrona & Dryhurst, 1979). The occurrence of allantoin in UOX‐deficient animals, including humans and birds, supports the physiological plausibility of this pathway (Grootveld & Halliwell, 1987; Simoyi et al., 2003). In Arabidopsis, urate scavenges chloroplast‐derived ROS (Ma et al., 2016), further supporting physiologically relevant urate–ROS interactions in plant cells. Overall, leakage of HIU, and possibly OHCU, from peroxisomes appears to be the most plausible route supplying cytosolic intermediates for non‐peroxisomal allantoin biosynthesis, although enzymatic and non‐enzymatic urate oxidation in the cytosol could also contribute to this process (Figure 9).

### ALNS‐mediated regulation of HIU metabolism, allantoin homeostasis, and downstream catabolism

Once present in the cytosol, the stereochemical fate of HIU can critically affect allantoin homeostasis. Although HIU is generated enzymatically by UOX in an optically active form, it is chemically unstable and undergoes spontaneous decomposition via OHCU to racemic allantoin (Kahn et al., 1997; Kahn & Tipton, 2000). In addition, ROS‐dependent non‐enzymatic urate oxidation could also generate racemic HIU. Although this pathway is unlikely to represent a major source of cytosolic HIU under normal conditions, it might become more relevant during oxidative stress. In such cases, the ALNS‐preferred enantiomer would undergo stereospecific conversion and the other could potentially contribute to non‐enzymatic (*R*)‐allantoin formation. Consistent with this model, racemic allantoin accumulated only when both AtALNS isoforms were absent (Figure 5a), indicating that either AtALNS‐L or AtALNS‐S converts HIU stereospecifically and thereby limits racemic allantoin formation.

A further consequence may involve downstream catabolism, as accumulated (*R*)‐allantoin may interfere with the degradation of allantoin. One possibility is that (*R*)‐allantoin, a poor substrate for ALN (Lee & Roush, 1964; Vogels et al., 1966), competitively inhibits (*S*)‐allantoin turnover, thereby reducing allantoate formation. Although ALN hydrolyzes (*S*)‐allantoin to allantoate (Figure 1), structurally related compounds such as hydantoin competitively inhibit this enzyme without being metabolized (Peng & Huang, 2014). Molecular docking suggested that (*S*)‐ and (*R*)‐allantoin occupy the same predicted substrate‐binding region of AtALN, displaying broadly similar positioning relative to conserved substrate‐interacting residues but different orientations of the hydantoin moiety (Figure S7; Table S4). This model could explain the decreased allantoate levels observed in *alns* mutants despite increased *ALN* expression (Figure 5b; Figure S6), the latter likely reflecting a compensatory response to racemic allantoin accumulation. Further biochemical analyses will be needed to test this hypothesis.

Together, as illustrated in Figure 9, these findings help explain the previously reported paradoxical accumulation of allantoin in Arabidopsis *alns* mutants (Chen et al., 2020), which likely results from the spontaneous decomposition of HIU/OHCU intermediates, followed by impaired downstream utilization (see Figure S12 for models including complementation by ALNS‐L and ALNS‐S). Thus, ALNS may channel unstable intermediates into the correct stereochemical product while preventing wasteful nitrogen loss or deleterious side reactions. However, the metabolic consequences of ALNS loss may differ among plant lineages. In Arabidopsis, loss of ALNS led to spontaneous accumulation of racemic allantoin, whereas in some other species this outcome could be modified by auxiliary routes for HIU metabolism, such as a β‐glucosidase‐like protein with HIUH activity reported in soybean (*Glycine max*) nodules (Raychaudhuri & Tipton, 2002; Sarma et al., 1999).

### ALNS might function as a detoxification enzyme

Beyond its role in nitrogen metabolism, ALNS might also protect cells from the potentially cytotoxic effects of HIU. *HIUH*‐knockout mice develop hepatomegaly and tumors, and the levels of HIU‐derived protein adducts increase in human plasma during inflammation, underscoring the harmful effects of HIU accumulation (Stevenson et al., 2010; Turner et al., 2018). Notably, some bacteria, nematodes, birds, and certain hominoids have retained *HIUH* homologs despite lacking functional *UOX* genes (Hennebry et al., 2006; Keebaugh & Thomas, 2010; Ramazzina et al., 2006). These observations suggest that HIU‐detoxifying activity might be selectively retained even when canonical purine catabolism is reduced or absent. ALNS could therefore contribute to cellular detoxification by rapidly converting HIU to (*S*)‐allantoin. If so, the severe growth defect of *alns* mutants on xanthosine (Figure 6) may not solely reflect impaired utilization of purine‐derived nitrogen but also partly result from increased cellular exposure to HIU in the absence of ALNS activity.

### Implications for the relationship between allantoin accumulation and stress tolerance

Another physiological implication concerns the frequently reported association between allantoin accumulation and stress tolerance (see Kaur et al., 2021, and references therein). Both the endogenous accumulation and exogenous application of allantoin enhance salt tolerance in Arabidopsis (Irani & Todd, 2018; Lescano et al., 2016). By contrast, *alns* mutants accumulate high levels of allantoin yet exhibit salt‐sensitive germination, a phenotype also reported in rice mutants defective in the homologous gene (Zhan et al., 2023). The expression of either *ALNS* splice variant rescued this phenotype in Arabidopsis (Figure 8). These observations indicate that total allantoin abundance alone does not determine stress tolerance and is insufficient for protection in the absence of ALNS activity. Because allantoin can stimulate abscisic acid, jasmonate, and auxin biosynthesis and signaling responses (Takagi et al., 2016; Watanabe et al., 2014; Yang et al., 2018), these effects might depend on the stereochemical form of allantoin, potentially through enantiomer‐selective transport, perception, or metabolism, although this remains to be tested experimentally. Alternatively, the accumulation of labile upstream intermediates such as HIU/OHCU or the disruption of purine catabolic flux may offset any beneficial effects of elevated allantoin levels.

### Evolutionary significance of dual ALNS localization through alternative splicing

As discussed above, cytosolic localization of ALNS‐S might confer functional advantages. The ability of cytosolic AtALNS‐S to suppress racemic allantoin formation as effectively as peroxisomal AtALNS‐L suggests that HIU rapidly crosses the peroxisomal membrane and reaches the cytosol before substantial spontaneous decomposition occurs. This raises the question of why angiosperms retained ALNS‐L after acquiring ALNS‐S, rather than relying on the latter alone. Their co‐existence may reflect spatially complementary functions rather than simple redundancy: ALNS‐L metabolizes HIU at its site of production in peroxisomes, whereas ALNS‐S converts HIU that escapes into the cytosol. This dual system may become particularly important under stress conditions, when allantoin biosynthesis and accumulation are enhanced (Kaur et al., 2021) because it could facilitate the rapid removal of labile intermediates.

In eukaryotes, protein isoforms with distinct subcellular localizations generally arise through gene duplication, often resulting in multigene families (Hughes, 1994; Lynch & Conery, 2000). However, ALNS isoforms in angiosperms are produced from a single gene via alternative splicing (Lamberto et al., 2010). By selectively including or excluding a targeting signal, this post‐transcriptional mechanism enables functionally equivalent enzymes to operate in different subcellular compartments while avoiding the sequence divergence that often accompanies duplicated genes and leads to neofunctionalization, subfunctionalization, or non‐functionalization (Lynch & Conery, 2000). The acquisition of this regulatory mechanism suggests that selective pressure favored the maintenance of ALNS activity in both peroxisomes and the cytosol. Such dual localization may have conferred a selective advantage by increasing the flexibility and robustness of purine‐derived nitrogen reutilization under fluctuating environmental conditions.

In summary, our findings demonstrate that allantoin biosynthesis in plants is not restricted to peroxisomes but also operates in the cytosol through ALNS‐S. The dual localization of ALNS likely helps maintain metabolic homeostasis by limiting spontaneous side reactions of HIU while supporting efficient nitrogen remobilization. How allantoin accumulation helps protect plants from stress remains to be elucidated. More broadly, this work reveals previously unrecognized flexibility in the subcellular organization of plant purine catabolism, suggesting that even established plant metabolic pathways may conceal similar spatial and functional complexity.

## MATERIALS AND METHODS

### Plant materials and chemicals

*Arabidopsis thaliana* (L.) Heynh. accession Columbia‐0 was used as the WT, and all mutant lines were in the Columbia‐0 background. Two T‐DNA insertion lines for *ALNS* (AT5G58220), *alns‐1* (SALK_137289) and *alns‐2* (SAIL_628_A04), corresponding to *ttl‐1* and *ttl‐2*, respectively (Chen et al., 2020), were obtained from the Arabidopsis Biological Resource Center (Ohio State University, Columbus, OH, USA). Homozygosity of each T‐DNA insertion was confirmed by PCR genotyping using gene‐specific and T‐DNA border primers (Table S5). The *aln‐1* mutant for *ALN* (AT4G04955) was described previously (Watanabe et al., 2014; Yang & Han, 2004). Uric acid was obtained from Wako Pure Chemical Industries (Osaka, Japan). Racemic allantoin and allantoic acid were purchased from Nacalai Tesque, Inc. (Kyoto, Japan) and Toronto Research Chemicals (Toronto, Canada), respectively. Xanthosine dihydrate and recombinant UOX from *Candida* sp. were purchased from Sigma‐Aldrich (St. Louis, MO, USA).

### Growth conditions and phenotypic analysis

Surface‐sterilized seeds were sown on half‐strength Murashige and Skoog (MS) medium supplemented with 1% (w/v) sucrose and solidified with 0.8% (w/v) agar. The plates were incubated at 4°C for 2 days for seed stratification and transferred to a growth chamber at 23°C with a 16‐h light/8‐h dark photoperiod under white light (100 μmol photons m^−2^ s^−1^). For germination assays, seeds were sown on medium with or without 100 mM NaCl, and germination was scored for up to 72 h as the percentage of seeds showing primary root extrusion relative to the total number of seeds sown per plate. To analyze growth under different nitrogen sources, seeds were germinated and grown for 20 days on half‐strength MS medium lacking KNO_3_, NH_4_NO_3_, and sucrose (M531; PhytoTechnology Laboratories, Lenexa, KS, USA). The medium was supplemented with either 5 mM xanthosine or allantoin as the nitrogen source and with 20 mM KCl to maintain potassium levels. The lengths of the first and second true leaf blades were measured using ImageJ 1.53e, and the percentage of individuals with both leaf blades ≥1 mm long was determined. To analyze flowering time, plants were grown in commercial potting soil (Hanasaki Monogatari; Akimoto Tensanbutsu, Iga, Japan) containing 290 mg of nitrogen, 620 mg of phosphorus, 285 mg of potassium, and trace amounts of magnesium, boron, iron, and manganese per liter of dry soil. The plants were watered twice weekly with tap water. For allantoin supplementation, plants received the same watering regime, except that once every 2 weeks, the plants were watered with 5 mM allantoin solution rather than tap water. Flowering time was defined as the number of days until 50% of the plants had produced a primary inflorescence at least 25 mm long (“days to 50% flowering”) and as the total number of rosette leaves at that stage. Flowering time for the experiment shown in Figure S9 was evaluated as described in Methods S4. All growth periods are given as days after germination.

### Sequence retrieval, multiple sequence alignment, and subcellular localization prediction

ALNS, HIUH, and OHCUD sequences from diverse plant species were retrieved using BLASTP and tBLASTn searches against NCBI databases, with previously reported AtALNS sequences used as queries (Lamberto et al., 2010; Ramazzina et al., 2006). Searches were performed using the default settings of the respective BLAST search tools against the NCBI ClusteredNR, Expressed Sequence Tag, and Transcriptome Shotgun Assembly databases, as well as the China National GeneBank database and Phytozome v13 (Goodstein et al., 2012). Previously reported ALNS sequences were also used for sequence alignment (Kechasov et al., 2020; Lamberto et al., 2010; Oh et al., 2018; Reumann et al., 2007). XDH and UOX sequences were retrieved using the same procedure, with the corresponding Arabidopsis sequences used as queries (Hesberg et al., 2004; Marchfelder et al., 1997).

The retrieved sequences of ALNS/HIUH/OHCUD, XDH, and UOX are listed in Tables S1–S3, respectively. Putative PTS1 motifs were predicted using PTS1 Predictor (Neuberger et al., 2003), whereas PTS2 motifs were identified by manual inspection based on the consensus sequence RΦXXΦXX(H/Q)Φ, where Φ represents a hydrophobic residue and X denotes any residue (Kunze, 2020). Subcellular localization of XDH and UOX was predicted using DeepLoc 2.1 (Thumuluri et al., 2022).

### DNA constructs and plant transformation

To construct native promoter‐driven expression vectors for cDNA complementation, the *AtALNS* promoter (ALNSpro) was amplified from WT genomic DNA by PCR. The coding sequences for AtALNS with or without the PTS2 motif were amplified from the full‐length cDNA clones pda03063 and pda17682, respectively, from the RIKEN BioResource Research Center (Tsukuba, Japan). The promoter and each cDNA fragment were assembled together into the pENTR vector (Thermo Fisher Scientific, San Jose, CA, USA) using an In‐Fusion HD Cloning Kit (Takara Bio, Kyoto, Japan). Following sequence verification of the resulting entry clones, each promoter:cDNA fusion sequence was transferred into the binary vector pGWB1 (Nakagawa et al., 2007b) using Gateway LR Clonase II Enzyme Mix (Thermo Fisher Scientific), yielding the constructs *ALNSpro:AtALNS‐L* and *ALNSpro:AtALNS‐S*. For genomic complementation, the *AtALNS* genomic sequence, including its promoter and entire gene, was PCR‐amplified, cloned into pENTR, and transferred into pGWB1 as described above, generating ALNSpro:gAtALNS.

To generate N‐terminal fusions with EGFP under the control of the cauliflower mosaic virus *35S* promoter, the above entry clones were modified by inverse PCR to remove the *AtALNS* promoter and recircularized by self‐ligation. The verified entry clones were then transferred into the pGWB6 binary vector (Nakagawa et al., 2007b), generating the constructs *35S:EGFP*–*AtALNS‐L*, *35S:EGFP*–*AtALNS‐S*, and *35S:EGFP*–g*AtALNS*. For localization of a gymnosperm ALNS, the coding sequence for TpALNS was custom‐synthesized based on the cDNA sequence (Phytozome ID: Thupl.29379470s0024.1) and cloned into the pDONR221 vector by the GeneArt service (Thermo Fisher Scientific). This synthetic cDNA was subsequently transferred into the expression vector pGWB555 to construct an N‐terminal fusion with mRFP under the control of the *35S* promoter (Nakagawa et al., 2007b), yielding 35S:mRFP–TpALNS. Similarly, mRFP‐fused constructs for monilophyte and bryophyte ALNS proteins were generated using synthesized coding sequences for DeALNS (NCBI accession no. GHEW01015885.1) and MpALNS (MarpolBase ID: Mp4g21780.1; Montgomery et al., 2020), resulting in the constructs 35S:mRFP–DeALNS and 35S:mRFP–MpALNS.

The PCR primer sequences are listed in Table S5. All constructs were introduced into *Agrobacterium tumefaciens* strain GV3101(pMP90) and used for stable transformation of Arabidopsis by the floral dip method or for transient transformation of Arabidopsis.

### Agroinfiltration‐mediated transient expression

Transient expression in Arabidopsis cotyledons and leaves was performed using *A. tumefaciens* strain GV3101(pMP90) as described by Zhang et al. (2020), with minor modifications. Glycerol stocks of *A. tumefaciens* strains carrying expression constructs were inoculated into 5 mL of LB medium and cultured at 28°C for 24 h. The bacterial cultures were centrifuged at 2000 × ***g*** for 2 min, and the cells were resuspended in infiltration buffer to an OD_600_ of 0.5. Arabidopsis cotyledons or leaves were infiltrated with the bacterial suspension. After infiltration, the plants were incubated in a greenhouse for 2–3 days. Fluorescent protein markers for peroxisome visualization included mCherry–PTS1 (pCMU‐PERr; Ivanov & Harrison, 2014) and Citrine–PTS1 (pMpGWB102‐Citrine‐PTS1; Ogasawara et al., 2013), both from Addgene (Cambridge, MA, USA; plasmids #61202 and #44592, respectively).

### Confocal laser‐scanning fluorescence microscopy

Images were acquired under a confocal laser‐scanning fluorescence microscope (Fluoview FV3000; Olympus, Tokyo, Japan). Fluorescent proteins were detected using the following excitation/emission settings: EGFP and Citrine, 488 nm/500–555 nm; mRFP and mCherry, 561 nm/615–650 nm. FM4‐64 fluorescence was observed using the same settings as for mRFP and mCherry after treating Arabidopsis roots with 2 μM FM4‐64 for 5 min at 25°C.

### Salt treatment and fluorescence microscopy

Two‐day‐old Arabidopsis seedlings expressing EGFP–AtALNS‐S or EGFP–AtALNS‐L were transferred to half‐strength MS solid medium containing 100 mM NaCl for 24 h under long‐day conditions. Root EGFP fluorescence was observed using the same confocal microscopy settings described above. Control seedlings were transferred to the same medium without added NaCl and observed under identical imaging conditions.

### Recombinant protein production and spectrophotometric enzyme assays

Coding sequences for AtALNS‐S, MpALNS, and DeALNS were custom‐synthesized and cloned into the pGA‐ET28(+) expression vector by GeneArt to encode N‐terminal hexahistidine‐tagged proteins. These plasmids were introduced into *Escherichia coli* BL21 Star (DE3), and recombinant protein production was induced with 0.1 mM isopropyl β‐D‐thiogalactopyranoside at 20°C for 20 h in LB medium supplemented with 50 μg mL^−1^ kanamycin. The hexahistidine‐tagged proteins were purified by nickel‐affinity column chromatography, desalted, concentrated, and quantified as described previously (Sakamoto et al., 2003). Purified proteins were analyzed by SDS–PAGE using a 10% polyacrylamide gel.

Enzymatic activity assays were performed at 25°C in 50 mM potassium phosphate buffer (pH 7.8), essentially as described by Pessoa et al. (2010). The effect of the recombinant proteins on UOX‐catalyzed urate oxidation was examined by monitoring the decrease in absorbance at 292 nm. Reactions were initiated by adding 50 μM urate to a reaction mixture containing 0.5 units mL^−1^ UOX and 0.5 μg mL^−1^ recombinant protein. HIUH activity was examined by monitoring the decrease in absorbance at 312 nm. HIU was first generated from 50 μM urate using 0.5 units mL^−1^ UOX, and 0.5 μg mL^−1^ recombinant protein was added after HIU accumulation had reached a plateau.

### Time‐resolved CD analysis of ALNS‐catalyzed reactions

The conversion of UOX‐generated HIU to (*S*)‐allantoin by recombinant ALNS proteins was monitored by time‐resolved CD spectroscopy using a JASCO J‐1500 spectropolarimeter (JASCO Corporation, Hachioji, Japan). Reactions were performed at 25°C in 50 mM potassium phosphate buffer (pH 7.8) containing 100 μM urate, 0.025 units mL^−1^ UOX, and 0.5 μg mL^−1^ recombinant protein. CD spectra were recorded from 200 to 350 nm at the indicated time points after initiation of the reaction in a cell with a 10‐mm path length at a scanning speed of 500 nm min^−1^. HIU and OHCU were assigned based on their characteristic negative CD peaks at approximately 312 and 257 nm, respectively, and the reaction product was assigned as (*S*)‐allantoin by comparison with the reference CD spectrum of chromatographically separated (*S*)‐allantoin described below.

### Protein extraction, immunoprecipitation, and immunoblotting

Protein extraction, immunoprecipitation, and immunoblotting were performed as described in Methods S1.

### Gene expression analyses

Conventional and quantitative reverse‐transcription PCR were performed as described in Methods S2.

### Quantification of allantoin and allantoate

Fresh tissues were homogenized in 6.7 volumes (w/v) of Milli‐Q water and centrifuged at 20000 × ***g*** for 5 min at 4°C. The supernatant was filtered through a 0.2 μm PTFE membrane (Waters, Milford, MA, USA). Allantoin and allantoate were separated by HPLC and detected at 191 nm using an LC‐20 Prominence HPLC system (Shimadzu, Kyoto, Japan) equipped with an Aminex HPX‐87H column (300 mm × 7.8 mm i.d.; Bio‐Rad, Hercules, CA, USA), essentially as described by Nourimand and Todd (2016), except that the H_2_SO_4_ concentration in the mobile phase was modified. The mobile phase contained 14 mM H_2_SO_4_ for allantoin analysis and 2.5 mM H_2_SO_4_ for allantoate analysis. Separations were performed at a flow rate of 0.5 mL min^−1^ for 60 min at 25°C.

### Chromatographic enantiomer separation and CD‐based configurational analysis of allantoin

Fresh tissues were homogenized in 10 volumes (w/v) of Milli‐Q water and centrifuged at 20000 × ***g*** for 5 min at 4°C. The supernatant was freeze‐dried, and the dried material was resuspended in 300 μL of Milli‐Q water and centrifuged again under the same conditions. The supernatant was partitioned with hexane at an aqueous phase‐to‐hexane ratio of 3:1 (v/v) to remove lipids and chlorophyll. The aqueous phase was ultrafiltered using an Amicon Ultra centrifugal filter unit (10 kDa; Millipore, Burlington, MA, USA), and the filtrate was fractionated by HPLC as described above. Fractions containing allantoin were collected, residual H_2_SO_4_ was removed using an ion‐exchange column (Extract‐Clean SPE IC‐Ba 1.5 mL/4 mL; Systech Co., Tokyo, Japan), and the eluate was freeze‐dried.

The dried material was dissolved in ethanol. Enantiomeric separation and quantification of (*S*)‐ and (*R*)‐allantoin were performed using a Shimadzu LC‐20 Prominence system equipped with a CHIRAL ART Cellulose‐SB column (250 mm × 4.6 mm i.d., 5 μm; YMC Co., Kyoto, Japan). The mobile phase consisted of hexane/ethanol (8:2, v/v), and separation was performed at a flow rate of 1.0 mL min^−1^ for 30 min at 25°C. Elution was monitored at 210 nm. Racemic allantoin was used as a reference for determining the retention times of the two enantiomers.

To assign their absolute configurations, the two enantiomeric fractions separated from the racemic allantoin standard were individually evaporated to dryness, washed with a small volume of methanol, redissolved in 100 mM potassium phosphate buffer (pH 7.0), and analyzed using a JASCO J‐720w spectropolarimeter (JASCO Corporation). CD spectra were recorded from 190 to 300 nm in a 10‐mm‐path‐length cell at a scanning speed of 100 nm min^−1^. The fractions showing positive and negative ellipticity were assigned as (*S*)‐ and (*R*)‐allantoin, respectively, according to previous reports ('s‐Gravenmade et al., 1969; Ramazzina et al., 2006).

### Molecular docking analysis

Molecular docking analysis was performed as described in Methods S3.

### Statistical analysis

Data are presented as means ± standard error of the mean (SEM) of three independent biological replicates unless otherwise noted. Statistical analyses were performed using Prism (v10.4.2; GraphPad Software, San Diego, CA, USA) or Microsoft Excel v2505 (Microsoft Corp., Redmond, WA, USA), as appropriate. Categorical variables were analyzed using the chi‐squared test with Bonferroni correction. Numerical data were analyzed using Student's *t*‐test or one‐way analysis of variance (ANOVA) followed by Tukey's or Dunnett's multiple comparison test. Differences were considered statistically significant at *P* < 0.05.

## AUTHOR CONTRIBUTIONS

YT and AS conceived and designed the study. YT performed most of the experiments. TS contributed to the subcellular localization analyses. AS performed experiments involving recombinant proteins and molecular docking analyses. YT, TS, HS, and AS analyzed the data. YT and AS wrote the manuscript. AS supervised the study. All authors read and approved the final manuscript.

## CONFLICT OF INTEREST

The authors declare no conflicts of interest.

## Supporting information


**Figure S1.** DNA constructs for EGFP‐ or mRFP‐fused ALNS used for subcellular localization and protein expression in stable Arabidopsis transformants.
**Figure S2.** Separation and identification of (*S*)‐ and (*R*)‐allantoin enantiomers.
**Figure S3.** Alternatively spliced variants of *AtALNS* mRNA and their relative expression levels across Arabidopsis organs and tissues.
**Figure S4.** Characterization of the *alns‐1* mutant and its complementation lines carrying *AtALNS* splice‐variant constructs and a genomic construct.
**Figure S5.** Enantiomeric composition of allantoin in wild‐type, *alns‐1*, and *alns‐1* lines expressing *AtALNS* splice‐variant constructs.
**Figure S6.** Relative expression levels of genes encoding purine catabolic enzymes involved in allantoin biosynthesis and degradation in wild‐type and *alns‐1* lines.
**Figure S7.** Predicted binding modes of (*S*)‐ and (*R*)‐allantoin in the putative substrate‐binding region of AtALN.
**Figure S8.** Both EGFP–AtALNS‐L and EGFP–AtALNS‐S rescue the defective growth of *alns‐1* supplied with xanthosine as the sole nitrogen source.
**Figure S9.** Characterization of the *alns‐2* mutant and rescue of the *alns‐1* early‐flowering phenotype by EGFP–AtALNS‐L and EGFP–AtALNS‐S.
**Figure S10.** Rescue of the *alns‐1* early‐flowering phenotype by *AtALNS* splice‐variant constructs and a genomic construct.
**Figure S11.** Subcellular localization of EGFP–AtALNS‐L and EGFP–AtALNS‐S under salt stress conditions.
**Figure S12.** Working models of purine metabolism in *alns‐1* mutants expressing *AtALNS* splice‐variant constructs.
**Table S1.** Domain architecture and predicted peroxisomal targeting signals (PTSs) of allantoin‐biosynthetic enzymes (ALNS, HIUH, and OHCUD) in Archaeplastida.
**Table S2.** Occurrence and predicted subcellular localization of xanthine dehydrogenases (XDHs) in Archaeplastida.
**Table S3.** Occurrence, PTS motifs, and predicted subcellular localization of urate oxidases (UOXs) in Archaeplastida.
**Table S4.** Molecular docking of (*S*)‐ and (*R*)‐allantoin to the putative substrate‐binding region of Arabidopsis allantoinase (AtALN).
**Table S5.** PCR primers used in this study.
**Methods S1.** Protein extraction, immunoprecipitation, and immunoblotting.
**Methods S2.** Conventional and quantitative reverse‐transcription PCR.
**Methods S3.** Molecular docking analysis.
**Methods S4.** Flowering time measurements.

## Data Availability

The data supporting the findings of this study are available within the article and its Supporting Information.
