# Supplementary material for: Non‐peroxisomal allantoin biosynthesis reveals unexpected flexibility in plant purine catabolism
Source: Plant J. 2026 Oct 23;128(2):e71178. doi: 10.1111/tpj.71178 (PMC13691922; doi:10.1111/tpj.71178)
Supplement: Supplementary file 1 [file TPJ-128-0-s001.pdf]

Supporting Information

Non-peroxisomal allantoin biosynthesis reveals unexpected flexibility in plant purine catabolism

Yuta Takeuchi, Tamio Sumida, Hiroshi Shimada, and Atsushi Sakamoto*

*Corresponding author: E-mail: ahkkao@hiroshima-u.ac.jp; Fax: +81(0)82-424-7449

The following supporting materials are available for this article. Supporting references are listed at the end of this document:

Supporting Figures

Figure S1. DNA constructs for EGFP- or mRFP-fused ALNS used for subcellular localization and protein expression in stable Arabidopsis transformants.

Figure S2. Separation and identification of (*S*)- and (*R*)-allantoin enantiomers.

Figure S3. Alternatively spliced variants of *AtALNS* mRNA and their relative expression levels across Arabidopsis organs and tissues.

Figure S4. Characterization of the *alns-1* mutant and its complementation lines carrying *AtALNS* splice-variant constructs and a genomic construct.

Figure S5. Enantiomeric composition of allantoin in wild-type, *alns-1*, and *alns-1* lines expressing *AtALNS* splice-variant constructs.

Figure S6. Relative expression levels of genes encoding purine catabolic enzymes involved in allantoin biosynthesis and degradation in wild-type and *alns-1* lines.

Figure S7. Predicted binding modes of (*S*)- and (*R*)-allantoin in the putative substrate-binding region of AtALN.

Figure S8. Both EGFP–AtALNS-L and EGFP–AtALNS-S rescue the defective growth of *alns-1* supplied with xanthosine as the sole nitrogen source.

Figure S9. Characterization of the *alns-2* mutant and rescue of the *alns-1* early-flowering phenotype by EGFP–AtALNS-L and EGFP–AtALNS-S.

Figure S10. Rescue of the *alns-1* early-flowering phenotype by *AtALNS* splice-variant constructs and a genomic construct.

Figure S11. Subcellular localization of EGFP–AtALNS-L and EGFP–AtALNS-S under salt stress conditions.

Figure S12. Working models of purine metabolism in *alns-1* mutants expressing *AtALNS* splice-variant constructs.

Supporting Tables

Table S1. Domain architecture and predicted peroxisomal targeting signals (PTSs) of allantoin biosynthetic enzymes (ALNS, HIUH, and OHCUD) in Archaeplastida.

Table S2. Occurrence and predicted subcellular localization of xanthine dehydrogenases (XDHs) in Archaeplastida.

Table S3. Occurrence, PTS motifs, and predicted subcellular localization of urate oxidases (UOXs) in Archaeplastida.

Table S4. Molecular docking of (*S*)- and (*R*)-allantoin to the putative substrate-binding region of Arabidopsis allantoinase (AtALN).

Table S5. PCR primers used in this study.

Supporting Methods

Methods S1. Protein extraction, immunoprecipitation, and immunoblotting.
Methods S2. Conventional and quantitative reverse-transcription PCR.
Methods S3. Molecular docking analysis.
Methods S4. Flowering time measurements.

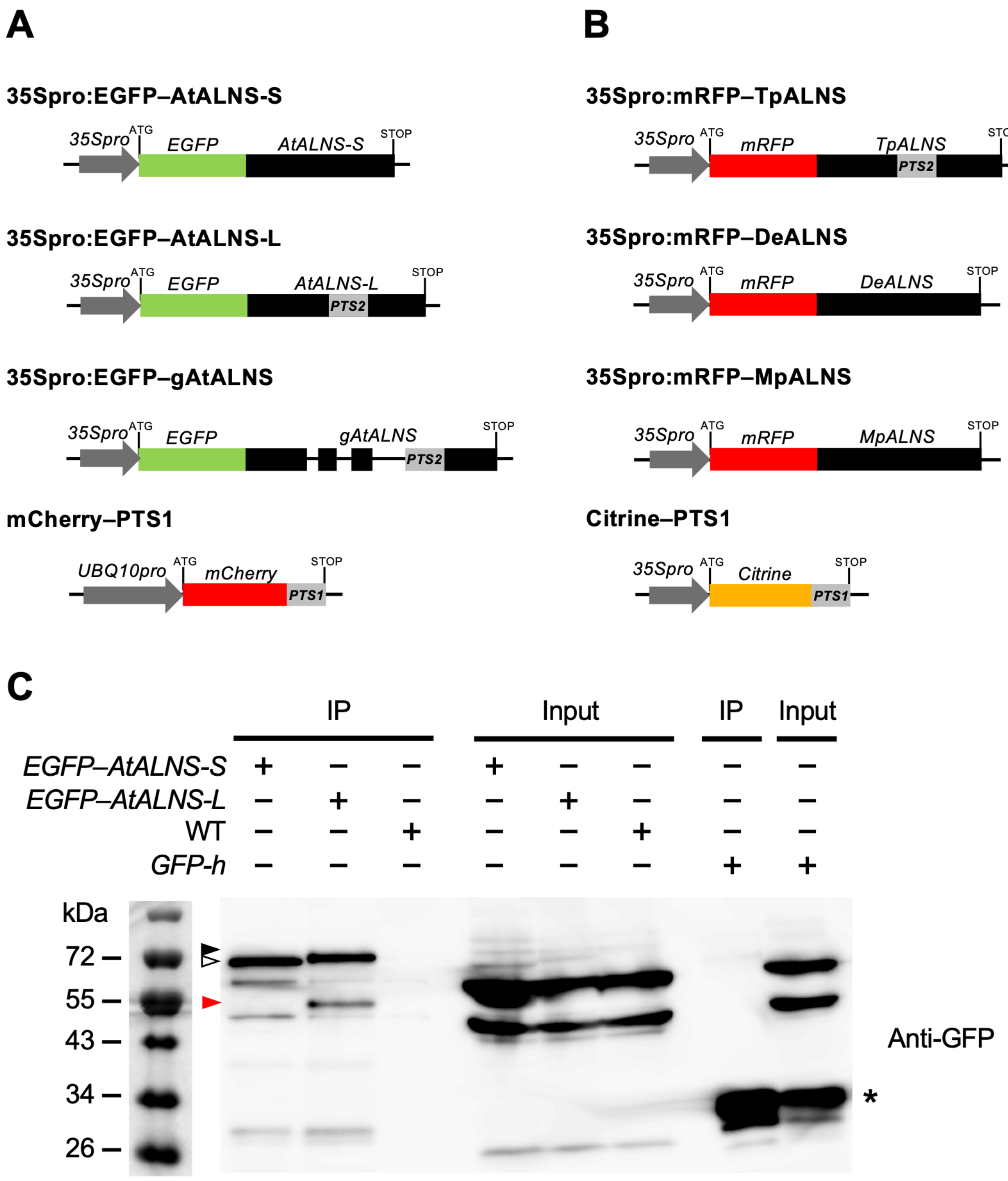

Figure S1. DNA constructs for EGFP- or mRFP-fused ALNS used for subcellular localization and protein expression in stable Arabidopsis transformants.

A. Three EGFP fusion constructs expressing Arabidopsis *AtALNS-S* and *AtALNS-L* cDNAs, and genomic *AtALNS* (*gAtALNS*), under the control of the cauliflower mosaic virus *35S* promoter in the binary vector pGWB6 (Nakagawa *et al*., 2007). mCherry fused to PTS1 under the control of the *UBQ10* promoter was used as a red fluorescent peroxisomal marker (Ivanov and Harrison, 2014).

B. Three mRFP fusion constructs expressing *ALNS* cDNAs from a gymnosperm (*Thuja plicata*, TpALNS), a monilophyte (*Dryopteris erythrosora*, DeALNS), and a bryophyte (*Marchantia polymorpha*, MpALNS), under the control of the *35S* promoter in the binary vector pGWB555 (Nakagawa *et al*., 2007). Citrine fused to PTS1 under the control of the *35S* promoter was used as a yellow fluorescent peroxisomal marker (Ogasawara *et al*., 2013).

C. Expression of EGFP–AtALNS-S and EGFP–AtALNS-L fusion proteins in transgenic Arabidopsis plants in the wild-type (WT) background. Immunoprecipitation (IP) was performed using 1 mg of total proteins extracted from 6-day-old seedlings with agarose beads conjugated to anti-GFP antibodies. Immunoblot analysis of the input and IP fractions was performed using anti-GFP antibodies. A *GFP-h* transgenic line stably expressing GFP with an endoplasmic reticulum retention signal was used as a positive control for GFP detection (Hayashi *et al*., 2001; Han *et al*., 2020). Forty micrograms of total proteins were loaded for the input samples, except for *GFP-h* (20 μg). Open and closed arrowheads indicate EGFP–AtALNS-S and EGFP–AtALNS-L, respectively. Red arrowhead denotes a band observed specifically in the EGFP–AtALNS-L sample, possibly corresponding to a processed EGFP–OHCUD–PTS2 fragment lacking the HIUH domain. The asterisk (*) indicates GFP-h.

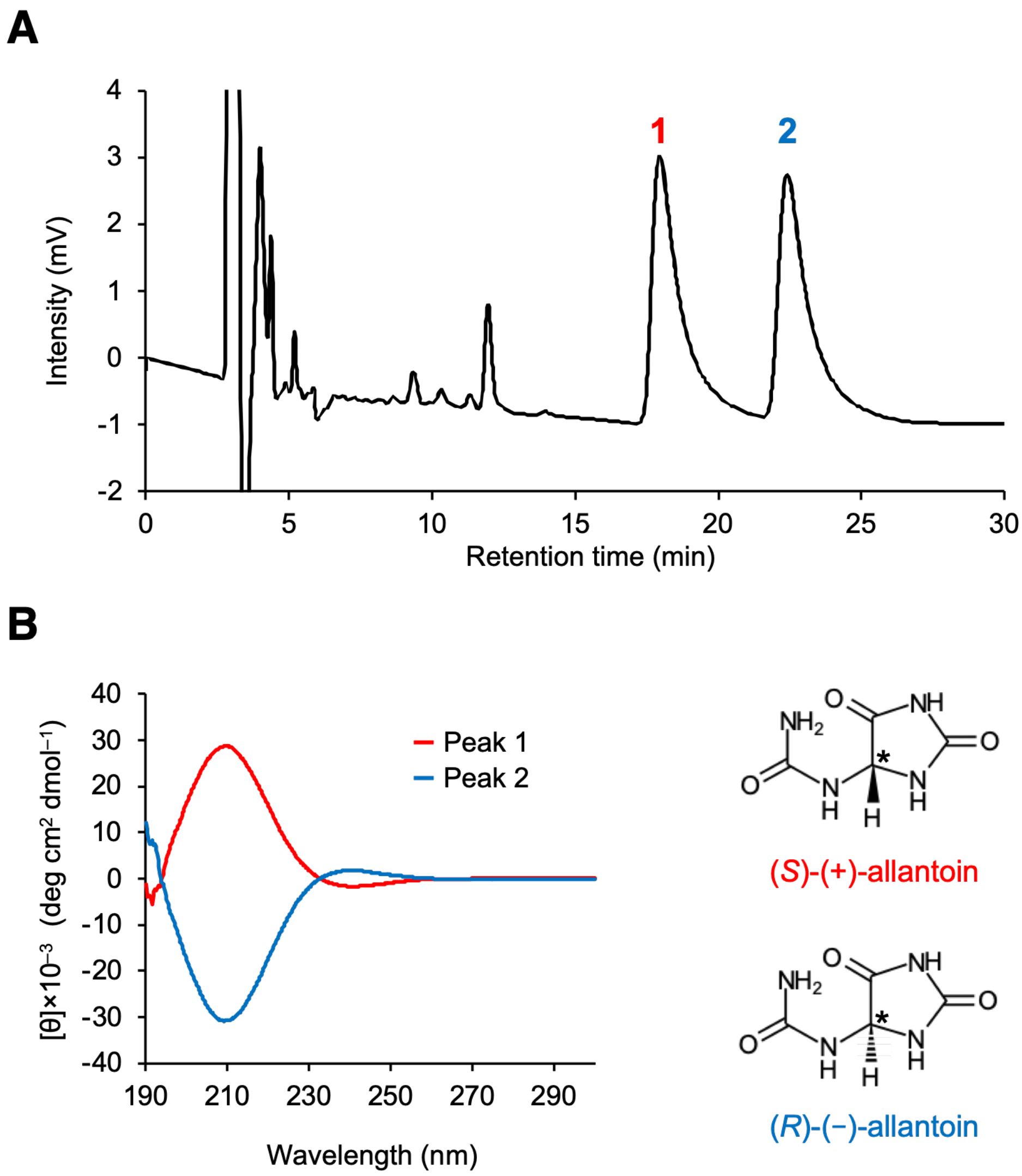

Figure S2. Separation and identification of (*S*)- and (*R*)-allantoin enantiomers.

A. Representative chiral HPLC chromatogram showing the separation of racemic allantoin into two peaks (1 and 2). A racemic allantoin standard was used to assign retention times. Experimental details are described in the main text.

B. Circular dichroism spectra of the collected fractions, expressed as molar ellipticity (θ), together with the chemical structures of (*S*)- and (*R*)-allantoin. Peak 1 exhibited positive ellipticity, whereas peak 2 exhibited negative ellipticity. Positive and negative ellipticity were assigned to (*S*)- and (*R*)-allantoin, respectively, based on previous reports ('s-Gravenmade *et al*., 1969; Ramazzina *et al*., 2006). Asterisks (*) indicate chiral centers.

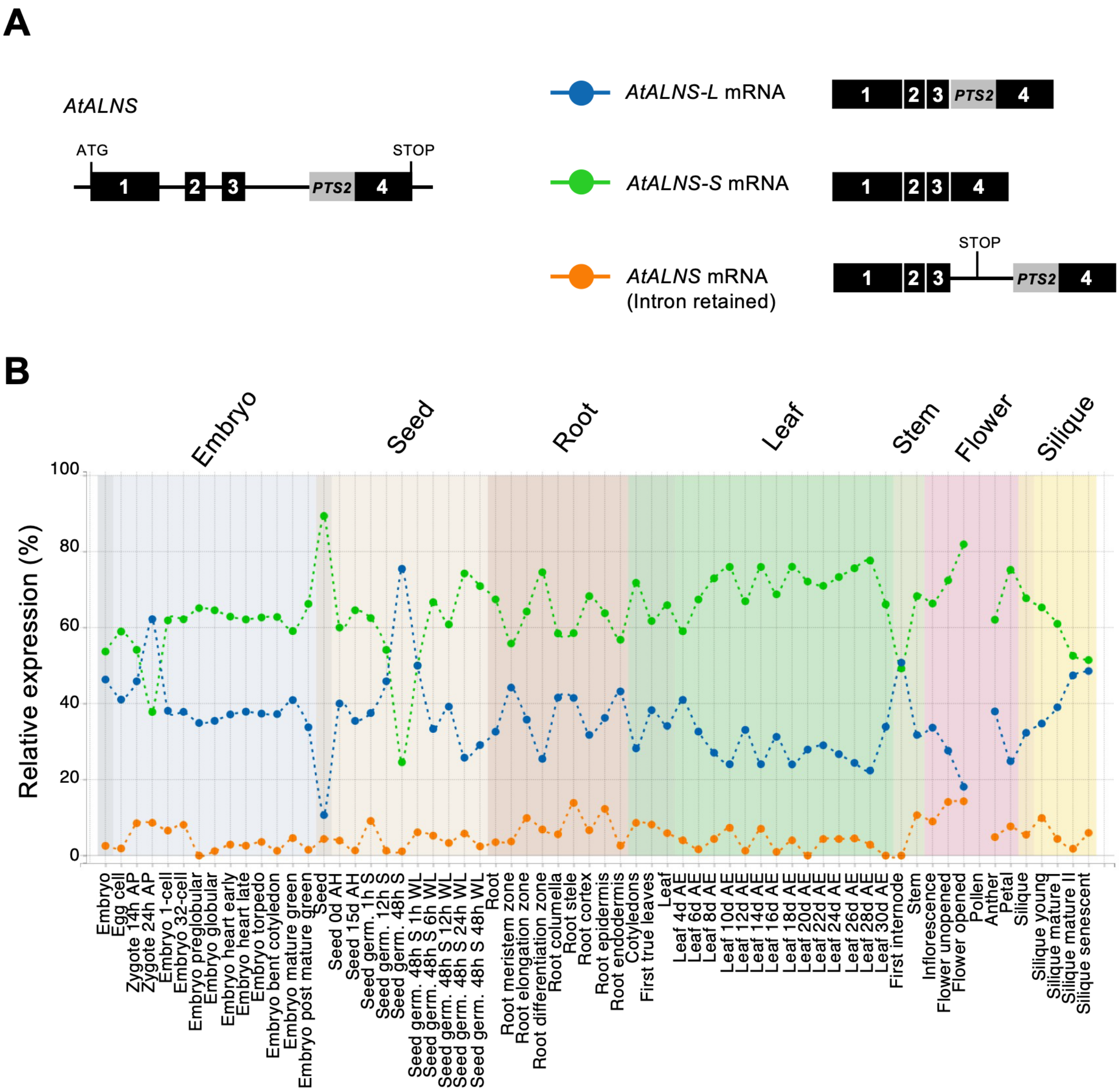

Figure S3. Alternatively spliced variants of *AtALNS* mRNA and their relative expression levels across Arabidopsis organs and tissues.

A. Genomic structure of *AtALNS* (left) and its three splice variants, *AtALNS-L*, *AtALNS-S*, and an intron-retained transcript (right), as shown in PastDB (Martín *et al*., 2021; https://pastdb.crg.eu/wiki/Main_Page). Black boxes indicate exons, numbered sequentially. Alternative splicing at an internal splice site within exon 4 removes its 5′ portion (gray box), generating *AtALNS-S* transcripts encoding an enzyme lacking the type 2 peroxisomal targeting signal (PTS2).

B. Relative transcript abundance across organs and tissues. Data were retrieved from PastDB. Pollen data are not shown because read counts were deemed insufficient in PastDB to estimate relative transcript abundance reliably. Abbreviations: AP, after pollination; AH, after harvest; S, stratification; WL, white light; AE, after emergence.

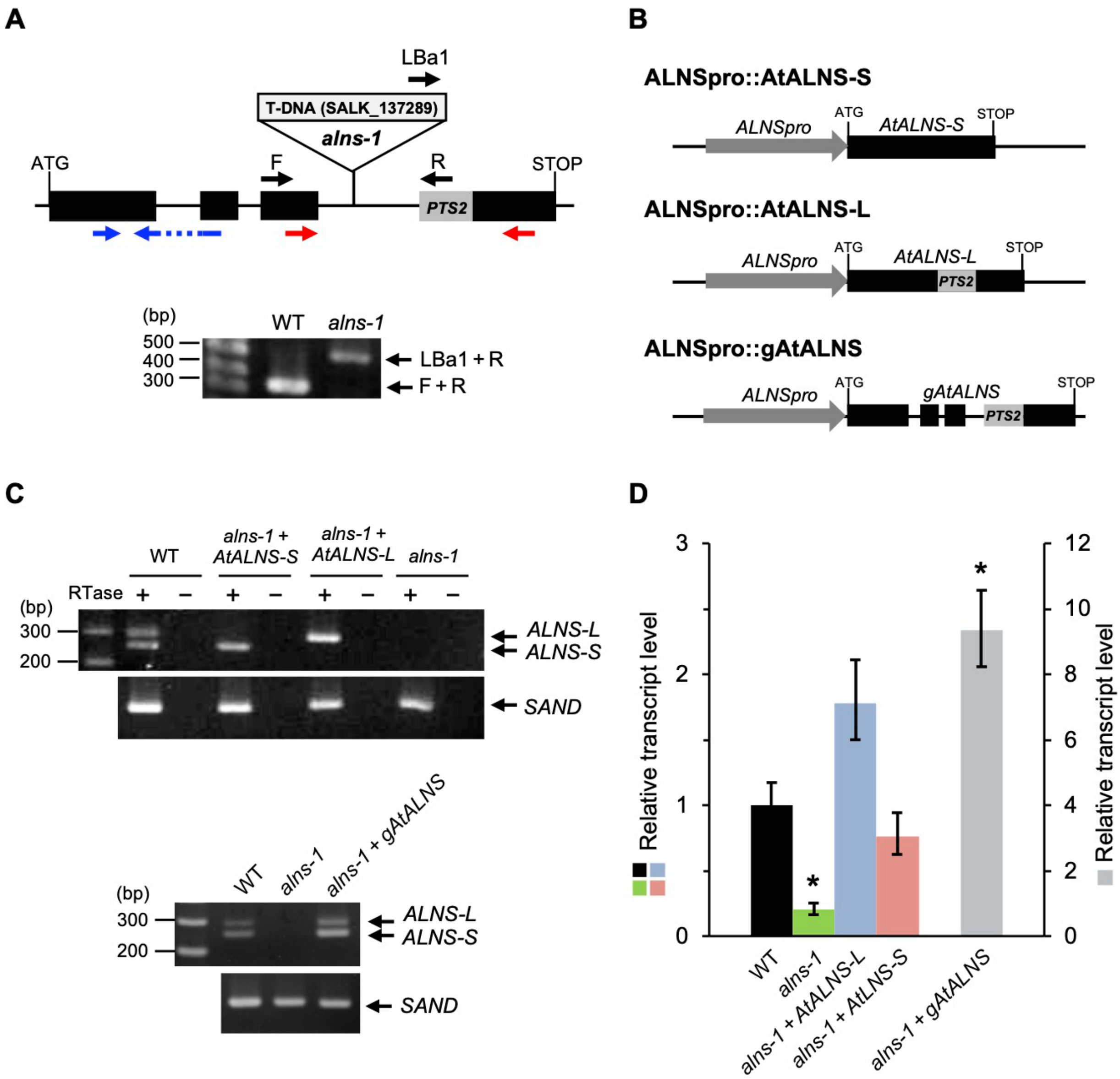

Figure S4. Characterization of the *alns-1* mutant and its complementation lines carrying *AtALNS* splice-variant constructs and a genomic construct.

- **A.** Schematic representation of the *AtALNS* gene structure showing the position of the T-DNA insertion and the corresponding SALK line identifier (upper panel), and PCR genotyping (lower panel). Arrows indicate primers used for genotyping and expression analysis: black, PCR genotyping; red, conventional reverse-transcription PCR (RT-PCR); blue, quantitative RT-PCR. Primer sequences are listed in Table S5. The expected sizes of the amplified products are 333 bp for wild-type (WT) and 442 bp for *alns-1*.
- **B.** Three complementation cassettes for stable expression of *AtALNS-S* and *AtALNS-L* cDNAs, and genomic *AtALNS* (*gAtALNS*), under the control of the native promoter (*ALNSpro*). Each cassette was cloned into the T-DNA region of the binary vector pGWB1 (Nakagawa *et al*., 2007) and introduced into the *alns-1* mutant.
- **C.** Conventional RT-PCR of *AtALNS-S* and *AtALNS-L* in WT, *alns-1*, and the three complementation lines, with *SAND* used as an internal control. Total RNA was extracted from 14-day-old seedlings grown under the standard conditions described in Materials and Methods in the main text. The expected sizes of the amplified products are 259 bp for *ALNS-S*, 298 bp for *ALNS-L*, and 244 bp for *SAND*. RTase, reverse transcriptase.
- **D.** Quantitative RT-PCR showing relative *ALNS* transcript levels in WT, *alns-1*, and the three complementation lines, using *PPR* as a reference gene. Total RNA was extracted from 10-day-old seedlings grown as above. Values represent means ± SEM of three biological replicates (one-way ANOVA followed by Dunnett's multiple-comparison test; an asterisk (*) indicates $P < 0.05$ vs. WT).

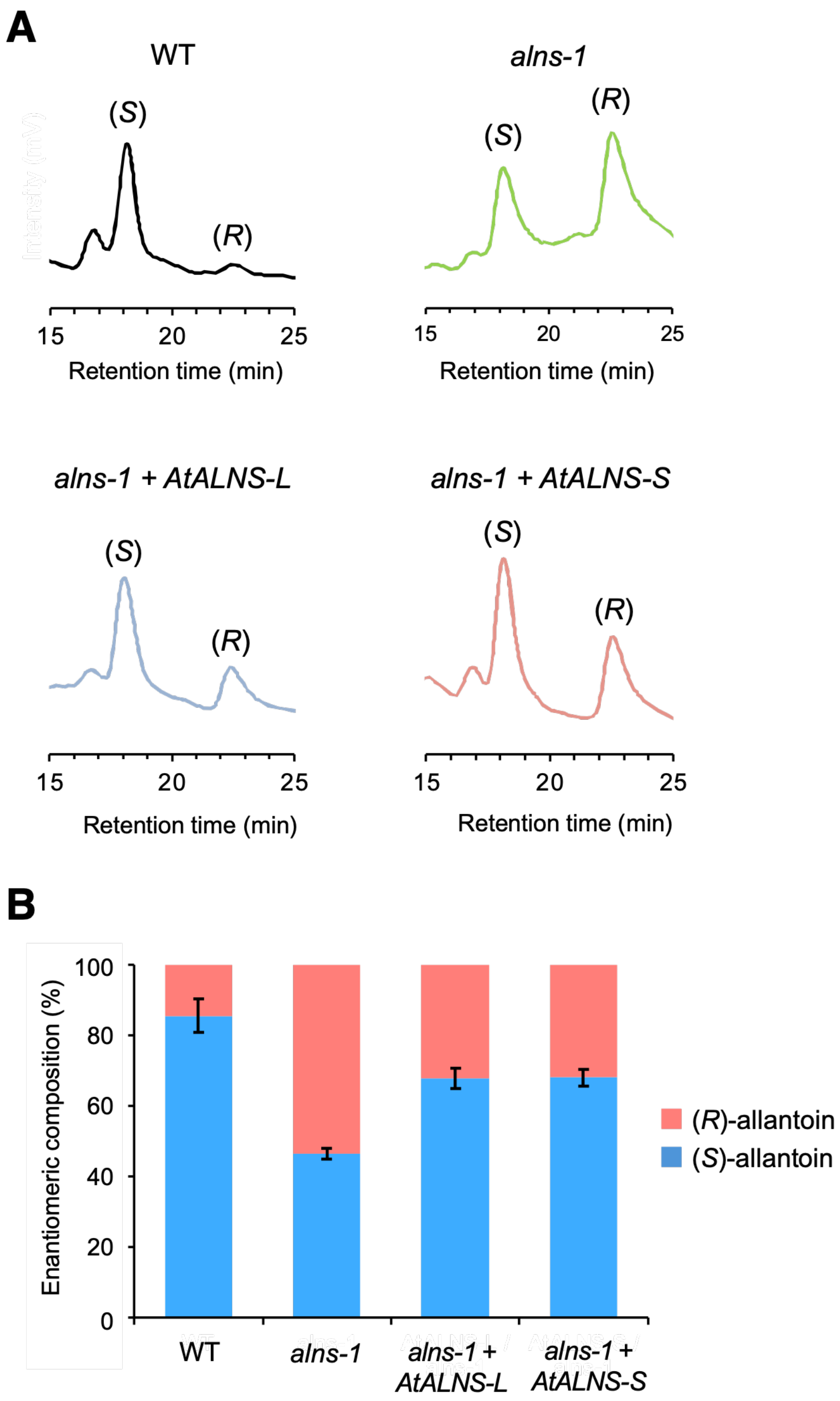

Figure S5. Enantiomeric composition of allantoin in wild-type, *alns-1*, and *alns-1* lines expressing *AtALNS* splice-variant constructs.

A. Representative chiral HPLC chromatograms of allantoin from 32-day-old plants of each genotype. Separation and peak assignment were performed as described in Figure S2. WT, wild-type.

B. Relative proportions of (*S*)- and (*R*)-allantoin. See Figure S4B for details about the expression constructs. Values represent means ± SEM of three biological replicates.

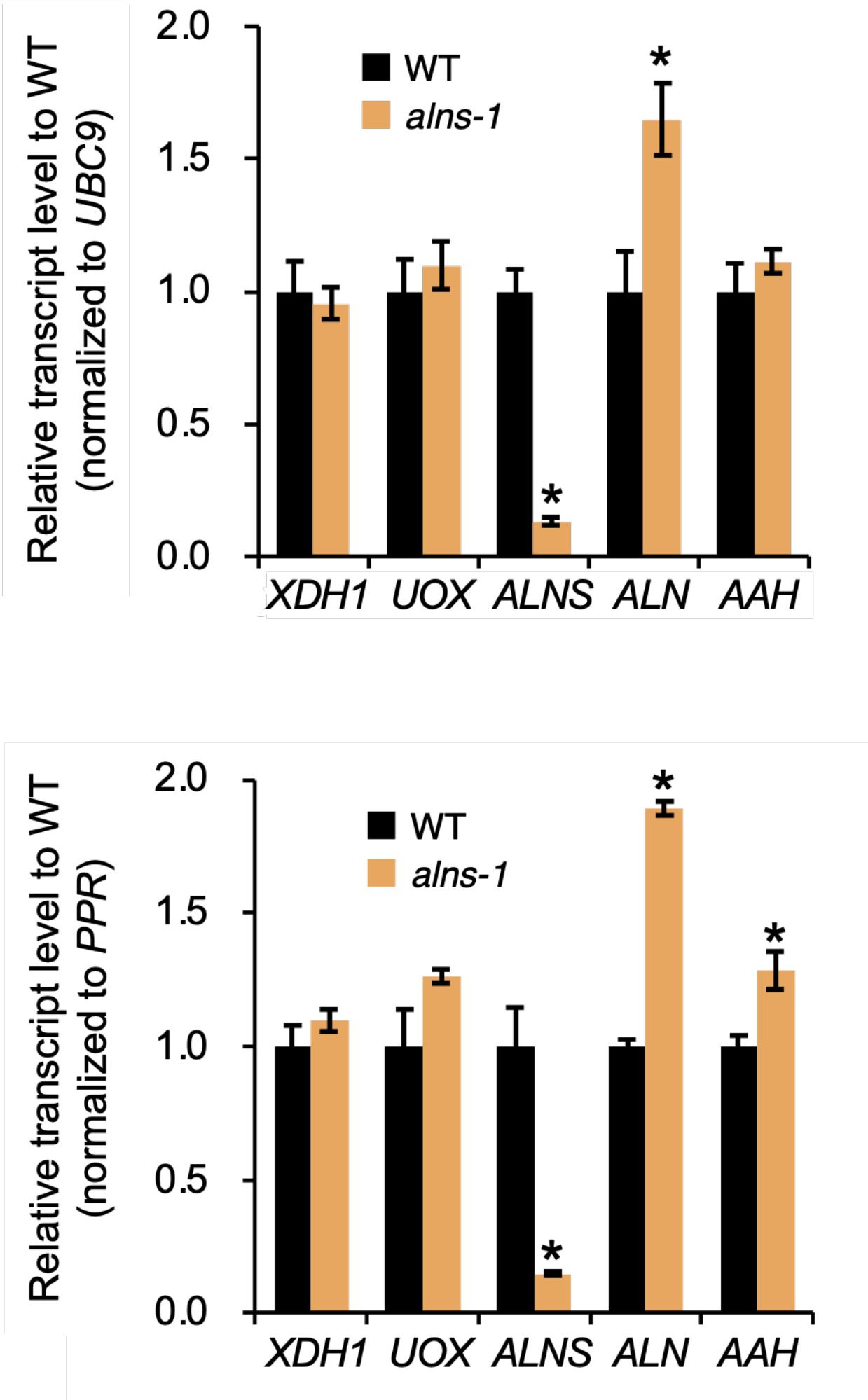

Figure S6. Relative expression levels of genes encoding purine catabolic enzymes involved in allantoin biosynthesis and degradation in wild-type and *alns-1* lines.

Total RNA for quantitative reverse-transcription PCR was extracted from 14-day-old seedlings grown under the standard conditions described in Materials and Methods in the main text. The same expression data were normalized independently using two reference genes, *UBC9* (top) and *PPR* (bottom). Relative transcript levels are shown with wild type (WT) set to 1. Primer sequences are listed in Table S5. *XDH1*, *XANTHINE DEHYDROGENASE 1* (AT4G34890); *UOX*, *URATE OXIDASE* (AT2G26230); *ALNS*, *ALLANTOIN SYNTHASE* (AT5G58220); *ALN*, *ALLANTOINASE* (AT4G04955); *AAH*, *ALLANTOIN AMIDOHYDROLASE* (AT4G20070). The reactions catalyzed by enzymes encoded by these genes are shown in Figure 1. Values represent means ± SEM of three biological replicates. Statistical significance was determined using Student's *t*-test; an asterisk (*) indicates $P < 0.05$ versus WT.

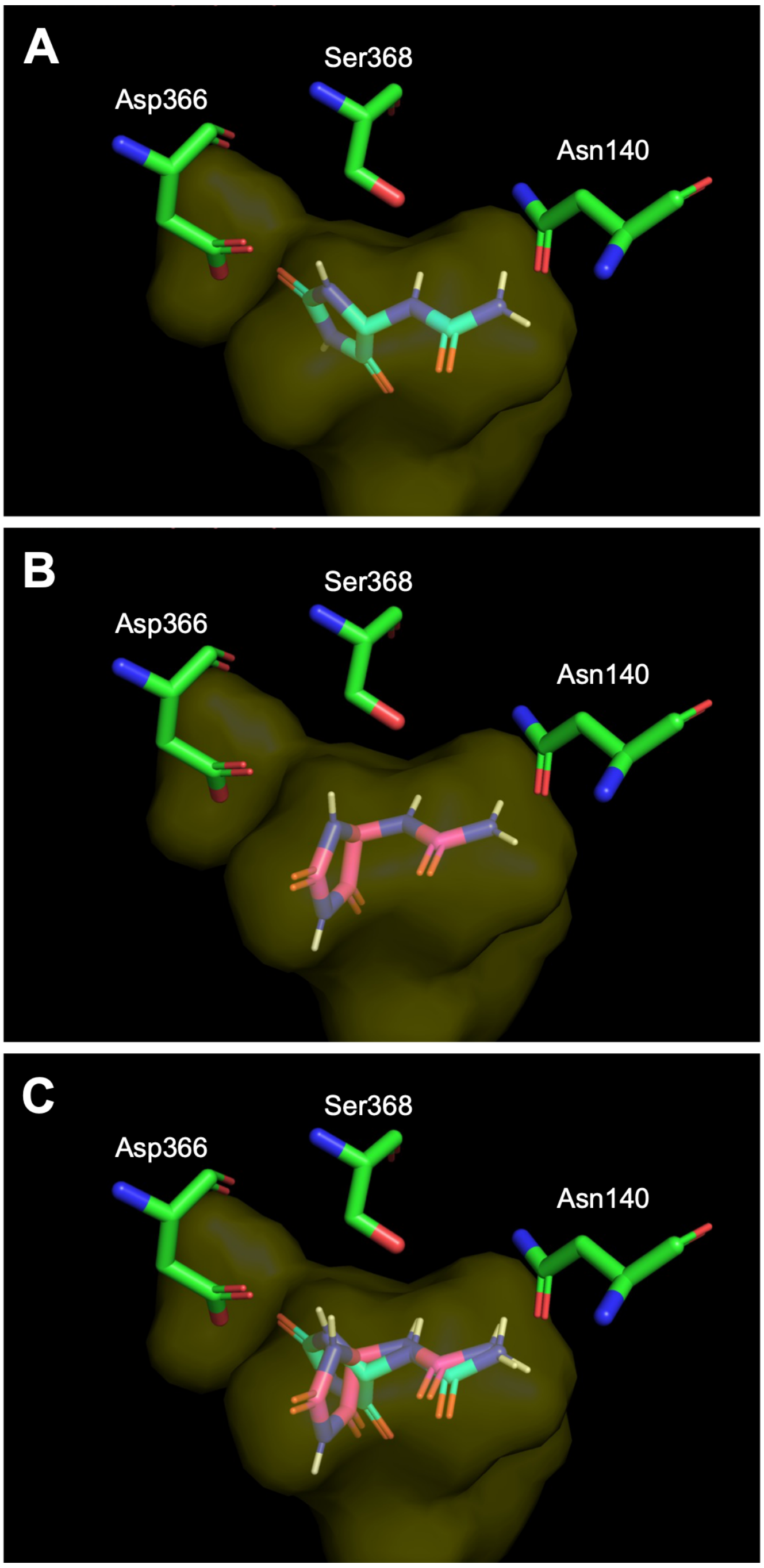

Figure S7. Predicted binding modes of (*S*)- and (*R*)-allantoin in the putative substrate-binding region of AtALN.

(*S*)- and (*R*)-allantoin are shown with cyan and magenta carbon atoms, respectively. Asn140, Asp366, and Ser368, the conserved residues corresponding to substrate-interacting residues identified in a bacterial allantoinase (Kim *et al*., 2009), are shown with green carbon atoms. The semitransparent molecular surface represents the putative substrate-binding pocket.

A. Docking pose of (*S*)-allantoin (model 4, selected based on its proximity to Asn140, Asp366, and Ser368; see Table S4).

B. Top-ranked docking pose of (*R*)-allantoin (model 1).

C. Superposition of the two poses. The two enantiomers show broadly similar overall positioning within the pocket but differ in the orientation of the hydantoin ring.

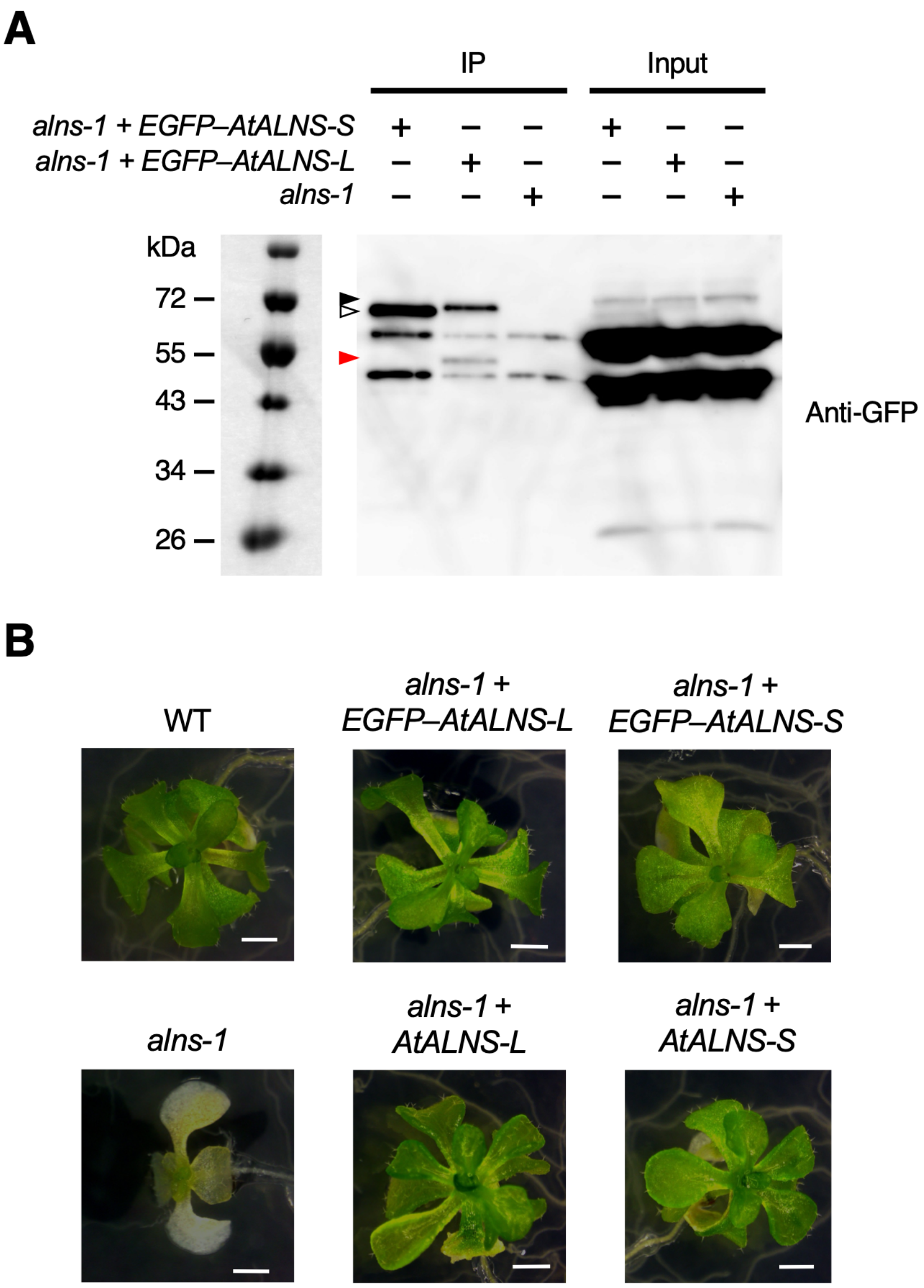

Figure S8. Both EGFP–AtALNS-L and EGFP–AtALNS-S rescue the defective growth of *alns-1* supplied with xanthosine as the sole nitrogen source.

A. EGFP–ALNS-S and EGFP–ALNS-L protein accumulation in transgenic *alns-1* plants. Immunoprecipitation (IP) was performed using 1 mg of total proteins extracted from 6-day-old seedlings with agarose beads conjugated to anti-GFP antibodies. Immunoblot analysis of input and IP fractions was performed using anti-GFP antibodies. Forty micrograms of total protein were loaded for the input samples. Open and closed arrowheads indicate EGFP–ALNS-S and EGFP–ALNS-L, respectively. Red arrowhead denotes a band observed specifically in the EGFP–ALNS-L sample, possibly corresponding to a processed EGFP–OHCUD–PTS2 fragment lacking the HIUH domain.

B. Representative images of each genotype. Plants were grown 45 days: 2 days on half-strength MS solid medium followed by 43 days on nitrogen-free half-strength MS solid medium supplemented with 5 mM xanthosine. The results for mutants expressing native *AtALNS-S* or *AlALNS-L* cDNA are shown for comparison. See Figures S1A and S4B for details about the fusion and native expression constructs, respectively. Scale bars, 1 mm.

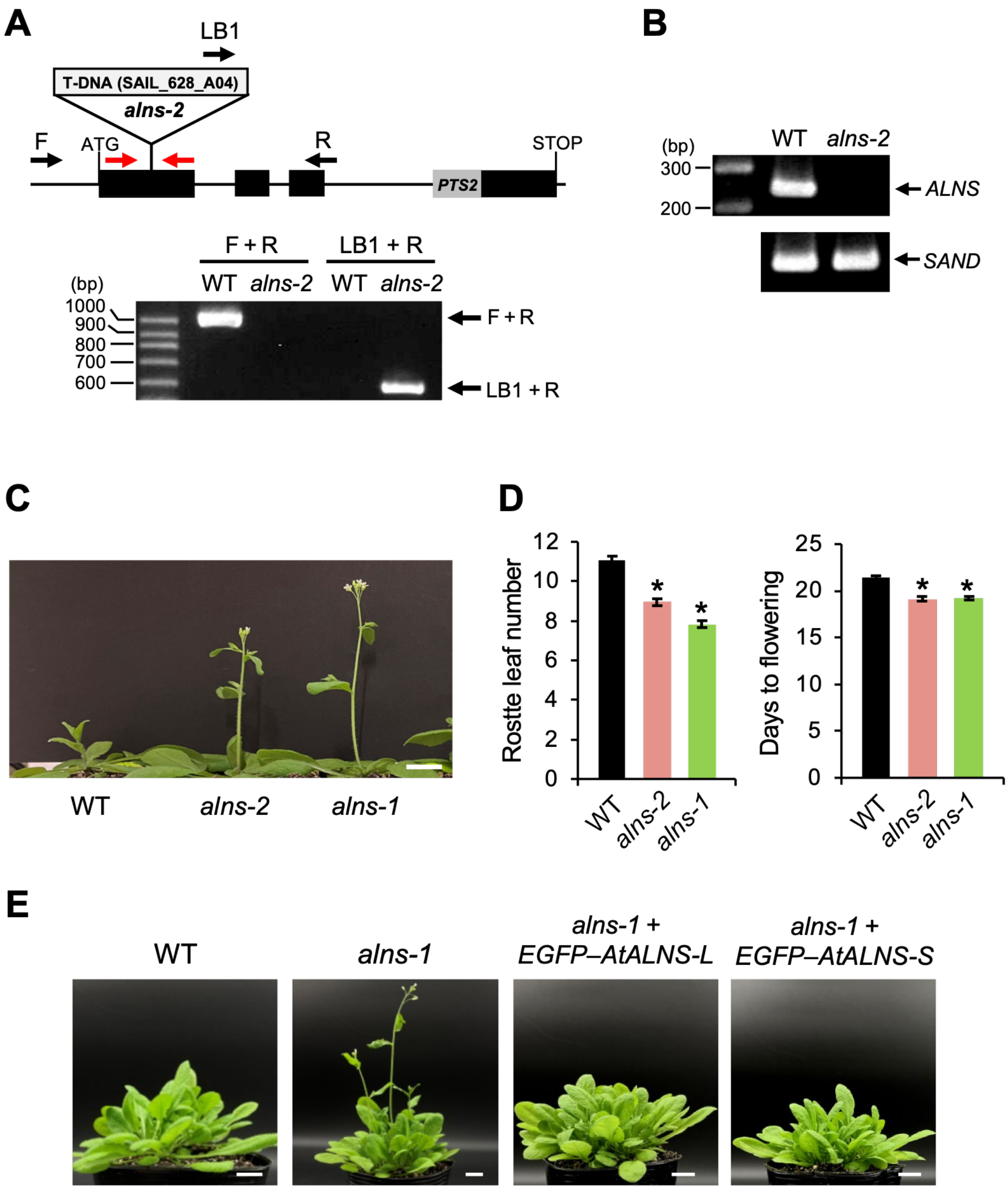

Figure S9. Characterization of the *alns-2* mutant and rescue of the *alns-1* early-flowering phenotype by EGFP–AtALNS-L and EGFP–AtALNS-S

A. Schematic representation of the *AtALNS* gene structure showing the position of the T-DNA insertion and the corresponding SAIL line identifier (upper panel). Arrows indicate primers used for genotyping (black) and expression analysis (red). Primer sequences are listed in Table S5. PCR genotyping is shown below. The expected sizes of the amplified products are 1044 bp for wild-type (WT) and 582 bp for *alns-2*.

B. Conventional reverse-transcription PCR of *AtALNS* transcripts in WT and *alns-2*, with *SAND* used as an internal control. The expected sizes of the amplified products are 247 bp for *ALNS* and 244 bp for *SAND*. Total RNA was extracted from 10-day-old seedlings grown under the standard conditions described in Materials and Methods in the main text.

C. Representative growth of WT, *alns-1*, and *alns-2* plants at 21 days under long-day conditions. Scale bars, 1 cm.

D. Number of days to flowering (left) and total rosette leaf number at flowering (right). Flowering was defined as the stage when the primary inflorescence stem reached 10 mm in height. Values represent means ± SEM of three biological replicates. Statistical significance was determined by one-way ANOVA followed by Dunnett's multiple-comparison test; an asterisk (*) indicates $P <$ 0.05 versus WT.

E. Representative flowering phenotypes at 24 days under long-day conditions. See Figures S1A and S8A for details of the constructs used for fusion protein expression and immunoblot analysis of the corresponding fusion proteins, respectively. Scale bars, 1 cm.

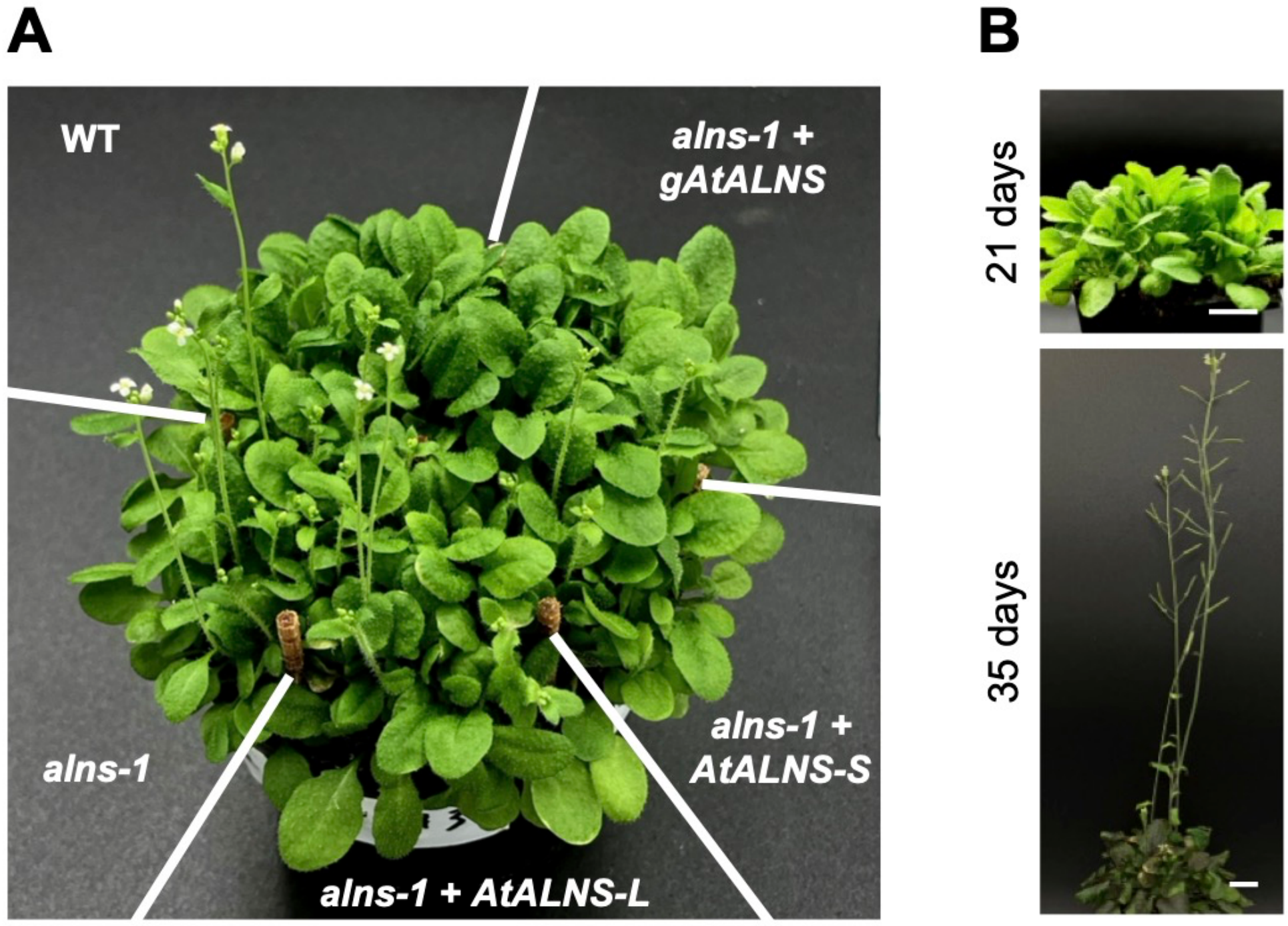

Figure S10. Rescue of the *alns-1* early-flowering phenotype by *AtALNS* splice-variant constructs and a genomic construct

A. Approximately 50 seeds per genotype were sown in soil, and the plants were grown for 21 days under long-day conditions. The experiment was performed twice. See Figure S4B for details about the expression constructs. WT, wild type.

B. Delayed flowering of *alns-1* plants expressing *gAtALNS*. Representative flowering phenotypes are shown at 21 and 35 days under long-day conditions. Scale bars, 1 cm.

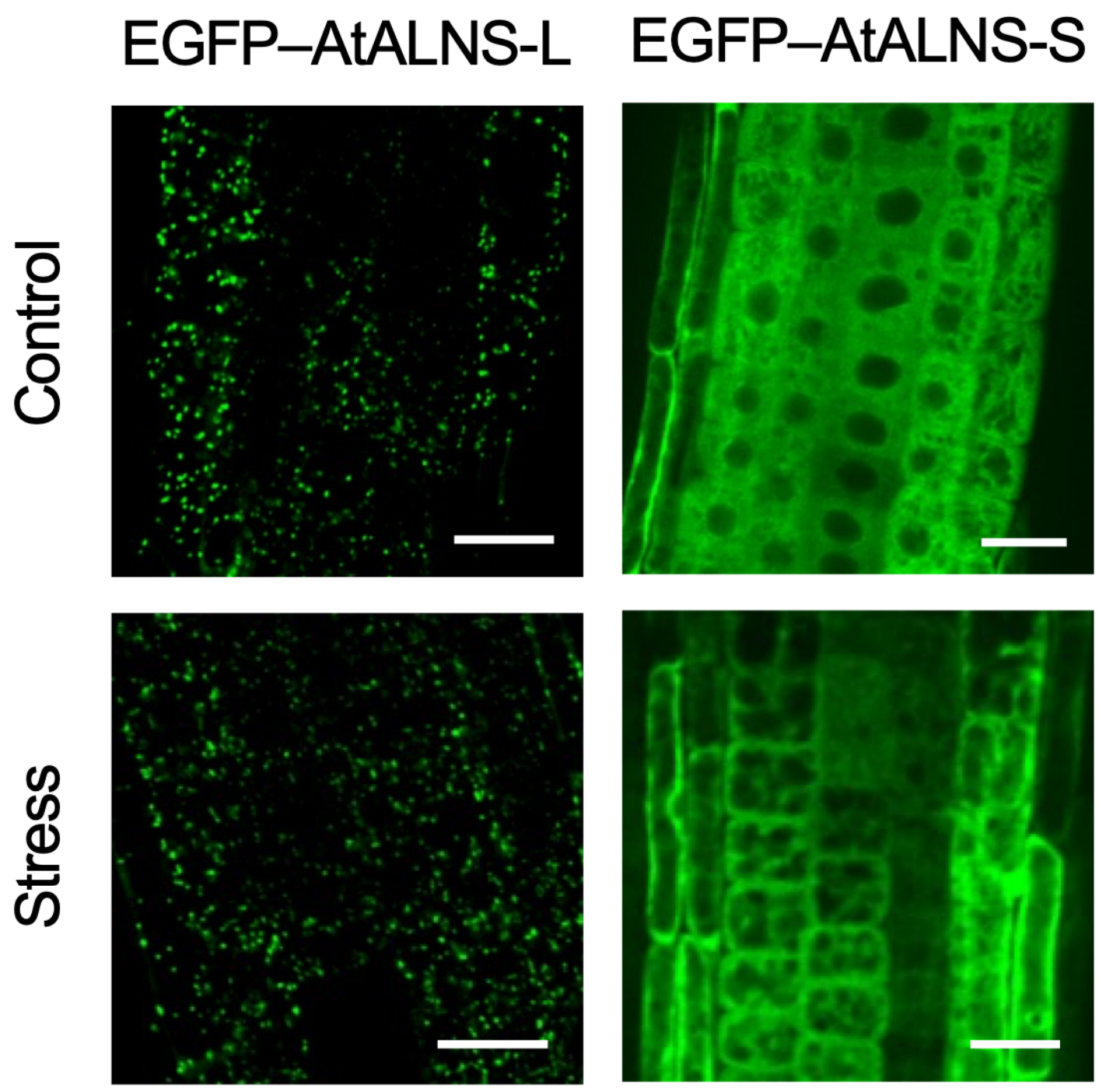

Figure S11. Subcellular localization of EGFP–AtALNS-L and EGFP–AtALNS-S under salt stress conditions.

Representative confocal images of Arabidopsis roots under control conditions (upper panels) and salt stress conditions (lower panels) are shown. Two-day-old seedlings were transferred in parallel to half-strength MS solid medium without added NaCl or supplemented with 100 mM NaCl and incubated for 24 h. Scale bars, 20 μm. See Figure S1A for details of the ALNS fusion constructs.

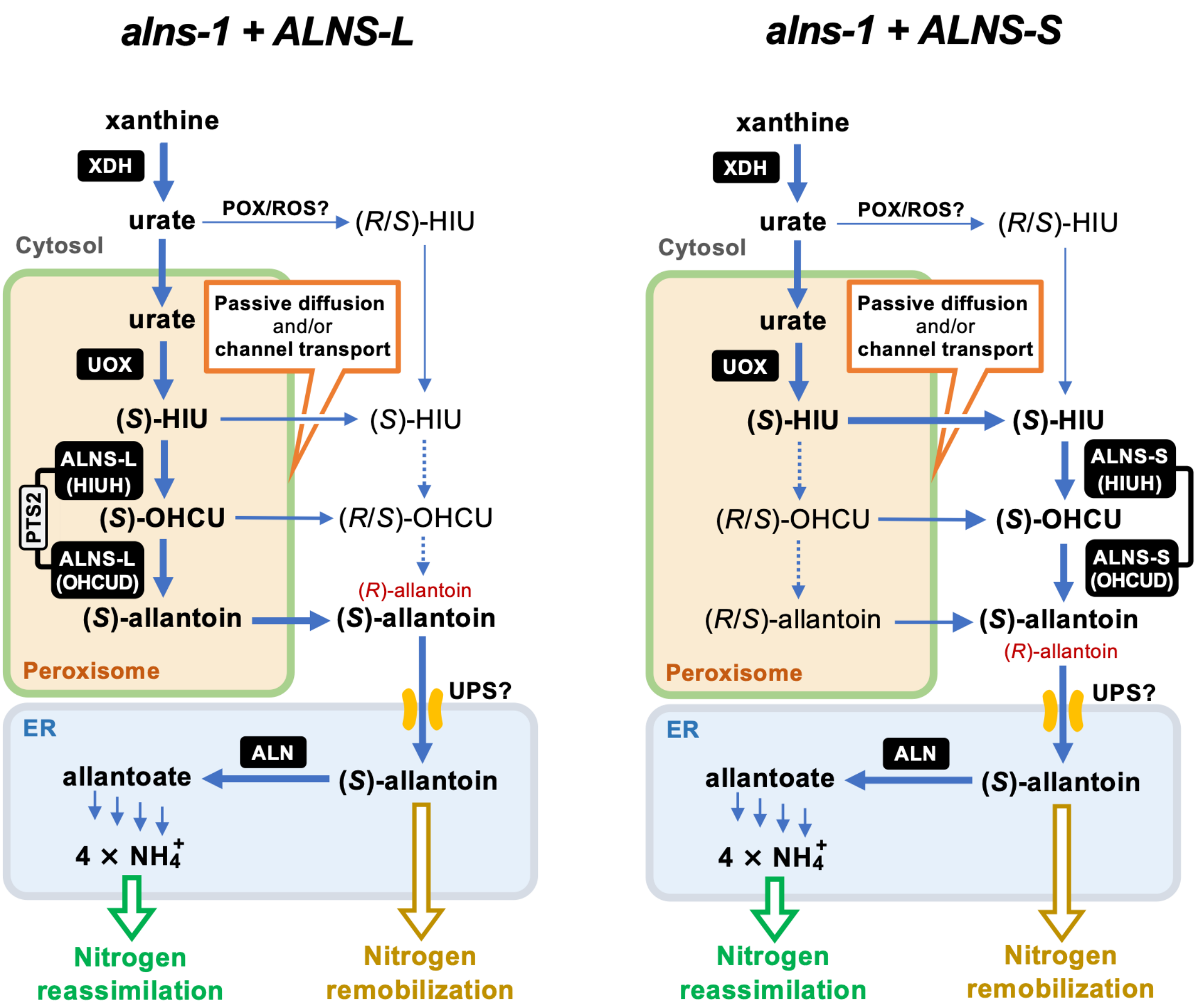

Figure S12. Working models of purine metabolism in *alns-1* mutants expressing *AtALNS* splice-variant constructs.

When either ALNS-L or ALNS-S is functional in the *alns-1* mutant, stereospecific (*S*)-allantoin biosynthesis is restored in the peroxisome or cytosol, respectively, thereby reducing the formation of racemic allantoin. Metabolite passage across the peroxisomal membrane may occur through passive diffusion, transport via unidentified channel-forming proteins, or both. HIU and possibly OHCU may therefore become available in the cytosol by leakage from peroxisomes, with minor contributions from chemical or enzymatic urate oxidation associated with ROS scavenging. Minor amounts of (*R*)-allantoin, consistent with the enantiomeric analysis, are also indicated in the cytosolic allantoin pool. See Figure 9 for the corresponding models for wild-type and *alns* mutant lines. Solid arrows indicate enzymatic reactions or metabolite transport, whereas dashed arrows indicate spontaneous non-enzymatic reactions. Arrow thickness schematically represents the proposed relative magnitude of metabolic or transport fluxes. Abbreviations: ALN, allantoinase; ER, endoplasmic reticulum; POX, putative urate peroxidase; ROS, reactive oxygen species; UPS, ureide permease.

Table S1. Domain architecture and predicted peroxisomal targeting signals (PTSs) of allantoin biosynthetic enzymes (ALNS, HIUH, and OHCUD) in Archaeplastida

Major lineage	Species (major group or lineage)	Domain architecture[a]	PTS[b]	Accession no.	Database / Method[c]	*E*-value	Reference
Angiosperms	*Arabidopsis thaliana* (eudicot)	OHCUD-HIUH (ALNS-L)	PTS2 (**RL**RI**I**GG**HL**)	NP_200630.1	NCBI		Ramazzina *et al.*, 2006
		OHCUD-HIUH (ALNS-S)	n.d.	NP_001032094.1	NCBI		Lamberto *et al.*, 2010
	Erigeron breviscapus (eudicot)	OHCUD-HIUH (ALNS-L)	PTS2 (**RV**AI**L**GG**HL**)	GDQF01113739.1	NCBI (TSA) / tBLASTn	5e−112	
		OHCUD-HIUH (ALNS-S)	n.d.	GDQF01113740.1	NCBI (TSA) / tBLASTn	2e−105	
	Fragaria vesca (eudicot)	OHCUD-HIUH (ALNS-L)	PTS2 (**RV**SI**I**GG**HL**)	GJFN01009879.1	NCBI (TSA) / tBLASTn	4e−93	
		OHCUD-HIUH (ALNS-S)	n.d.	EX668013.1	NCBI (EST) / tBLASTn	2e−96	
	Populus trichocarpa (eudicot)	OHCUD-HIUH (ALNS-L)	PTS2 (**RV**SI**I**SG**HL**)	Potri.002G238300.4	Phytozome / BLASTP	1e−124[d]	Lamberto *et al.*, 2010[d]
		OHCUD-HIUH (ALNS-S)	n.d.	EEE81034.1	NCBI		Lamberto *et al.*, 2010
	Vitis vinifera (eudicot)	OHCUD-HIUH (ALNS-L)	PTS2 (**RV**SI**I**GA**HL**)	CAO23132.1	NCBI		Lamberto *et al.*, 2010
		OHCUD-HIUH (ALNS-S)	n.d.	XP_002268834.1	NCBI		Lamberto *et al.*, 2010
	Anthurium andraeanum (monocot)	OHCUD-HIUH (ALNS-L)	PTS2 (**RI**GV**I**GG**HL**)	GBKP01002889.1	NCBI (TSA) / tBLASTn	8e−113	
		OHCUD-HIUH (ALNS-S)	n.d.	GGPS01045710.1	NCBI (TSA) / tBLASTn	2e−104	
	Lilium longiflorum (monocot)	OHCUD-HIUH (ALNS-L)	PTS2 (**RI**GV**I**GA**HL**)	GIUQ01002611.1	NCBI (TSA) / tBLASTn	1e−106	
		OHCUD-HIUH (ALNS-S)	n.d.	GIUQ01002610.1	NCBI (TSA) / tBLASTn	4e−100	
	Oryza sativa (monocot)	OHCUD-HIUH (ALNS-L)	PTS2 (**RM**RI**I**GA**HL**)	NP_001050277.1	NCBI		Lamberto *et al.*, 2010
		OHCUD-HIUH (ALNS-S)	n.d.	AAR06356.1	NCBI		Lamberto *et al.*, 2010
	Triticum aestivum (monocot)	OHCUD-HIUH (ALNS-L)	PTS2 (**RM**RI**I**GA**HL**)	BQ295224	NCBI		Reumann *et al.*, 2007
		OHCUD-HIUH (ALNS-S)	n.d.	BE403432	NCBI		Reumann *et al.*, 2007
		OHCUD-HIUH (ALNS-S)	n.d.	BJ214926	NCBI		Reumann *et al.*, 2007
	Zea mays (monocot)	OHCUD-HIUH (ALNS-L)	PTS2 (**RI**RI**I**GA**HL**)	GECR01012710.1	NCBI (TSA) / tBLASTn	1e−49[d]	Lamberto *et al.*, 2010[d]
		OHCUD-HIUH (ALNS-S)	n.d.	GGGX01018049.1	NCBI (TSA) / tBLASTn	1e−91[d]	Lamberto *et al.*, 2010[d]
	Piper methysticum (Basal angiosperm)	OHCUD-HIUH (ALNS-L)	PTS2 (**RL**LI**L**GA**HL**)	GHAC01112288.1	NCBI (TSA) / tBLASTn	5e−108	
		OHCUD-HIUH (ALNS-S)	n.d.	GHAC01112287.1	NCBI (TSA) / tBLASTn	2e−103	
Gymnosperms	*Ephedra trifurca* (Gnetidae)	OHCUD-HIUH (ALNS-L)	PTS2 (**RL**KQ**I**GG**HL**)	GBKT01011712.1	NCBI (TSA) / tBLASTn	4e−101	
	Picea abies (Pinidae)	OHCUD-HIUH (ALNS-L)	PTS2 (**RL**NH**I**GA**HL**)	GKLE01026854.1	NCBI (TSA) / tBLASTn	7e−89	
	Picea glauca (Pinidae)	OHCUD-HIUH (ALNS-L)	PTS2 (**RL**NH**I**GA**HL**)	GGJI01007790.1	NCBI (TSA) / tBLASTn	3e−88[d]	Lamberto *et al.*, 2010[d]
	Pinus albicaulis (Pinidae)	OHCUD-HIUH (ALNS-L)	PTS2 (**RL**NH**I**GA**HL**)	GDQR01015514.1	NCBI (TSA) / tBLASTn	2e−96	
	Pinus flexilis (Pinidae)	OHCUD-HIUH (ALNS-L)	PTS2 (**RL**NH**I**GA**HL**)	GDQE01013372.1	NCBI (TSA) / tBLASTn	4e−96	
	Pinus taeda (Pinidae)	OHCUD-HIUH (ALNS-L)	PTS2 (**RL**NH**I**GA**HL**)	GKVD01030262.1	NCBI (TSA) / tBLASTn	3e−92[d]	Lamberto *et al.*, 2010[d]
	Araucaria cunninghamii (Cupressidae)	OHCUD-HIUH (ALNS-L)	PTS2 (**RL**SQ**I**GA**HL**)	GCKF01041346.1	NCBI (TSA) / tBLASTn	2e−91	
	Cryptomeria japonica (Cupressidae)	OHCUD-HIUH (ALNS-L)	PTS2 (**RL**SQ**I**GG**HL**)	ICQR01022182.1	NCBI (TSA) / tBLASTn	2e−95	
	Sequoia sempervirens (Cupressidae)	OHCUD-HIUH (ALNS-L)	PTS2 (**RL**SQ**I**EG**HL**)	GIBU01037049.1	NCBI (TSA) / tBLASTn	3e−71	
	Taxus chinensis (Cupressidae)	OHCUD-HIUH (ALNS-L)	PTS2 (**RL**SQ**I**GG**HL**)	KA390740.1	NCBI (TSA) / tBLASTn	8e−73	
	Thuja plicata (Cupressidae)	OHCUD-HIUH (ALNS-L)	PTS2 (**RL**SQ**I**GG**HL**)	Thupl.29379470s0024.1	Phytozome / BLASTP	3e−101	
	Cycas revoluta (Cycadidae)	OHCUD-HIUH (ALNS-L)	PTS2 (**RL**NQ**I**GA**HL**)	GBJU01034422.1	NCBI (TSA) / tBLASTn	4e−72	
	Cycas sphaerica (Cycadidae)	OHCUD-HIUH (ALNS-L)	PTS2 (**RL**NQ**I**GA**HL**)	GGZS01003777.1	NCBI (TSA) / tBLASTn	9e−87	
	Ginkgo biloba (Ginkgoidae)	OHCUD-HIUH (ALNS-L)	PTS2 (**RL**NQ**I**GA**HL**)	GGBW01015584.1	NCBI (TSA) / tBLASTn	1e−94	

(*continues*)

Table S1. (*continued*)

Major lineage	Species (major group or lineage)	Domain architecture[a]	PTS[b]	Accession no.	Database / Method[c]	*E*-value	Reference
Monilophytes	*Brainea insignis* (Eupolypods)	OHCUD-HIUH (ALNS-S)	n.d.	GFUE01052070.1	NCBI (TSA) / tBLASTn	2e−82	
	Dryopteris erythrosora (Eupolypods)	OHCUD-HIUH (ALNS-S)	n.d.	GHEW01015885.1	NCBI (TSA) / tBLASTn	3e−80	
	Ceratopteris richardii (Pteridaceae)	OHCUD-HIUH (ALNS-L)	PTS2 (**RL**NQ**F**LG**HL**)	HANB01249884.1	NCBI (TSA) / tBLASTn	6e−83	
	Pteris vittata (Pteridaceae)	OHCUD-HIUH (ALNS-L)	PTS2 (**RL**GQ**V**LG**HL**)	GGXV01190287.1	NCBI (TSA) / tBLASTn	6e−62	
	Azolla filiculoides (Salviniaceae)	OHCUD-HIUH (ALNS-L)	PTS2 (**RM**GQIAG**HL**)	GBTV01014965.1	NCBI (TSA) / tBLASTn	4e−80	
	Lygodium japonicum (Lygodiaceae)	OHCUD-HIUH (ALNS-L)	PTS2 (**RV**GQIGA**HL**)	FX972116.1	NCBI (TSA) / tBLASTn	2e−83	
	Botrychium lunaria (Ophioglossaceae)	OHCUD-HIUH (ALNS-S)	n.d.	GIJZ01009288.1	NCBI (TSA) / tBLASTn	1e−60	
Lycophytes	*Isoetes lacustris* (Isoetaceae)	OHCUD-HIUH (ALNS-L)	PTS2 (**RL**NKIQA**HV**)	GIOA01057218.1	NCBI (TSA) / tBLASTn	2e−30	
	Selaginella denticulata (Selaginellaceae)	OHCUD-HIUH (ALNS-L)	PTS2 (**RL**NKIEA**HV**)	GIMH01041835.1	NCBI (TSA) / tBLASTn	3e−83	
	Selaginella moellendorffii (Selaginellaceae)	OHCUD-HIUH (ALNS-L)	PTS2 (**RL**SKIEA**HV**)	FE444796.1	NCBI (EST) / tBLASTn	3e−22	
Bryophytes	*Bryum argenteum* (moss)	OHCUD-HIUH (ALNS-S)	n.d.	GCZP01012556.1	NCBI (TSA) / tBLASTn	1e−82	
	Dicranum scoparium (moss)	OHCUD-HIUH (ALNS-S)	n.d.	HANF01025301.1	NCBI (TSA) / tBLASTn	1e−81	
	Physcomitrella patens (moss)	OHCUD-HIUH (ALNS-S)	n.d.	XP_001775390.1	NCBI		Lamberto *et al.*, 2010
	Pohlia nutans (moss)	OHCUD-HIUH (ALNS-S)	n.d.	GACA01042657.1	NCBI (TSA) / tBLASTn	9e−83	
	Sanionia uncinata (moss)	OHCUD-HIUH (ALNS-S)	n.d.	GKBN01002878.1	NCBI (TSA) / tBLASTn	1e−78	
	Marchantia paleacea (liverwort)	OHCUD-HIUH (ALNS-S)	n.d.	GFGJ01007739.1	NCBI (TSA) / tBLASTn	5e−77	
	Marchantia polymorpha (liverwort)	OHCUD-HIUH (ALNS-S)	n.d.	ICPF01017664.1	NCBI (TSA) / tBLASTn	2e−76	
	Anthoceros agrestis (hornwort)	OHCUD-HIUH (ALNS-L)	PTS2 (**RL**TQIEA**HL**)	TWUW_scaffold_2049370	CNGB / BLASTP	1e−85	
	Megaceros tosanus (hornwort)	OHCUD-HIUH (ALNS-L)	PTS2 (**RL**SQIGA**HV**)	UCRN_scaffold_2051854	CNGB / BLASTP	1e−83	
	Phaeoceros carolinianus (hornwort)	OHCUD-HIUH (ALNS-L)	PTS2 (**RL**NQIEA**HI**)	WCZB_scaffold_2117093	CNGB / BLASTP	1e−93	
	Phaeomegaceros coriaceus (hornwort)	OHCUD-HIUH (ALNS-L)	PTS2 (**RL**SQIGS**HF**)	AKXB_scaffold_2066062	CNGB / BLASTP	2e−85	
Streptophyte algae	*Mougeotiopsis calospora* (Serritaeniales)	OHCUD-HIUH (ALNS-S)	n.d.	GJZN01026065.1	NCBI (TSA) / tBLASTn	1e−74	
	Serritaenia testaceovaginata[e] (Serritaeniales)	OHCUD-HIUH (ALNS-L)	PTS2 (**RL**RQ**V**RD**HV**)	HCDJ01042784.1	NCBI (TSA) / tBLASTn	2e−22	
	Cylindrocystis crassa (Zygnematales)	OHCUD-HIUH (ALNS-S)	n.d.	HCDI01057241.1	NCBI (TSA) / tBLASTn	6e−65	
	Spirogyra pratensis (Spirogyrales)	OHCUD-HIUH (ALNS-L)	PTS2 (**RL**GNITA**HL**)	GBSM01007049.1	NCBI (TSA) / tBLASTn	6e−71	
	Coleochaete orbicularis (Coleochaetales)	OHCUD-HIUH (ALNS-S)	n.d.	GBSL01035708.1	NCBI (TSA) / tBLASTn	1e−67	
	Klebsormidium flaccidum (Klebsormidiales)	OHCUD-HIUH (ALNS-L)	PTS2 (**RL**AQIEN**HL**)	GBSO01004786.1	NCBI (TSA) / tBLASTn	3e−31	
	Mesostigma viride (Mesostigmatales)	OHCUD-HIUH (ALNS-L)	PTS2 (**RL**QHISA**HV**)	GBEK01017148.1	NCBI (TSA) / tBLASTn	9e−43	
Chlorophytes	*Chlamydomonas reinhardtii* (Chlorophyceae)	OHCUD-HIUH (ALNS-L)	PTS2 (**RA**EQ**V**LT**HL**)	XP_001699572.1	NCBI		Lamberto *et al.*, 2010
	Volvox carteri f. nagariensis (Chlorophyceae)	OHCUD-HIUH (ALNS-L)	PTS2 (**RA**EQ**V**LT**HL**)	XP_002947784.1	NCBI		Oh *et al.*, 2018
	Chlorella variabilis (Trebouxiophyceae)	HIUH	PTS2 (**RA**GR**L**LA**QL**)	XP_005844568.1	NCBI (ClusteredNR) / BLASTP	4e−22[d]	Kechasov *et al.*, 2020[d]
		OHCUD	n.d.	XP_005844569.1	NCBI (ClusteredNR) / BLASTP	1e−19[d]	Kechasov *et al.*, 2020[d]
	Chlorella sorokiniana (Trebouxiophyceae)	HIUH	n.d.	GCUV01011967.1_1	NCBI (TSA) / tBLASTn	1e−31[d]	Kechasov *et al.*, 2020[d]
		OHCUD	n.d.	GCUV01011967.1_2	NCBI (TSA) / tBLASTn	1e−31[d]	Kechasov *et al.*, 2020[d]
	Micromonas commoda (Prasinophytes)	HIUH	n.d.	XP_002502299.1	NCBI		Oh *et al.*, 2018
		OHCUD	PTS1 (**AKL**)	XP_002501565.1	NCBI		Oh *et al.*, 2018

(*continues*)

Table S1. (*continued*)

Major lineage	Species (major group or lineage)	Domain architecture[a]	PTS[b]	Accession no.	Database / Method[c]	*E*-value	Reference
Rhodophytes	*Chondrus crispus*	HIUH	n.d.	XP_005715913.1	NCBI		Oh *et al.*, 2018
		OHCUD	n.d.	XP_005714641.1	NCBI		Oh *et al.*, 2018
	Porphyra umbilicalis	HIUH	n.d.	Pum0317s0010.1	Phytozome / BLASTP	5e−18	
		OHCUD	n.d.	Pum0085s0025.1	Phytozome / BLASTP	3e−4	
	Porphyridium purpureum	HIUH	n.d.	GJVU01005269.1	NCBI (TSA) / tBLASTn	6e−09	
		OHCUD	n.d.	GJVU01001188.1	NCBI (TSA) / tBLASTn	9e−14	
	Rhodosorus marinus	HIUH	n.d.	HBEK01013719.1	NCBI (TSA) / tBLASTn	9e−16	
		OHCUD	n.d.	HBHW01015301.1	NCBI (TSA) / tBLASTn	5e−14	
Glaucophytes	*Cyanoptyche gloeocystis*	HIUH	PTS2 (**RL**GC**V**IR**HL**)	JKHA_scaffold_2113470	CNGB / tBLASTn	2e−15	
		OHCUD	PTS1 (P**KL**)	JKHA_scaffold_2118105	CNGB / tBLASTn	1e−16	
	Glaucocystis cf. nostochinearum	HIUH	PTS2 (**RL**EK**I**AK**HL**)	POOW_scaffold_2048914	CNGB / tBLASTn	2e−18	
		OHCUD	PTS1 (P**KL**)	POOW_scaffold_2010133	CNGB / tBLASTn	9e−20	
	Gloeochaete wittrockiana	HIUH	PTS2 (**RL**TT**L**TR**HF**)	HBOJ01004260.1	NCBI (TSA) / tBLASTn	3e−14	
		OHCUD	PTS1 (P**KL**)	HBOJ01057126.1	NCBI (TSA) / tBLASTn	7e−21	

[a]Enzymes lacking an apparent peroxisomal targeting signal (PTS) are highlighted in red. For bifunctional two-domain enzymes (ALNS-L and ALNS-S), variant names are shown in parentheses following the domain architecture.

[b]PTS motifs were predicted using the PTS1 Predictor (https://mendel.imp.ac.at/pts1/) and by manual inspection of the PTS2 consensus motif RΦXXΦXX(H/Q)Φ, with Φ denoting a hydrophobic residue and X any residue (Kunze, 2020). Corresponding sequences are shown in parentheses, with residues matching the canonical PTS1 motif (S/A/C)(K/R/H)L or the PTS2 consensus motif indicated in boldface. n.d., not detected. When present, PTS1 is located at the carboxyl terminus of OHCUD and PTS2 at the amino terminus of HIUH in single-domain enzymes, whereas in two-domain ALNS, PTS2 is found exclusively in the interdomain region.

[c]Unless otherwise noted, ALNS sequences were retrieved using the indicated databases and programs, with two Arabidopsis sequences (NP_200630.1 and NP_001032094.1) used as queries. NCBI, National Center for Biotechnology Information; TSA, Transcriptome Shotgun Assembly; EST, Expressed Sequence Tag; CNGB, China National GeneBank.

[d]Accession numbers cited in these references were either no longer valid or not provided; therefore, the corresponding sequences were retrieved for this study.

[e]Formerly known as *Mesotaenium testaceovaginatum.*

Table S2. Occurrence and predicted subcellular localization of xanthine dehydrogenases (XDHs) in Archaeplastida

Major lineage	Species (major group or lineage)[a]	Subcellular localization[c]	Accession no.	Database / Method[d]	*E*-value
Angiosperms	*Arabidopsis thaliana* (eudicot)	Cytoplasm	NP_195215.2	NCBI	
		Cytoplasm	NP_195216.2	NCBI	0
	Erigeron breviscapus (eudicot)	Cytoplasm	GDQF01096815.1	NCBI (TSA) / tBLASTn	0
	Fragaria vesca (eudicot)	Cytoplasm	GJFN01007015.1	NCBI (TSA) / tBLASTn	0
	Populus trichocarpa (eudicot)	Cytoplasm	Potri.009G054600.1	Phytozome / BLASTP	0
	Vitis vinifera (eudicot)	Cytoplasm	HAOE01027004.1	NCBI (TSA) / tBLASTn	0
	Anthurium andraeanum (monocot)	Cytoplasm	GGPS01020907.1	NCBI (TSA) / tBLASTn	0
	Lilium longiflorum (monocot)	Cytoplasm	GIUQ01019757.1	NCBI (TSA) / tBLASTn	0
	Oryza sativa (monocot)	Cytoplasm	GFMS01000262.1	NCBI (TSA) / tBLASTn	0
	Triticum aestivum (monocot)	Cytoplasm	GIJS01086465.1	NCBI (TSA) / tBLASTn	0
	Zea mays (monocot)	Cytoplasm	GGGX01000571.1	NCBI (TSA) / tBLASTn	0
	Piper methysticum (Basal angiosperm)	Cytoplasm	GHAC01134015.1	NCBI (TSA) / tBLASTn	0
Gymnosperms	*Ephedra trifurca* (Gnetidae)	Cytoplasm	GBKT01026887.1	NCBI (TSA) / tBLASTn	0
	Picea abies (Pinidae)	Cytoplasm	GGIG01013835.1	NCBI (TSA) / tBLASTn	0
	Picea glauca (Pinidae)	Cytoplasm	GGJI01001572.1	NCBI (TSA) / tBLASTn	0
	Pinus albicaulis (Pinidae)	Cytoplasm	GFLL01114438.1	NCBI (TSA) / tBLASTn	0
	Pinus flexilis (Pinidae)	Cytoplasm	GHWC02119074.1	NCBI (TSA) / tBLASTn	0
	Pinus taeda (Pinidae)	Cytoplasm	GKVF01010501.1	NCBI (TSA) / tBLASTn	0
	Araucaria cunninghamii (Cupressidae)	Cytoplasm	GCKF01046055.1	NCBI (TSA) / tBLASTn	0
	Cryptomeria japonica (Cupressidae)	Cytoplasm	IABV01014344.1	NCBI (TSA) / tBLASTn	0
	Sequoia sempervirens (Cupressidae)	Cytoplasm	GIBU01172762.1	NCBI (TSA) / tBLASTn	0
	Taxus chinensis (Cupressidae)	Cytoplasm	KA393625.1	NCBI (TSA) / tBLASTn	0
	Thuja plicata (Cupressidae)	Cytoplasm	Thupl.29377726s0006.1	Phytozome / BLASTP	0
	Cycas revoluta (Cycadidae)	Cytoplasm	GBJU01009071.1	NCBI (TSA) / tBLASTn	0
	Cycas sphaerica (Cycadidae)	Cytoplasm	GGZS01014230.1	NCBI (TSA) / tBLASTn	0
	Ginkgo biloba (Ginkgoidae)	Cytoplasm	GHLL01235322.1	NCBI (TSA) / tBLASTn	0
Monilophytes	*Brainea insignis* (Eupolypods)	Cytoplasm	GFUE01014142.1	NCBI (TSA) / tBLASTn	0
	Dryopteris erythrosora (Eupolypods)	Cytoplasm	GHEW01054746.1	NCBI (TSA) / tBLASTn	0
	Ceratopteris richardii (Pteridaceae)	Cytoplasm	GIXU01039241.1	NCBI (TSA) / tBLASTn	0
	Pteris vittata (Pteridaceae)	Cytoplasm	GGXV01155408.1	NCBI (TSA) / tBLASTn	0
	Azolla filiculoides (Salviniaceae)	Cytoplasm	GBTV01004877.1	NCBI (TSA) / tBLASTn	0
	Lygodium japonicum (Lygodiaceae)	Nucleus	FX950356.1	NCBI (TSA) / tBLASTn	5e−107
	Botrychium lunaria (Ophioglossaceae)	Cytoplasm	GIJZ01000441.1	NCBI (TSA) / tBLASTn	0
Lycophytes	*Isoetes lacustris* (Isoetaceae)	Cytoplasm	GIOA01069216.1	NCBI (TSA) / tBLASTn	0
	Selaginella denticulata (Selaginellaceae)	Cytoplasm	GIMH01010867.1	NCBI (TSA) / tBLASTn	0
	Selaginella moellendorffii (Selaginellaceae)	Cytoplasm	XP_002971411.1	NCBI (ClusteredNR) / BLASTP	0

(*continues*)

Table S2. (*continued*)

Major lineage	Species (major group or lineage)[a]	Subcellular localization[b]	Accession no.	Database / Method[c]	*E*-value
Bryophytes	*Bryum argenteum* (moss)	Cytoplasm	GCZP01039539.1	NCBI (TSA) / tBLASTn	0
	Dicranum scoparium (moss)	Cytoplasm	HANF01047445.1	NCBI (TSA) / tBLASTn	0
	Physcomitrella patens (moss)	Cytoplasm	XP_024357701.1	NCBI (ClusteredNR) / BLASTP	0
	Pohlia nutans (moss)	Cytoplasm	GACA01014309.1	NCBI (TSA) / tBLASTn	0
	Sanionia uncinate (moss)	Cytoplasm	GKBN01031230.1	NCBI (TSA) / tBLASTn	0
	Marchantia paleacea (liverwort)	Cytoplasm	GFGJ01000268.1	NCBI (TSA) / tBLASTn	0
	Marchantia polymorpha (liverwort)	Cytoplasm	ICPF01001514.1	NCBI (TSA) / tBLASTn	0
	Anthoceros agrestis (hornwort)	Cytoplasm	TWUW_scaffold_2052462	CNGB / tBLASTn	0
	Megaceros tosanus (hornwort)	Cytoplasm	UCRN_scaffold_2000566	CNGB / tBLASTn	0
	Phaeoceros carolinianus (hornwort)	Cytoplasm	WCZB_scaffold_2120128	CNGB / tBLASTn	0
	Phaeomegaceros coriaceus (hornwort)	Cytoplasm	AKXB_scaffold_2067804	CNGB / tBLASTn	0
Streptophyte algae	*Mougeotiopsis calospora* (Serritaeniales)	Cytoplasm	GJZN01001873.1	NCBI (TSA) / tBLASTn	0
	Serritaenia testaceovaginata[d] (Serritaeniales)	Cytoplasm	HCDJ01106060.1	NCBI (TSA) / tBLASTn	2e−159
	Cylindrocystis crassa (Zygnematales)	Cytoplasm	HCDI01046283.1	NCBI (TSA) / tBLASTn	0
	Spirogyra pratensis (Spirogyrales)	Cytoplasm	GFWN01014285.1	NCBI (TSA) / tBLASTn	0
	Coleochaete orbicularis (Coleochaetales)	Cytoplasm	GBSL01034740.1	NCBI (TSA) / tBLASTn	0
	Klebsormidium flaccidum (Klebsormidiales)	Cytoplasm	GFXY01003449.1	NCBI (TSA) / tBLASTn	0
	Mesostigma viride (Mesostigmatales)	Cytoplasm	GFXX01002596.1	NCBI (TSA) / tBLASTn	0
Chlorophytes	*Chlamydomonas reinhardtii* (Chlorophyceae)	Cytoplasm	XP_042919439.1	NCBI (ClusteredNR) / BLASTP	5e−47
	Volvox carteri (Chlorophyceae)	Cytoplasm	XP_002953715.1	NCBI (ClusteredNR) / BLASTP	0
	Chlorella variabilis (Trebouxiophyceae)	Cytoplasm	XP_005845405.1	NCBI (ClusteredNR) / BLASTP	0
	Chlorella sorokiniana (Trebouxiophyceae)	Cytoplasm	PRW60053.1	NCBI (ClusteredNR) / BLASTP	0
	Micromonas commoda (Prasinophytes)	Cytoplasm	XP_002503812.1	NCBI (ClusteredNR) / BLASTP	0
Rhodophytes	*Chondrus crispus*	Cytoplasm	GKSM01083566.1	NCBI (TSA) / tBLASTn	0
	Porphyra umbilicalis	Cytoplasm	OSX71439.1	NCBI (ClusteredNR) / BLASTP	2e−162
	Porphyridium purpureum	Cytoplasm	KAA8499059.1	NCBI (ClusteredNR) / BLASTP	0
	Rhodosorus marinus	Cytoplasm	KAJ8903637.1	NCBI (ClusteredNR) / BLASTP	0
Glaucophytes	*Cyanoptyche gloeocystis*	Cytoplasm	JKHA_scaffold_2031130	CNGB / tBLASTn	1e−138
	Glaucocystis cf. nostochinearum	Cytoplasm	POOW_scaffold_2010654	CNGB / tBLASTn	0
	Gloeochaete wittrockiana	Cytoplasm	HBOJ01009841.1	NCBI (TSA) / tBLASTn	0

[a]Species lacking allantoin biosynthetic enzymes (ALNS, HIUH, and OHCUD) containing PTS motifs are highlighted in red (see Table S1 for details).
[b]Subcellular localization was predicted using DeepLoc 2.1 (https://services.healthtech.dtu.dk/services/DeepLoc-2.1/).
[c]XDH sequences were retrieved using the indicated databases and programs, with the Arabidopsis XDH1 sequence (NP_195215.2) used as a query. NCBI, National Center for Biotechnology Information; TSA, Transcriptome Shotgun Assembly; EST, Expressed Sequence Tag; CNGB, China National GeneBank.
[d]Formerly known as *Mesotaenium testaceovaginatum*.

Table S3. Occurrence, PTS motifs, and predicted subcellular localization of urate oxidases (UOXs) in Archaeplastida

Major lineage	Species (major group or lineage)[a]	PTS[b]	Subcellular localization[c]	Accession no.	Database / Method[d]	*E*-value
Angiosperms	*Arabidopsis thaliana* (eudicot)	PTS1	Peroxisome	NP_180191.1	NCBI	
	Erigeron breviscapus (eudicot)	PTS1	Peroxisome	GDQF01027466.1	NCBI (TSA) / tBLASTn	6e−159
	Fragaria vesca (eudicot)	PTS1	Peroxisome	GJFN01030541.1	NCBI (TSA) / tBLASTn	2e−160
	Populus trichocarpa (eudicot)	PTS1	Peroxisome	Potri.010G242600.2	Phytozome / BLASTP	2e−164
	Vitis vinifera (eudicot)	PTS1	Peroxisome	GAKH01101673.1	NCBI (TSA) / tBLASTn	1e−160
	Anthurium andraeanum (monocot)	PTS1	Peroxisome	GBKP01033328.1	NCBI (TSA) / tBLASTn	7e−152
	Lilium longiflorum (monocot)	PTS1	Peroxisome	GIUQ01010994.1	NCBI (TSA) / tBLASTn	1e−147
	Oryza sativa (monocot)	PTS1	Peroxisome	GFGS01004669.1	NCBI (TSA) / tBLASTn	8e−142
	Triticum aestivum (monocot)	PTS1	Peroxisome	GEWU01140771.1	NCBI (TSA) / tBLASTn	5e−143
	Zea mays (monocot)	PTS1	Peroxisome	GGGX01010798.1	NCBI (TSA) / tBLASTn	3e−134
	Piper methysticum (Basal angiosperm)	PTS1	Peroxisome	GHAC01204022.1	NCBI (TSA) / tBLASTn	8e−148
Gymnosperms	*Ephedra trifurca* (Gnetidae)	PTS1	Peroxisome	GBKT01034945.1	NCBI (TSA) / tBLASTn	2e−124
	Picea abies (Pinidae)	PTS1	Peroxisome	GKLE01008770.1	NCBI (TSA) / tBLASTn	5e−124
	Picea glauca (Pinidae)	PTS1	Peroxisome	GGJI01011787.1	NCBI (TSA) / tBLASTn	1e−124
	Pinus albicaulis (Pinidae)	PTS1	Peroxisome	GFBO01051450.1	NCBI (TSA) / tBLASTn	7e−124
	Pinus flexilis (Pinidae)	PTS1	Peroxisome	GHWC02108502.1	NCBI (TSA) / tBLASTn	8e−124
	Pinus taeda (Pinidae)	PTS1	Peroxisome	ABD03943.1	NCBI (ClusteredNR) / BLASTP	3e−130
	Araucaria cunninghamii (Cupressidae)	PTS1	Peroxisome	GCKF01015445.1	NCBI (TSA) / tBLASTn	2e−126
	Cryptomeria japonica (Cupressidae)	PTS1	Peroxisome	FX338447.1	NCBI (TSA) / tBLASTn	3e−127
	Sequoia sempervirens (Cupressidae)	PTS1	Peroxisome	GIBU01001078.1	NCBI (TSA) / tBLASTn	7e−126
	Taxus chinensis (Cupressidae)	PTS1	Peroxisome	KAH9317360.1	NCBI (ClusteredNR) / BLASTP	3e−129
	Thuja plicata (Cupressidae)	PTS1	Peroxisome	Thupl.29380577s0001.1	Phytozome / BLASTP	2e−130
	Cycas revoluta (Cycadidae)	PTS1	Peroxisome	GBJU01023793.1	NCBI (TSA) / tBLASTn	6e−133
	Cycas sphaerica (Cycadidae)	PTS1	Peroxisome	GGZS01010180.1	NCBI (TSA) / tBLASTn	4e−129
	Ginkgo biloba (Ginkgoidae)	PTS1	Peroxisome	GGBW01042978.1	NCBI (TSA) / tBLASTn	5e−122
Monilophytes	*Brainea insignis* (Eupolypods)	PTS1	Peroxisome	GFUE01005939.1	NCBI (TSA) / tBLASTn	7e−118
	Dryopteris erythrosora (Eupolypods)	PTS1	Peroxisome	GHEW01041068.1	NCBI (TSA) / tBLASTn	1e−111
	Ceratopteris richardii (Pteridaceae)	PTS1	Peroxisome	HANB01240373.1	NCBI (TSA) / tBLASTn	8e−107
	Pteris vittata (Pteridaceae)	PTS1	Peroxisome	GGXV01062642.1	NCBI (TSA) / tBLASTn	1e−113
	Azolla filiculoides (Salviniaceae)	PTS1	Peroxisome	GBTV01032407.1	NCBI (TSA) / tBLASTn	4e−113
	Lygodium japonicum (Lygodiaceae)	PTS1	Peroxisome	FX959656.1	NCBI (TSA) / tBLASTn	3e−123
	Botrychium lunaria (Ophioglossaceae)	PTS1	Peroxisome	GIJZ01008740.1	NCBI (TSA) / tBLASTn	6e−114
Lycophytes	*Isoetes lacustris* (Isoetaceae)	PTS1	Peroxisome	GIOA01088130.1	NCBI (TSA) / tBLASTn	4e−109
	Selaginella denticulata (Selaginellaceae)	PTS1	Peroxisome	GIMH01018806.1	NCBI (TSA) / tBLASTn	2e−95
	Selaginella moellendorffii (Selaginellaceae)	PTS1	Peroxisome	172716	Phytozome / BLASTP	1e−104

(*continues*)

Table S3. (*continued*)

Major lineage	Species (major group or lineage)[a]	PTS[b]	Subcellular localization[c]	Accession no.	Database / Method[d]	*E*-value
Bryophytes	*Bryum argenteum* (moss)	PTS1	Peroxisome	GCZP01016036.1	NCBI (TSA) / tBLASTn	7e−107
	Dicranum scoparium (moss)	PTS1	Peroxisome	HANF01026949.1	NCBI (TSA) / tBLASTn	3e−112
	Physcomitrella patens (moss)	PTS1	Peroxisome	Pp6c14_8710V6.1	Phytozome / BLASTP	1e−118
	Pohlia nutans (moss)	PTS1	Peroxisome	GACA01009324.1	NCBI (TSA) / tBLASTn	1e−106
	Marchantia paleacea (liverwort)	PTS1	Peroxisome	GFGJ01008364.1	NCBI (TSA) / tBLASTn	3e−112
	Marchantia polymorpha (liverwort)	PTS1	Peroxisome	GEFO01015035.1	NCBI (TSA) / tBLASTn	3e−108
	Anthoceros agrestis (hornwort)	n.d.	Peroxisome	TWUW_scaffold_2049040	CNGB / tBLASTn	2e−101
	Megaceros tosanus (hornwort)	PTS1	Peroxisome	UCRN_scaffold_2051064	CNGB / tBLASTn	7e−107
	Phaeoceros carolinianus (hornwort)	PTS1	Peroxisome	WEEQ_scaffold_2004235	CNGB / tBLASTn	6e−102
	Phaeomegaceros coriaceus (hornwort)	PTS1	Peroxisome	AKXB_scaffold_2065187	CNGB / tBLASTn	2e−105
Streptophyte algae	*Mougeotiopsis calospora* (Serritaeniales)	PTS1	Peroxisome	GJZN01027699.1	NCBI (TSA) / tBLASTn	2e−106
	Serritaenia testaceovaginata[e] (Serritaeniales)	PTS1	Peroxisome	HCDJ01055692.1	NCBI (TSA) / tBLASTn	6e−88
	Cylindrocystis crassa (Zygnematales)	PTS1	Peroxisome	HCDI01046351.1	NCBI (TSA) / tBLASTn	5e−109
	Spirogyra pratensis (Spirogyrales)	PTS1	Peroxisome	GICF01000690.1	NCBI (TSA) / tBLASTn	2e−104
	Coleochaete orbicularis (Coleochaetales)	PTS1	Peroxisome	GBSL01053086.1	NCBI (TSA) / tBLASTn	3e−109
	Klebsormidium flaccidum (Klebsormidiales)	PTS1	Peroxisome	GBSO01023470.1	NCBI (TSA) / tBLASTn	4e−92
	Mesostigma viride (Mesostigmatales)	PTS1	Peroxisome	GBEK01010516.1	NCBI (TSA) / tBLASTn	2e−84
Chlorophytes	*Chlamydomonas reinhardtii* (Chlorophyceae)	PTS1	Peroxisome	Cre12.g504950.t1.2	Phytozome / BLASTP	1e−81
	Volvox carteri (Chlorophyceae)	PTS1	Peroxisome	Vocar.0016s0304.1	Phytozome / BLASTP	1e−86
	Chlorella variabilis (Trebouxiophyceae)	PTS1	Peroxisome	XP_005850936.1	NCBI (ClusteredNR) / BLASTP	5e−72
	Chlorella sorokiniana (Trebouxiophyceae)	n.d.	Peroxisome	GAPD01005016.1	NCBI (TSA) / tBLASTn	3e−74
	Micromonas commoda (Prasinophytes)	PTS1	Peroxisome	XP_002506401.1	NCBI (ClusteredNR) / BLASTP	6e−73
Rhodophytes	*Chondrus crispus*	PTS1	Peroxisome	XP_005710064.1	NCBI (ClusteredNR) / BLASTP	1e−76
	Porphyridium purpureum	PTS1	Peroxisome	KAA8496926.1	NCBI (ClusteredNR) / BLASTP	6e−78
	Rhodosorus marinus	PTS1	Peroxisome	KAJ8908107.1	NCBI (ClusteredNR) / BLASTP	2e−67
Glaucophytes	*Cyanoptyche gloeocystis*	PTS1	Peroxisome	JKHA_scaffold_2027464	CNGB / tBLASTn	1e−59
	Glaucocystis cf. nostochinearum	PTS1	Peroxisome	POOW_scaffold_2057635	CNGB / tBLASTn	4e−55
	Gloeochaete wittrockiana	PTS1	Peroxisome	HBOJ01051872.1	NCBI (TSA) / tBLASTn	3e−59

[a]Species lacking allantoin biosynthetic enzymes (ALNS, HIUH, and OHCUD) containing PTS motifs are highlighted in red (see Table S1 for details).
[b]PTS motifs were predicted using PTS1 Predictor (https://mendel.imp.ac.at/pts1/); PTS2 was assessed manually (not detected). When present, PTS1 is located at the carboxyl terminus of UOX. n.d., not detected.
[c]Subcellular localization was predicted using DeepLoc 2.1 (https://services.healthtech.dtu.dk/services/DeepLoc-2.1/).
[d]UOX sequences were retrieved using the indicated databases and programs, with the Arabidopsis sequence (NP_180191.1) used as a query. NCBI, National Center for Biotechnology Information; TSA, Transcriptome Shotgun Assembly; EST, Expressed Sequence Tag; CNGB, China National GeneBank.
[e]Formerly known as *Mesotaenium testaceovaginatum*.

Table S4. Molecular docking of (*S*)- and (*R*)-allantoin to the putative substrate-binding region of Arabidopsis allantoinase (AtALN)

Ligand	Model	AutoDock Vina score (kcal mol^{-1})	Minimum distances to putative substrate-binding residues (Å)[a,b]		
			Asn140	Asp366	Ser368
(*S*)-allantoin	1	−6.354	5.12	2.90	3.49
	2	−5.944	3.22	3.13	2.95
	3	−5.919	4.69	2.90	3.19
	4	−5.893	2.86	3.15	3.05
	5	−5.859	6.01	3.02	3.24
(*R*)-allantoin	1	−6.070	2.95	3.19	3.17
	2	−6.057	4.84	3.08	3.34
	3	−6.039	3.10	3.19	3.03
	4	−5.986	3.03	3.14	2.93
	5	−5.832	4.65	3.11	3.25

Molecular docking was performed using SwissDock (https://www.swissdock.ch/) with AutoDock Vina and the AlphaFold-predicted structure of AtALN (UniProt Q94AP0; AF-Q94AP0-F1), under identical conditions for both enantiomers. See Figure S7 for the corresponding docking poses.

[a]Minimum distances indicate the shortest heavy-atom distances between each docking pose of allantoin and the side chain of the indicated residue.

[b]Asn140, Asp366, and Ser368 are conserved AtALN residues corresponding to substrate-interacting residues identified in a bacterial allantoinase (Asn94, Asp315, and Ser317, respectively; Kim *et al*., 2009).

Table S5. PCR primers used in this study

AGI code[a]	Gene symbol[b]	Direction	Sequence (5´ to 3´)	Use
AT5G58220	*ALNS*	Forward	AAGGGTGGGCGCGCCGAC	ALNSpro:AtALNS-L or ALNSpro:AtALNS-S construction (to amplify the pENTR/D TOPO sequence)
		Reverse	AAGGGGGCGGCCGCGGAG	
		Forward	GGCTCCGCGGCCGCCCCCTTGCAACACTGGAAATAACCCAATC	ALNSpro:AtALNS-L, ALNSpro:AtALNS-S, or ALNSpro:gAtALNS construction (to amplify the *AtALNS* promoter)
		Reverse	CCATCGCCATTTTTTCTCTCTTTTCTCCAATCCTC	
		Forward	GAGAGAAAAAATGGCGATGGAGATCGGAGA	ALNSpro:AtALNS-L, ALNSpro:AtALNS-S, or ALNSpro:gAtALNS construction (to amplify *AtALNS* cDNA or genomic sequence)
		Reverse	GGGTCGGCGCGCCCACCCTTAGCTCCCACGGTATGTGGA	ALNSpro:AtALNS-L or ALNSpro:AtALNS-S construction (to amplify an *AtALNS* cDNA sequence)
		Reverse	GGGTCGGCGCGCCCACCCTTCTAGCTCCCACGGTATGTGGA	ALNSpro:gAtALNS construction (to amplify the *AtALNS* genomic sequence)
		Forward	CACCATGGCGATGGAGATCGGA	35Spro:EGFP–AtALNS-L, 35Spro:EGFP–AtALNS-S, or 35Spro:EGFP–gAtALNS construction (for inverse PCR)
		Reverse	AAGGGGGCGGCCGCGGAG	
		Forward	GTGGAGCTTGAGATTGCTGCTATGGA	PCR genotyping (for *alns-1*; indicated as black arrows in Figure S4A)
		Reverse	GCCCTCCAATGATTCTCAGACG	
		Forward	AGCGGTCATCATCGTACTGAC	PCR genotyping (for *alns-2*; indicated as black arrows in Figure S9A)
		Reverse	TTGGAAATGCAAAAGTTGACC	
		Forward	GCTCTCCTGTTTCAACAAAACC	Conventional RT-PCR (in *alns-1* background; indicated as red arrows in Figure S4A)
		Reverse	GGATTCAAAGCGTCAACCAAATC	
		Forward	GGAGATCGGAGAAGATGAATGGA	Conventional RT-PCR (in *alns-2* background; indicated as red arrows in Figure S9A)
		Reverse	AAAGGCGGTTGACTGTTCTG	
		Forward	CGTCGGAGCGTATCAGAACA	Quantitative RT-PCR (indicated as blue arrows in Figure S4A)
		Reverse	CCATTCGGCAAGCTCCTGTA	
AT4G34890	*XDH1*	Forward	GACAAGCACTGACAAGGTTCC	Quantitative RT-PCR
		Reverse	GCGTCTAAAACTGCAGCACC	
AT2G26230	*UOX*	Forward	AAGCTTGGGTCAGAGAACCAT	Quantitative RT-PCR
		Reverse	ACCTCTCAAATCCTGACTGGG	
AT4G04955	*ALN*	Forward	CCTGGTCTCATTGATGTGCATGTTC	Quantitative RT-PCR
		Reverse	TGTTTTCGCAGCTTCAATCTTGAGT	
AT4G20070	*AAH*	Forward	CTTTTGTGCTCCATTGAACGAAAGC	Quantitative RT-PCR
		Reverse	AACAACATTCCCACCTTGGTTAAGTGT	
AT2G28390	*SAND*	Forward	CAGACAAGGCGATGGCGATA	Conventional RT-PCR (as a reference)
		Reverse	GCTTTCTCTCAAGGGTTTCTGGGT	
AT4G27960	*UBC9*	Forward	TCACAATTTCCAAGGTGCTGC	Quantitative RT-PCR (as a reference)
		Reverse	TCATCTGGGTTTGGATCCGT	
AT1G62930	*PPR*	Forward	GAGTTGCGGGTTTGTTGGAG	Quantitative RT-PCR (as a reference)
		Reverse	CAAGACAGCATTTCCAGATAGCAT	
—	T-DNA	(LBa1)	TGGTTCACGTAGTGGGCCATCG	PCR genotyping (for T-DNA insertion lines)
		(LB1)	GCCTTTTCAGAAATGGATAAATAGCCTTGCTTCC	

[a]*Arabidopsis thaliana* gene locus identifiers assigned by the Arabidopsis Genome Initiatives (AGI) according to the Arabidopsis gene nomenclature guidelines (https://phoenixbioinformatics.atlassian.net/wiki/spaces/COM/pages/42215945/Arabidopsis+Nomenclature).
[b]As provided by The Arabidopsis Information Resource (TAIR; release 10; https://www.arabidopsis.org/), except for *Agrobacterium tumefaciens* T-DNA.

Supporting Methods

Experimental procedures are described in the relevant section of the main text, except as noted below.

S1. Protein extraction, immunoprecipitation, and immunoblotting

Total proteins were extracted from the samples using lysis buffer (10 mM Tris–HCl, pH 7.5, 150 mM NaCl, 0.5 mM EDTA, 0.5% [v/v] Nonidet P-40 substitute, 0.09% [w/v] sodium azide, and 1 mM phenylmethanesulfonyl fluoride). The lysate was centrifuged at 20,000 × *g* for 5 min at 4°C. An aliquot of the supernatant was retained as input, and the remaining supernatant was subjected to immunoprecipitation (IP) with anti-GFP agarose beads (50 μL bead slurry per mg of total protein; ChromoTek, Planegg-Martinsried, Germany) for 1 h at 4°C with gentle rotation. The beads were washed twice with wash buffer (10 mM Tris–HCl, pH 7.5, 150 mM NaCl, 0.5 mM EDTA, 0.05% [v/v] Nonidet P-40 substitute, and 0.018% [w/v] sodium azide). Input samples and bead-bound proteins were resuspended in loading buffer (50 mM Tris–HCl, pH 6.8, 2% [w/v] SDS, 5% [w/v] sucrose, 5% [v/v] 2-mercaptoethanol, and a trace amount of bromophenol blue) and boiled for 5 min prior to SDS–PAGE.

Proteins were separated by SDS–PAGE using a 10% (w/v) polyacrylamide gel and transferred onto a polyvinylidene difluoride membrane (Immobilon-P; Millipore, Billerica, MA, USA). The membrane was blocked with 5% (w/v) skim milk for 1 h at room temperature and incubated with an anti-GFP antibody conjugated to horseradish peroxidase (1:5,000 dilution; Proteintech, Rosemont, IL, USA) for 1 h at room temperature. Chemiluminescent detection was performed using a Western Lightning Plus-ECL kit (Revvity, Waltham, MA, USA), and signals were visualized using a ChemiDoc MP Imaging System (Bio-Rad, Hercules, CA, USA).

S2. Conventional and quantitative reverse-transcription PCR

Total RNA was extracted from the samples using a NucleoSpin RNA II Kit (Macherey-Nagel, Düren, Germany), and 500 ng of total RNA was reverse-transcribed using a ReverTra Ace qPCR RT Kit (Toyobo, Osaka, Japan). To detect *ALNS* transcripts by conventional reverse-transcription PCR (RT-PCR), reactions were performed in 20-μL volumes containing 10 ng of cDNA, using *Taq* DNA polymerase (New England Biolabs, Ipswich, MA, USA) on a T100 Thermal Cycler (Bio-Rad). The thermal cycling conditions were 95°C for 3 min, followed by 30 cycles of 95°C for 30 s, 55°C for 30 s, and 68°C for 30 s, with a final extension at 68°C for 5 min for the *alns-1* mutant and its complementation lines (Figure S5). For the *alns-2* mutant, the cycle number was increased to 35, and the annealing temperature was increased to 63°C (Figure S9). *SAND FAMILY PROTEIN* (*SAND*; locus AT2G28390) was used as an internal control.

Quantitative RT-PCR was performed essentially as described by Han *et al*. (2020), using 10 ng of cDNA per 20-μL reaction with a KAPA SYBR FAST qPCR Kit (Kapa Biosystems, Inc., Woburn, MA, USA) on a Roche LightCycler 96 (Roche Diagnostics, Basel, Switzerland). The thermal cycling conditions were 95°C for 3 min, followed by 40 cycles of 95°C for 15 s and 65°C for 40 s. Melting curve analysis was performed from 65°C to 95°C at 0.5°C increments. The relative expression levels of target genes were calculated using the comparative C_T method (Schmittgen and Livak, 2008), with *E2 UBIQUITIN-CONJUGATING ENZYME 9* (*UBC9*; locus AT4G27960) or *PENTATRICOPEPTIDE REPEAT* (*PPR*; locus AT1G62930) used as reference transcripts. The primer sequences used in these analyses are listed in Table S5.

S3. Molecular docking analysis

Molecular docking of (*S*)- and (*R*)-allantoin to Arabidopsis allantoinase (AtALN) was performed using SwissDock with the AutoDock Vina engine (https://www.swissdock.ch/). The AlphaFold-predicted structure of AtALN (UniProt: Q94AP0; AF-Q94AP0-F1) served as the receptor. The grid box was defined to cover the putative substrate-binding region, including Asn140, Asp366, and

Ser368, which correspond to the substrate-interacting residues identified in a bacterial allantoinase (Kim *et al*., 2009), as well as the adjacent catalytic metal center. The box was centered at x = 6.7, y = 3.6, and z = −4.4 Å, with dimensions of 18 × 18 × 18 Å. The exhaustiveness parameter was set to 32, and identical docking parameters were applied to both enantiomers. The top five poses for each enantiomer, ranked by AutoDock Vina score, were selected to assess proximity to Asn140, Asp366, and Ser368. Minimum distances were determined as the shortest heavy-atom distance between each allantoin pose and the side chain of the indicated AtALN residue. Docking poses were visualized and figures were prepared using PyMOL (version 3.1.8; Schrödinger, LLC, New York, NY, USA).

S4. Flowering time measurements

Wild-type (WT) plants and two allelic *alns* mutants, *alns-1* and *alns-2*, were germinated and grown at 23°C under long-day conditions. Flowering time was determined following Domagalska *et al.* (2007) as the number of days after germination and the total number of rosette leaves when the primary inflorescence stem reached approximately 10 mm in height.

Supporting References

Domagalska, M.A., Schomburg, F.M., Amasino, R.M., Vierstra, R.D., Nagy, F. & Davis, S.J. (2007) Attenuation of brassinosteroid signaling enhances *FLC* expression and delays flowering. *Development* **134**, 2841–2850. Available from: https://doi.org/10.1242/dev.02866

's-Gravenmade, E.J., Vogels, G.D. & van Pelt, C. (1969) Preparation, properties and absolute configuration of (–)-allantoin. *Recueil des Travaux Chimiques des Pays-Bas* **88**, 929–939. Available from: https://doi.org/10.1002/recl.19690880806

Han, Y., Watanabe, S., Shimada, H. & Sakamoto, A. (2020) Dynamics of the leaf endoplasmic reticulum modulate β-glucosidase-mediated stress-activated ABA production from its glucosyl ester. *Journal of Experimental Botany* **71**, 2058–2071. Available from: https://doi.org/10.1093/jxb/erz528

Hayashi, Y., Yamada, K., Shimada, T., Matsushima, R., Nishizawa, N.K., Nishimura, M. & Hara-Nishimura, I. (2001) A proteinase-storing body that prepares for cell death or stresses in the epidermal cells of Arabidopsis. *Plant and Cell Physiology* **42**, 894–899. Available from: https://doi.org/10.1093/pcp/pce144

Ivanov, S. & Harrison, M.J. (2014) A set of fluorescent protein-based markers expressed from constitutive and arbuscular mycorrhiza-inducible promoters to label organelles, membranes and cytoskeletal elements in *Medicago truncatula*. *The Plant Journal* **80**, 1151–1163. Available from: https://doi.org/10.1111/tpj.12706

Kechasov, D., de Grahl, I., Endries, P. & Reumann, S. (2020) Evolutionary maintenance of the PTS2 protein import pathway in the stramenopile alga *Nannochloropsis*. *Frontiers in Cell and Developmental Biology* **8**, 593922. Available from: https://doi.org/10.3389/fcell.2020.593922

Kim, K., Kim, M.-I., Chung, J., Ahn, J.-H. & Rhee, S. (2009) Crystal structure of metal-dependent allantoinase from *Escherichia coli*. *Journal of Molecular Biology* **387**, 1067–1074. Available from: https://doi.org/10.1016/j.jmb.2009.02.041

Kunze, M. (2020) The type-2 peroxisomal targeting signal. *Biochimica et Biophysica Acta – Molecular Cell Research* **1867**, 118609. Available from: https://doi.org/10.1016/j.bbamcr.2019.118609

Lamberto, I., Percudani, R., Gatti, R., Folli, C. & Petrucco, S. (2010) Conserved alternative splicing of *Arabidopsis* transthyretin-like determines protein localization and *S*-allantoin synthesis in peroxisomes. *The Plant Cell* **22**, 1564–1574. Available from: https://doi.org/10.1105/tpc.109.070102

Martín, G., Márquez, Y., Mantica, F., Duque, P. & Irimia, M. (2021) Alternative splicing landscapes in *Arabidopsis thaliana* across tissues and stress conditions highlight major functional differences with animals. *Genome Biology* **22**, 35. Available from: https://doi.org/10.1186/s13059-020-02258-y

Nakagawa, T., Kurose, T., Hino, T., Tanaka, K., Kawamukai, M., Niwa, Y., Toyooka, K., Matsuoka, K., Jinbo, T. & Kimura, T. (2007) Development of series of gateway binary vectors, pGWBs, for realizing efficient construction of fusion genes for plant transformation. *Journal of Bioscience and Bioengineering* **104**, 34–41. Available from: https://doi.org/10.1263/jbb.104.34

Ogasawara, Y., Ishizaki, K., Kohchi, T. & Kodama, Y. (2013) Cold-induced organelle relocation in the liverwort *Marchantia polymorpha* L. *Plant, Cell & Environment* **36**, 1520–1528. Available from: https://doi.org/10.1111/pce.12085

Oh, J., Liuzzi, A., Ronda, L., Marchetti, M., Corsini, R., Folli, C., Bettati, S., Rhee, S. & Percudani, R. (2018) Diatom allantoin synthase provides structural insights into natural fusion protein therapeutics. *ACS Chemical Biology* **13**, 2237–2246. Available from: https://doi.org/10.1021/acschembio.8b00404

Ramazzina, I., Folli, C., Secchi, A., Berni, R. & Percudani, R. (2006) Completing the uric acid degradation pathway through phylogenetic comparison of whole genomes. *Nature Chemical Biology* **2**, 144–148. Available from: https://doi.org/10.1038/nchembio768

Reumann, S., Babujee, L., Ma, C., Wienkoop, S., Siemsen, T., Antonicelli, G.E., Rasche, N., Lüder, F., Weckwerth, W. & Jahn, O. (2007) Proteome analysis of Arabidopsis leaf peroxisomes reveals novel targeting peptides, metabolic pathways, and defense mechanisms. *The Plant Cell* **19**, 3170–3193. Available from: https://doi.org/10.1105/tpc.107.050989

Schmittgen, T.D. & Livak, K.J. (2008) Analyzing real-time PCR data by the comparative C_T method. *Nature Protocols* **3**, 1101–1108. Available from: https://doi.org/10.1038/nprot.2008.73
